# Massively Parallel RNA Sequencing Identifies a Complex Immune Gene Repertoire in the lophotrochozoan *Mytilus edulis*

**DOI:** 10.1371/journal.pone.0033091

**Published:** 2012-03-20

**Authors:** Eva E. R. Philipp, Lars Kraemer, Frank Melzner, Albert J. Poustka, Sebastian Thieme, Ulrike Findeisen, Stefan Schreiber, Philip Rosenstiel

**Affiliations:** 1 Institute of Clinical Molecular Biology, Christian-Albrechts-University Kiel, Kiel, Germany; 2 Environmental Physiology, Helmholtz Centre for Ocean Research Kiel, Kiel, Germany; 3 Evolution and Development Group, Max-Planck Institute for Molecular Genetics, Berlin, Germany; Northeastern University, United States of America

## Abstract

The marine mussel *Mytilus edulis* and its closely related sister species are distributed world-wide and play an important role in coastal ecology and economy. The diversification in different species and their hybrids, broad ecological distribution, as well as the filter feeding mode of life has made this genus an attractive model to investigate physiological and molecular adaptations and responses to various biotic and abiotic environmental factors. In the present study we investigated the immune system of *Mytilus*, which may contribute to the ecological plasticity of this species. We generated a large *Mytilus* transcriptome database from different tissues of immune challenged and stress treated individuals from the Baltic Sea using 454 pyrosequencing. Phylogenetic comparison of orthologous groups of 23 species demonstrated the basal position of lophotrochozoans within protostomes. The investigation of immune related transcripts revealed a complex repertoire of innate recognition receptors and downstream pathway members including transcripts for 27 toll-like receptors and 524 C1q domain containing transcripts. NOD-like receptors on the other hand were absent. We also found evidence for sophisticated TNF, autophagy and apoptosis systems as well as for cytokines. Gill tissue and hemocytes showed highest expression of putative immune related contigs and are promising tissues for further functional studies. Our results partly contrast with findings of a less complex immune repertoire in ecdysozoan and other lophotrochozoan protostomes. We show that bivalves are interesting candidates to investigate the evolution of the immune system from basal metazoans to deuterostomes and protostomes and provide a basis for future molecular work directed to immune system functioning in *Mytilus*.

## Introduction

The marine mussel *Mytilus edulis* and its closely related sister species *M. galloprovincialis*, *M. trossulus*, *M. californianus*, *M. chilensis* and *M. coruscus* are distributed world-wide and play an important role in coastal ecology and economy. The genus' diversification and broad ecological distribution has made these species attractive models to investigate physiological and molecular adaptations to different environmental conditions. Especially the understanding of the immune system has been a focus of research in *Mytilus* spp., in order to improve aquaculture conditions, but also to understand specific acclimation and adaptation processes to local and changing environmental settings [1], [2]. The immune system of *Mytilus* has so far been mainly studied on the cellular and protein level [3], [4], but recent investigations on the molecular level already gave indications about the complexity and diversification of the innate immune system [2], [5], [6]. Filter feeding bivalves such as *Mytilus spp.*, are constantly exposed to a complex microbiota from the surrounding seawater medium and thus subject to extensive attack of potential pathogens (e.g. *Vibrio* species). However, reports of mass mortality are lacking in *Mytilus spp.* and infections by pathogens are rarely reported [7], which speaks for an effective immune system in this genus. In the present paper we report a large *Mytilus edulis* transcriptome (2.39mio reads assembled into 74,622 contigs and 176,476 singletons), generated from different tissues of immune challenged and stress treated *M. edulis* individuals from the Baltic Sea using 454 pyrosequencing. In the subsequent analysis of the *M. edulis* database the stringently assembled contigs were searched for immune related transcripts with a special focus on the comparison to well-known immune related genes of other invertebrates and vertebrates. The rationale behind this approach is the currently changing understanding of phylogenetic relationships and complexity of cellular pathways in animal phyla, due to the increasing availability of sequences from massive parallel sequencing projects. Sequencing of genomes and transcriptomes of invertebrates like the sea urchin *Strongylocentrotus purpuratus* [8], the leech *Hirudo medicinalis* [9] or Cnidarians [10] as well as basal chordates like *Branchiostoma floridae* [11] revealed an unexpected diversity of innate immune system pathways and even primitive precursors of an adaptive immune system. Several immune system components which were thought to be restricted to the vertebrate clade were found to already occur in lower invertebrates and were lost again during evolution in the classical invertebrate model organisms *C. elegans* and *D. melanogaster* [12], [13], [14]. While genomes and transcriptomes of the ecdysozoan clade, as well as lower invertebrates of the deuterostome clade have been extensively investigated in the past, detailed sequence information of the third bilaterian clade, the lophotrochozoans, is still lacking. However, this clade might be especially useful for the understanding of evolutionary conserved pathways as there is increasing evidence that gene divergence and loss in the lophotrochozoan clade occurred at a lower rate compared to ecdysozoans [12]. Thus this group might reveal interesting insights into the function and evolution of the immune system. In the present study *Mytilus edulis* was used as a model species for the lophotrochozoan clade to a) investigate the utility of bivalves to understand the evolution of the immune system from basal metazoans to deuterostomes and protostomes and b) to lay a basis for future molecular work directed to immune system functioning in *Mytilus spp*.

## Methods

### Sample collection and preparation

Several independent transcriptomal data sets of *M. edulis* were generated using 454 technology (454 Life Sciences, USA) with FLX and Titanium chemistry. To maximize the diversity of expressed stress- and immune-related transcripts in the experimental material, *M. edulis* individuals were exposed to different stressors and different tissues were used for RNA extraction. For the first set of transcriptome sequencing, *M. edulis* individuals were collected in March 2008 in Kiel Fjord (Germany) in front of the Helmholtz Centre for Ocean Research Kiel (GEOMAR). Animals were cleaned from epifauna and kept in a constant temperature room at 10°C with a flow-through of natural unfiltered seawater from Kiel Bay. After 1 week of acclimation, animals were exposed to the different experimental conditions (5 animals each): 5 days high temperature (20°C), anoxia: wrapped in a double layer of parafilm at 10°C and injury: cracking the shell, and 4 hours immune system challenge: injection of 10 µg lipopolysaccharide (kindly provided by Prof. U. Zaehringer, Research Center Borstel, Germany). Control animals were kept at 10°C and a salinity of 17 in flow-through seawater. Two animals of each experiment were dissected and the different tissues frozen in liquid nitrogen. From these experiments, digestive gland, gill, foot and adductor muscle was used for sequence generation. To enhance the number of stress related transcripts, sequencing reads of ongoing immune and stress related studies were included in the database. This included digestive gland tissues of 4 *Mytilus edulis* individuals in which inflammation was chemically induced by dextran sodium sulfate (2–4%, 5days; TdB Consultancy, Sweden) and 4 control individuals without DSS treatment, mantle tissues of animals acclimated to high (4000 µatm) and low (380 µatm) seawater pCO_2_ levels (8–12 animals each, 8 week acclimation, samples from Thomsen and Melzner [15]), as well as *M. edulis* hemocytes pooled from 32 individuals and treated for 4 h *in vitro* with or without 2.5 µg/ml flagellin (Enzo Life Sciences GmbH, Germany). The different samples were ground in liquid nitrogen and total RNA extracted using the Qiagen RNeasy (Qiagen, Germany) kit in combination with the QiaShredder from Qiagen. mRNA was purified by using the Qiagen Oligotex mRNA purification kit (Qiagen, Germany). In case of pooled samples equal amounts of mRNA were merged and 400–600 ng used for cDNA generation with the SMART cDNA synthesis kit (Clontech Laboratories, USA). Quality control in each extraction step was investigated using gel electrophoresis and a nanodrop spectrophotometer (Peqlab, Germany). cDNA was further checked with a Bioanalyzer 2100 (Agilent Technologies, USA).

### 454 sequencing, EST assembly

Samples were prepared for 454 sequencing according to the manufacturers protocol and sequenced on a 454 Genome Sequencer system (Roche Life Sciences, USA) with FLX and Titanium chemistry (see Table S1 for details of sample sets used for the different transcriptome runs). cDNA libraries used for sequencing were not normalized as recent papers show that although normalization reduces the amount of highly expressed genes, such as ribosomal or genes of structural proteins, the number of genes and GO terms is however not necessarily improved by normalization [16], [17]. Moreover after this first investigation about the immune inventory of *M. edulis*, further investigations will focus on differential expression analysis between tissues and treatments which require non- normalized datasets.

The resulting sequences were extracted from 454 output files using the ‘sffinfo’ script from Roche. Subsequently sequences were again quality controlled (bases with quality 10≤ were removed) and cleaned for primer and adapter sequences (Smart primer sequences and 454 adapters) as well as polyA tails by ‘seqclean’, ‘cln2qual’ (TGI - The Gene Index Project) before the assembly. Reads smaller than 40 bp after the quality control and cleaning were excluded from further sequence assembly and annotation. Details about sequence data input are given in file S1. The trimmed reads of the first data set were assembled with the Celera assembler version 5.3 and subsequently in several rounds with the TGICL (Cap3) assembler. The ‘minimum overlap length’ varied between 40 and 200 bp and the ‘minimum overlap identity’ between 90 and 100%. Additional reads from the CO_2_ treated mantle tissue and flagellin stimulated hemocytes were assembled with the ‘GS De novo Assembler 2.3’ from Roche (NEWBLER, Roche/454 Life Sciences), as a first step. The standard parameters of the ‘GS De novo Assembler 2.3’ were used for this initial assembly (‘minimum overlap length’ = 40; ‘minimum overlap identity’ = 90). Afterwards the resulting contigs and singletons were further assembled in multiple rounds using the TGICL (Cap3) assembler, using the first transcriptome as a backbone. The ‘minimum overlap length’ varied between 40 and 460 bp and the ‘minimum overlap identity’ between 80 and 98%. At last all reads were mapped against the generated contigs and the *Mytilus edulis* mitochondrion genome (GI:55977238) using AMOScmp (http://sourceforge.net/apps/mediawiki/amos/index.php? title = AMOScmp), which resulted in the final contigs. A detailed list of the assembly process is given in table S2.

### Database statistics

For comparison with published *Mytilus* sequence information, the obtained contigs and singletons were blasted (BLASTN) against already published *Mytilus* sequences with a cut off e-value of 1e ^−^10, 20, 30, 60 and 100. *M. edulis* and *M. galloprovincialis* sequence reads generated by pyrosequencing (454 Roche, USA) by Craft et al. [18] were downloaded from the MG-RAST portal (http://metagenomics.nmpdr.org/; [19]), cleaned and assembled as described above. In total 7088 MG-RAST *M. edulis* sequences (average length 147 bp) could be assembled into 271 contigs (av. length 245 bp) and 5742 singletons (av. length 138 bp). The 104123 MG-RAST sequences (av. length 211 bp) of *M. galloprovincialis* were assembled into 12,827 contigs (av. length 279 bp) and 40,972 singletons (av. length 207 bp). Additional 5036 *M. edulis* and 12827 *M. galloprovincialis* as well as all sequences belonging to the genus *Mytilus* (67779 sequences) were downloaded from the NCBI database (http://www.ncbi.nlm.nih.gov, 12/2010), which in case of *M. galloprovincialis* also includes the sequences obtained by Mytibase (http://mussel.cribi.unipd.it/, [20]). These sequences were not assembled but used as singletons due to their different origin with respect to investigators and sequencing techniques.

### Phylogenetic analysis

Orthologous groups of 23 species (Table S3) were computed by bidirectional best hit (BBH) method [21] and manually inspected. To select one-to-one orthologs from the *Mytilus edulis* EST catalog we used BLAST against the pre-computed groups and additional manual inspection. Missing data were not allowed. 71 gene groups fulfilled this criterion. Alignments were computed using MUSCLE [22] on all identified groups individually. Ambiguously aligned regions were detected and removed with Gblocks [23] (b2 : 16, b3 : 5, b4 : 5, b5 : h). To increase the phylogenetic signal, supermatrices were established by concatenating the processed single gene alignments using an in-house script. The length of the final concatenated alignment is 30784 Amino acid positions (file S1, S2). The phylogenomic dataset was analyzed with PhyloBayes version 2.3 [24] using the CAT-GTR+Γ_4_ mixture model. Two independent chains were run for 10000 cycles. For construction of the consensus tree between the two chains a burn in of 1000 trees was used. A maximum difference of 0.0032048 and a mean difference of 2.7709e-05 across all observed bipartitions of the compared independent chains was obtained. Species and gene sequence sources: We used peptide sequences of gene predictions from the completed genomes downloaded as indicated in table S3. The unpublished genomes of *Capitella teleta*, *Lottia gigantea* and *Helobdella robusta* were produced by the US Department of Energy Joint Genome Institute (JGI) http://www.jgi.doe.gov/ in collaboration with the user community. We thank the Acorn Worm Genome Sequencing Consortium for making their data publicly available, and the BCM-HGSC for providing the draft assembly (Skow_1.0) and the JGI for the peptide sequences of the gene predictions. The *Schmidtea mediterannea* data were produced by the Genome Institute at Washington University School of Medicine in St. Louis and were obtained from ftp://genome.wustl.edu/pub/organism/Invertebrates/Schmidtea_mediterannea/assembly/Schmidtea_mediterranea-3.1/output/. We thank the Broad institute to make *Aplysia californica* data publicly available.

### Annotation of *M. edulis* sequences

Putative gene names and protein domains were assigned to all assembled sequences and singletons of the *M. edulis* transcriptome by using the BLASTX algorithm against the UniprotKB/Swissprot database of UniProt Knowledgebase (UniProtKB, http://www.expasy.org/sprot/) with a cut off e-value of e ≤10^−3^, as well as tBLASTx (e≤10^−3^) and BLASTn (e≤10^−10^) against the NCBI nt database (http://www.ncbi.nlm.nih.gov). To identify conserved domains, the assembled contigs were run via InterProScan [25]. Gene Ontology (GO) terms were deduced from the BLAST and InterProScan results and sorted into the immediate subcategories for ‘molecular function’, ‘cellular component’ and ‘biological process’.

### Identification of immune related transcripts

Contigs and associated annotations were analyzed in T-ACE [26]. Orthologs of immune genes were searched via key words and protein structure in the established *M. edulis* transcriptome. Identified transcripts were visually inspected and re-blasted by hand via nr/nt (tBLASTx, NCBI) against *Homo sapiens* (taxid 9606), *Drosophila melanogaster* (taxid 7227), *Caenorhabditis elegans* (taxid 6239), *Mus musculus* (taxid 10090), *Molluscs* (taxid 6447), *Bivalvia* (taxid 6544) and *Mytilus* (taxid 6548). Protein domains were re-evaluated using SMART [27] and NCBI DART [28], and transmembrane domains specifically analyzed with the TMHMM Server v. 2.0 (www.cbs.dtu.dk/services/TMHMM-2.0/). TNF domains were additionally re-checked within Prosite (http://www.expasy.org/prosite/). In case of the high number of contigs identified for large gene families (e.g. TNF, C1q), contigs were loaded into Sequencer 4.5 and manually assessed whether the sequences assembled with min 20 bp overlap and 90% identity or indeed represent separate gene fragments.

### Tissue expression of selected contigs with putative immune function

A selected number of putative *M. edulis* immune gene encoding contigs were investigated in a tissue panel of untreated *M. edulis* individuals to identify tissues of high immune-competence which could serve as candidate tissues for future functional studies. *M. edulis* individuals of equal size were sampled in September 2010 and kept in a flow through system at 15°C for 1 week. Expression of selected transcripts (Table 1) was investigated in gill, foot, digestive gland, adductor muscle, inner mantle tissue (central zone), mantle rim (pallial and marginal zone) and hemocytes. Prior to tissue dissection hemolymph of six animals was collected individually with a syringe from the posterior adductor muscle, immediately centrifuged at 400×g, for 10 min at 15°C, and the pellet frozen in liquid nitrogen. Subsequently the six animals were dissected and tissues immediately frozen in liquid nitrogen. Total RNA was extracted as described above and quality and quantity determined using a nanodrop spectrophotometer (Peqlab, Erlangen). Equal amounts of total RNA from the six different individuals per tissue were pooled for cDNA generation using the Advantage RT-for PCR Kit (Clontech, Germany). Real-time primer (Table 1) were designed using Primer3 [29] and investigated for primer-dimers and hairpin formation using netprimer (Premier Biosoft). To check primer specificity, a PCR was performed with Advantage Taq 2 polymerase (Clontech, Germany) and the appropriate PCR conditions. The PCR products were evaluated by gel electrophoresis to confirm the presence and size of the transcripts. After extraction and purification from the gel PCR products were Sanger sequenced using Big Dye Terminator (BDT) Chemistry (Applied Biosystems, USA). Resulting sequences were assembled with the respective initial contig using Sequencher version 4.5 (GeneCodes, USA).

**Table 1:** Primer sequences used for qRT-PCR analysis of tissue specific expression of selected contigs with putative immune function identified by BLAST and domain analysis in *Mytilus edulis*.

Gene name	*M. edulis* Accession	5′-3′forward primer	5′-3′reverse primer
*Me2-*IRF	HE609043	TGAACGACAGCGAATGAGAC	GCTTGATGGCGAGGATGTTA
*Me3*-IRF	HE609044	GACGCTGATTTGTTTGAACG	ATCCTCCGACATTTCCTCCT
*Me4*-IRF	HE609045	GGAACCTGACCTTGGTGTTG	TGATGGCGTCCTGTATGAAA
LITAF	HE609047	GAGCCACCCTCCAAACAG	ACCAACCTCCACCAGCCTAT
NF-κB p65	HE609048	GACTTCATCCCCGCCTTT	ACCCCATCCACATAACCTTG
*Me1*- NF-κB p50	HE609049	TGATGAAATGAAAGAAGAAGAAAAA	TGGCGGTGTGTAGAGGTAGA
*Me1*_I*κ*B	HE609059	GAGACACACCCTTACACATCG	TCCCACATTTACCATCCTTGA
*Me2*_I*κ*B	HE609060	GCCGCAATAAAACTGAAACC	GGAAAAGACGAGGATGGTGA
IRAK 4	HE609065	CCCGTTTACTTCCGTGTTGT	TTTGTGTTCCCCAGTCTTCA
*Me1*-LBP	HE609067	CGGTTCAGTTTTCAGGGATG	TCTTGCTGTCTGTGATTGGTG
Myd88	HE609071	GCCCTCTCGTCCCAAGTAT	GGGTTCATCCATTCAACACAG
TRAF6	HE609075	CTGCTGTCCCACAATCACAC	AGCCCCACCTTCTACATCCT
*Me2-*PGRP	HE609081	TGGGCAGTCAGTGGATGTAA	TGGGATAAAGTAGGGGCTCA
*Me1*_SOCS	HE609098	AGCATACAAAGACGCAAGGA	TCACACTCACCGAAAATAAGAAG
*Me2-*IL 17	HE609102	TCCAACACATCCTTTCCAGA	AACACACAATACACACACTCACAATC
*Me3*-MIF	HE609105	CCATTGAGACCTACATTTCCTC	TATGCCGACTTTTGCCATTT
Mytimycin	HE610032	TCGTTGTGTTGTCTGTTATGTCC	CGCTTTTGTTTGTTGTTTGTT

Tissue mRNA expression was investigated using quantitative real-time PCR (qRT-PCR) with SYBR Green (Power SYBR® Green PCR Master Mix, Applied Biosystems, USA) on a 7900HT Fast Real-Time PCR System (Applied Biosystems, USA). PCR cycling conditions were 50°C for 2 min, 95°C for 10 min, followed by 40 cycles of 95°C for 15 s, 60°C for 1 min. Samples were run in triplicates. For each primer a melting curve of the PCR product was also performed to ensure the absence of artifacts. The comparative CT method (delta Ct) for the relative quantification of the respective transcript expression was used as described by Livak and Schmittgen (2001). Expression values of putative immune-related contigs were normalized using the 18S rRNA as reference gene. We tested 18S and 28S rRNA as putative reference genes and normfinder (http://www.mdl.dk/publicationsnormfinder.htm) selected 18S rRNA as most suitable reference gene. Data were transferred to GraphPad Prism (GraphPad Software Inc., USA) and plotted as histograms of normalized expression of target genes.

## Results and Discussion

### Transcriptome overview

**Figure 1:**
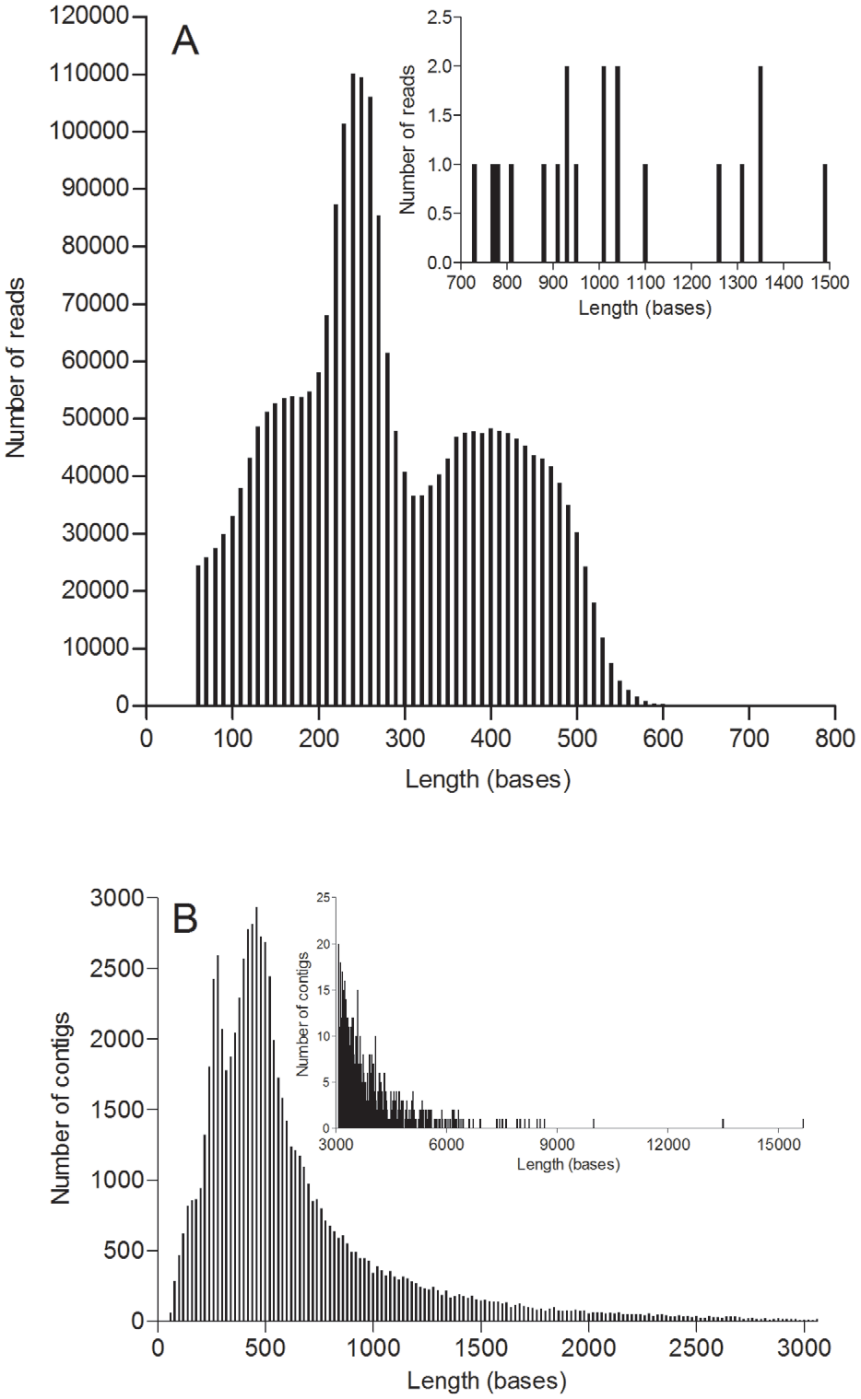
Overview of the *Mytilus edulis* sequences and assembly. A) size distribution of 454 read sizes sequenced with FLX and Titanium chemistry after removal of adaptor and SMART sequences as well as PolyA tails (insert: reads of 700–1500 bp length). B) size frequency of assembled contigs (insert: contigs >3000 bp length). For dataset details see table S1.

In order to create a comprehensive transcriptome data set, sequences from different tissues and stress- and immune-challenged *M. edulis* individuals were generated on a Genome Sequencer FLX system (454 Life Sciences, Branford, CT, USA). A total of 2393441 reads, with an average length of 279 bp, passed the quality control and filtering and entered the assembly pipeline (Figure 1A). From these, 2216965 reads could be assembled into 74622 contigs with an average length of 645 bp (Figure 1B) and an n50 value of 767 bp. 176476 reads could not be assembled and remained as singletons. The GC-Content of the transcriptome was 34.45% and the AT/GC-ratio 1.90. Mean coverage of contigs (number of bases in all reads within the contig/number of bases of the contig consensus sequence) was 11.81x with a minimum of 1.04 and maximum of 17989x. Coverage per base of the whole transcriptome was mean 12.66x and max. 30964x. The transcriptome project is deposited as SRA study ERP00937 and Bioproject 75259 at the European Nucleotide Archive (ENA). Assembled transcriptome sequences are deposited in the ENA as Transcriptome Shotgun Assembly (TSA) and accession numbers are given throughout the text or tables.

### Comparison with known Mytilus sequences

**Figure 2:**
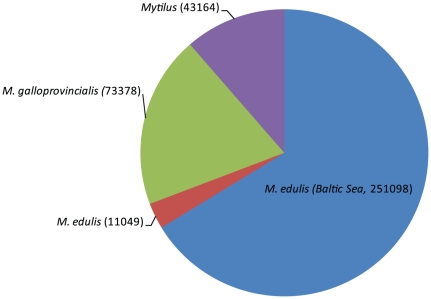
Overview of current *Mytilus* nucleotide sequences. Number of sequences of *M. edulis* and *M. galloprovincials* from Craft et al [18] generated by pyrosequencing (454 Roche, Maryland, USA) combined with sequences of *M. edulis*, *M. galloprovincials* downloaded from NCBI (December 2010), as well as all other sequences of the *Mytilus* genus downloaded from NCBI (December 2010) were compared to the number of Baltic Sea *Mytilus edulis* contigs and singletons generated in the present study. Respective number of sequences is given in brackets. For details of sequence downloads see material and method section.

To benchmark the generated transcriptome, contigs and singletons were compared with previously published sequences of *M. edulis* and *M. galloprovincialis*. Specifically, we obtained *M. edulis*, *M. galloprovincialis* sequence data from a recent pyrosequencing project by Craft *et al* [18] and downloaded *M. edulis* and *M. galloprovincialis* sequences from the NCBI database (http://www.ncbi.nlm.nih.gov, downloaded 12/2010) as well as retrieved all sequences belonging to the *Mytilus* genus from the NCBI database (http://www.ncbi.nlm.nih.gov, downloaded 12/2010). The number of contigs and singletons of the current *M. edulis* database more than doubled the current public sequence information (Figure 2). We compared the new generated *M. edulis* sequences with previously published sequences on the nucleotide level using BLASTn and cut off e-values of 1e ^−^10 to 1e ^−^100, to investigate whether the new database increased also the amount of new sequence information. Most of the *M. edulis* (78–90%) and *M. galloprovincialis* (64–95%) newly assembled contigs from sequences generated by Craft et al. [18], or sequences deduced from NCBI, showed high similarity to the *M. edulis* sequences of the present study (Figure S1 A, B). Also the comparison with all *Mytilus* genus sequences (from NCBI) showed a high identification rate of 65%. Singletons from the Craft *et al.* [18] pyrosequencing project, i.e. sequences which could not be assembled into contigs, were only identified to 30–40% (Figure S1 A, B), which could result from the lower quality of the sequences (personal observation). Of the present *M. edulis* database, 42392 (57%) of the 74622 contigs and 104819 (60%) of the 176476 singletons did not show any similarity to previously published *Mytilus* sequences and thus represent new sequence information (Figure S1 D).

### Transcriptome annotation

**Figure 3:**
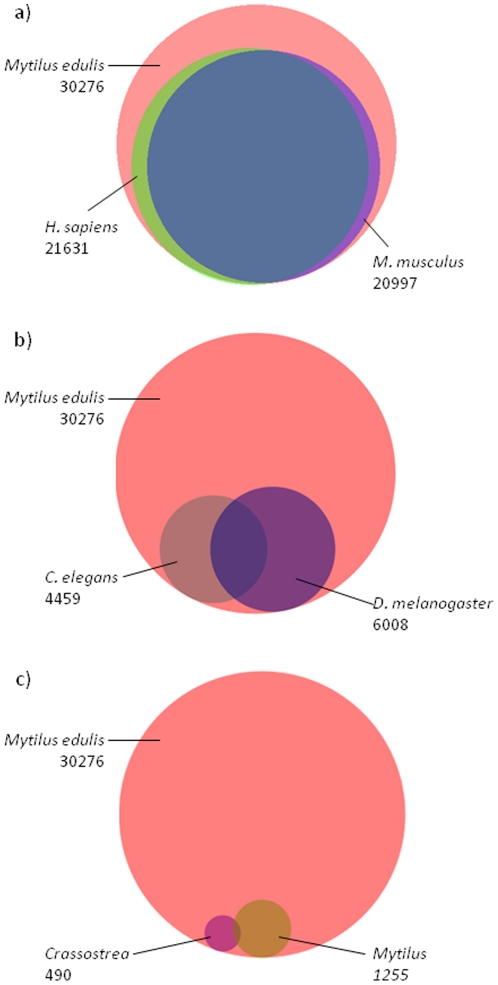
Venn diagrams of all annotated *M. edulis* contigs by genes of (a) *H. sapiens* and *M. musculus*, (b) *C. elegans* and *D. melanogaster* and (c) *Mytilus spp.* and *Crassostrea spp.* (cut off e-value of e≤10^−3^). Venn diagrams were calculated using Biovenn http://www.cmbi.ru.nl/cdd/biovenn/index.php [118].

To assign a putative function to the assembled contigs and search for immune related genes, *M. edulis* contigs and singletons were compared against public databases (UniprotKB/Swissprot, nr/nt NCBI) using BLAST. Over 60% of contigs with sequence sizes >500 bp could be annotated either with BLAST matches, GO terms or proteins domains. Singletons of the same length were only annotated up to 27% (Table 2). A more detailed analysis of the matching BLAST hits showed that most BLAST annotations were derived from *H. sapiens* or *M. musculus* sequence records and only a minor percentage were annotated by *C. elegans* or *D. melanogaster* derived genes (cut off e-value of e≤10^−3^; Figure 3 a, b). An even lower amount of sequences were similar to public available annotated sequences of *Mytilus* or *Crassostrea* (Figure 3 c). The minor percentage of contigs annotated by *Mytilus* or *Crassostrea* genes compared to the classical model organisms *H. sapiens*, *M. musculus*, *D. melanogaster* and *C. elegans*, most likely results from the fact that a higher number of annotated genes are public available for the classical model organisms. Interestingly there is only a minor overlap (<1%) between transcripts annotated by *Mytilus* and *Crassostrea* sequence records and at the same time also annotated by *H. sapiens*, *M. musculus*, *D. melanogaster* and *C. elegans*. This indicates that in most cases bivalve-specific genes (e.g. antimicrobial peptides) were annotated by public available bivalve sequence records. In contrast a nearly complete overlap was observed between *M. edulis* transcripts which were highly similar to annotated genes of *H. sapiens*, *M. musculus*, *D. melanogaster* and *C. elegans*. In total however, >4 times more contigs were annotated by *H. sapiens* or *M. musculus* compared to *D. melanogaster* or *C. elegans*. In these classical model organisms, the number of coding-genes ranges from 20–25000 in *H. sapiens* and *M. musculus* to 13–20000 in *D. melanogaster* and 20000 in *C. elegans* [30]. Thus the only slightly lower amount of coding sequences in *C. elegans* and *D. melanogaster* compared to the two vertebrates cannot explain the much lower observed annotation rate of *M. edulis* contigs by the two ecdysozoan invertebrates.

**Table 2:** Summary of the assembly and annotation results in the *Mytilus edulis transcriptome*.

	all sequences	100–500 bp	>500 bp
total number of contigs	74622	38742	35096
contigs with BLAST matches	30276	10551	19676
contigs with assigned GO terms	16415	5183	11227
contigs with InterProScan matches	22967	7321	15640
contigs without matches	41985	27152	14088
% annotated	44	30	60
Total number of singletons	176476	150843	7523
singletons with blast matches	33177	30586	2030
singletons without matches	143135	120109	5478
% annotated	19	20	27

**Figure 4:**
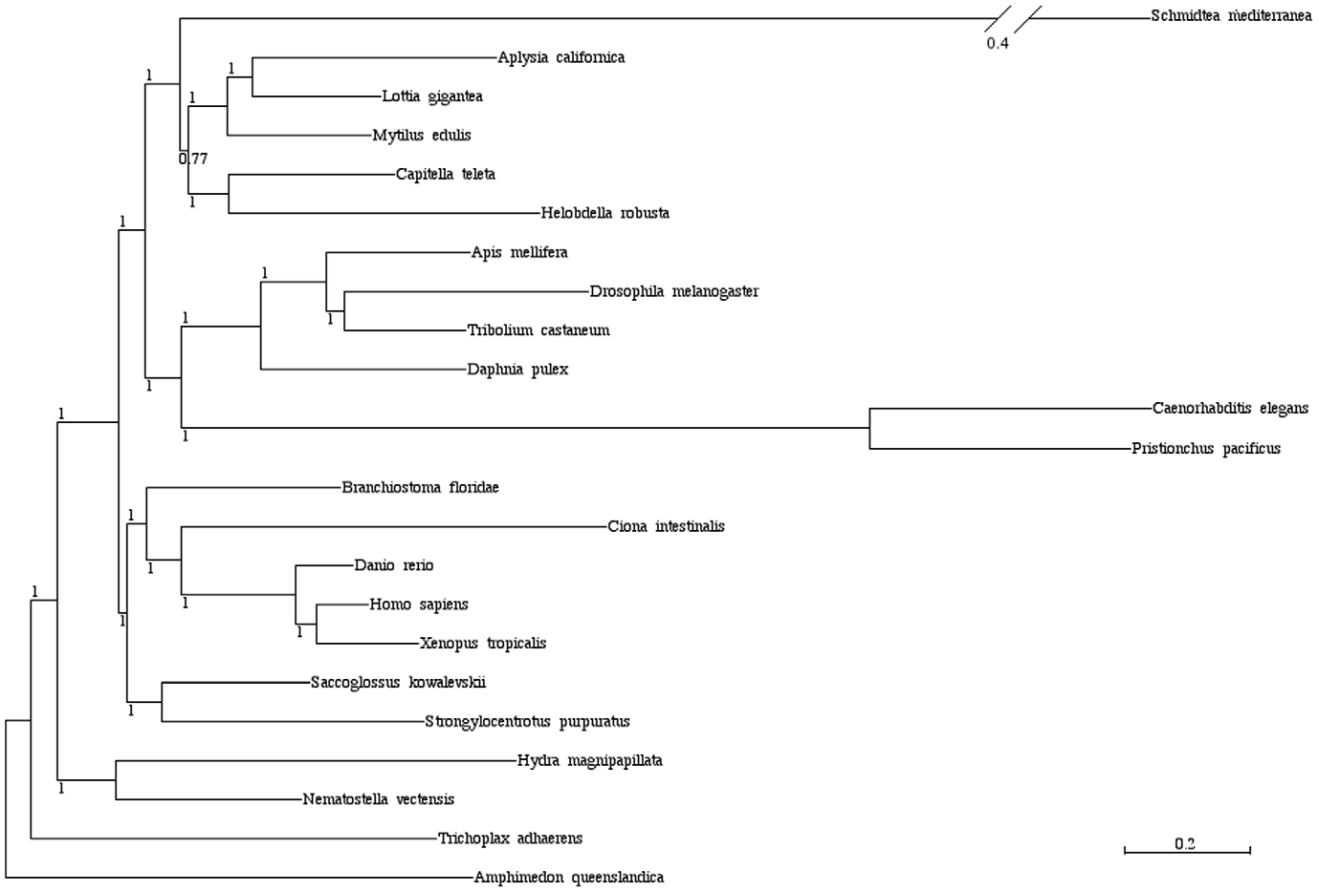
Phylogeny of 24 animal species based on EST sequences of *Mytilus edulis* and predicted genes from completed genomes. (71 genes, 30,784 unambiguously aligned positions, 0% missing data) reconstructed using the CATGTR +gamma model under a Bayesian analysis. Major accepted metazoan clades (e.g. Lophotrochozoa, Ecdysozoa, Protostomia) are supported. Level of bootstrap support is indicated. The scale bar indicates the number of changes per site.

Phylogenetic analysis of 71 gene groups and 23 species ranging from *Amphimedon* to *Homo sapiens* further demonstrate the basal position of lophotrochozoans to which *M. edulis* belongs within protostomes (Figure 4). This might explain the significant better recovery of shared genes with deuterostomes, rather than with the ecdysozoans *C. elegans* and *D. melanogaster*, which are extremely fast evolving and have suffered from major gene loss [13].

### GO- and Protein domain analysis

**Figure 5:**
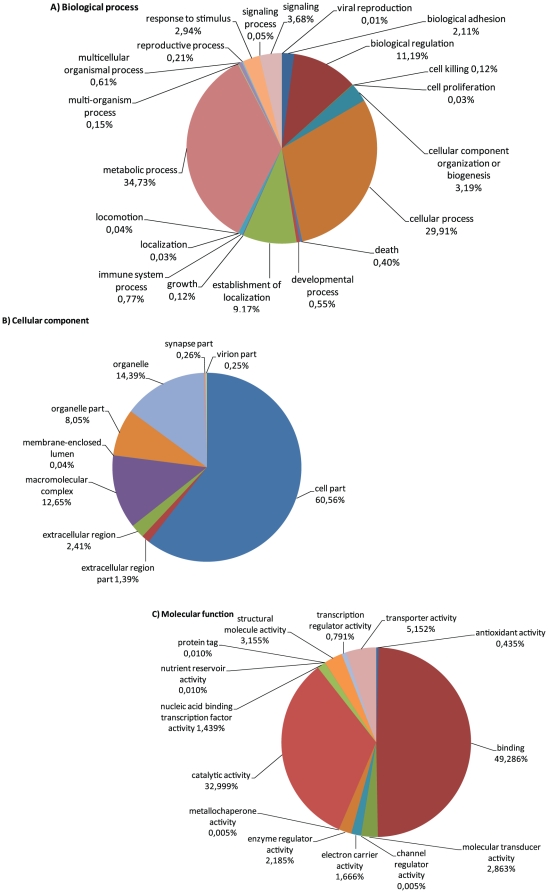
Gene Ontology of annotated contigs from *M. edulis*. Distribution in percent (%) of gene ontology terms among the annotated sequences. Gene Ontologies are represented as general function categories (A) biological process, (B) cellular component and (C) molecular function.

**Figure 6:**
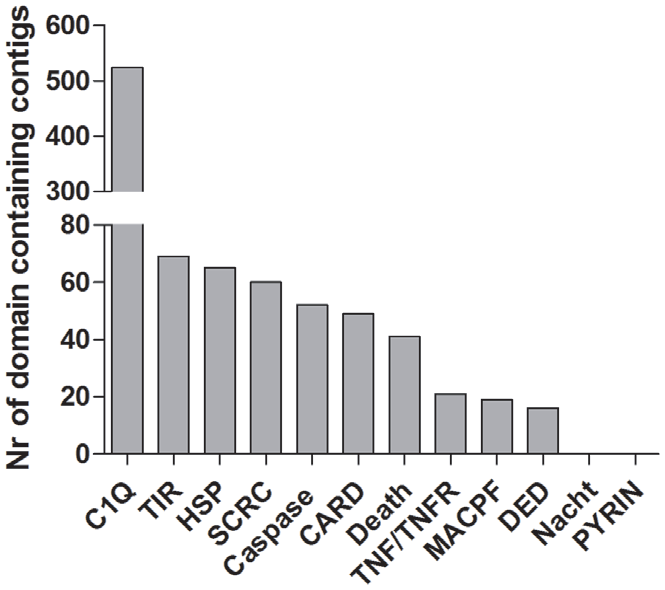
Immune relevant protein domains in the *Mytilus edulis* transcriptome: number of contigs which show immune system relevant proteins domains assigned by InterProScan.

In a second step, contigs were interrogated for predicted protein domains using InterProScan and sorted into Gene Ontology (GO) terms deduced from the InterProScan annotation. This resulted in an assignment of protein domains to 22967 contigs and functional GO-terms to 16415 contigs (Table 2). As reported in other mollusc transcriptome studies [31], [32] the largest proportion of GO assigned sequences fell into 2–3 broad categories of the general term categories ‘biological process’, ‘cellular component’ and ‘molecular function’ (Figure 5). We were especially interested in immune related sequences but within the GO-category ‘biological process’, only a small amount of transcripts (127 transcripts) fell into the category immune system process. These contigs mainly consisted of predicted toll-like receptor – like transcripts. However, by using protein domain prediction (InterProScan), a high number of predicted immune-related protein domains e.g. 524 C1q domain containing contigs could be identified in the transcriptome (Figure 6). Within the classical complement pathway (see below) C1q acts as the target recognition protein but is also considered to bind directly to pathogens and enhance phagocytosis and was further found to function as a lectin and activate the lectin complement pathway [6], [33]. First expression analysis in *Mytilus galloprovincialis*, *Chlamys farreri* and *Ruditapes decussatus* found C1q-domain containing transcripts to be up-regulated upon stimulation with LPS, different bacteria and parasites and appear to play an important role in host defense [6], [34], [35]. Previous studies identified high numbers of C1q-domain containing genes in vertebrates such as in the human (31 models, [36]) and zebrafish genome (52 models, [37]), and only lower numbers were found in invertebrates like the sea urchin *S. purpuratus* (4 gene models, [8]) or the urochordate *Ciona intestinalis* (2 gene models, [38]). This led to the assumption of a higher diversification of this gene family in the vertebrate lineage. Recent investigation of genomes and transcriptomes of molluscs and other invertebrate species however revealed a high number of C1q-containing gene models [39]. Gerdol et al [39] reported at least 168 different C1q domain-containing transcripts in *M. galloprovincialis* transcriptome and in the present *Mytilus edulis* transcriptome 524 contigs exhibiting a C1q domain were found. Our finding support the hypothesis of the expansion of this gene family in *Mytilus* while this seems not to be the general case in other bivalves [39]. Other highly abundant protein domains comprise the TIR, SRCR, Caspase, CARD and DEATH domain, which are found in a large number of receptor and adaptor molecules involved in immunity and apoptosis. Interestingly the NACHT domain, a key element of NOD-like receptors (see below), as well as the PYRIN domain was absent which indicated a possible absence of the NLR pathway.

To identify immune related genes we therefore investigated the *Mytilus edulis* database in a combined approach by key-word and protein domain search. Contigs identified by key word search within their BLAST annotation were manually verified (i.e. inspected for the expected protein domains for the specific gene) and vice versa: characteristic protein domains and domain combinations were used to identify contigs with putative immune function which were then subsequently verified by their BLAST annotation.

### Identification of immune- related transcripts in Mytilus edulis 1. Pathogen sensors: TLRs, NLRs, RLRs, GNBPs and PGRPs

A first focus of the present study was the identification of pathogen sensors in *Mytilus edulis*, as the first line of defense starts outside the cell, via the recognition of pathogens to prevent an infection. Toll like receptors (TLRs), peptidoglycan recognition receptors (PGRPs) and gram-negative binding proteins (GNBPs) are pathogen recognition receptors (PRRs) which recognize bacteria, fungi and viruses on the cellular surface. NOD-like receptors (NLRs) and RIG-like receptors (RLRs) play a major role in the recognition of intra-cellular bacteria and viruses, respectively.

#### 1.1 Toll receptor pathway

**Figure 7:**
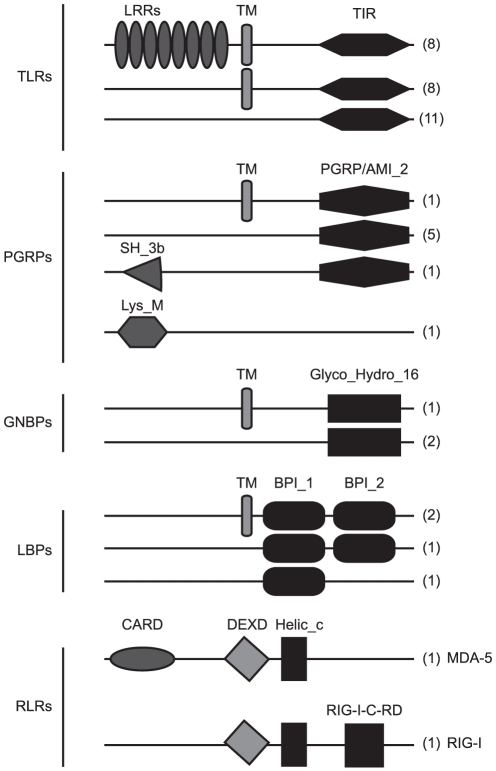
Pattern recognition receptors (PRRS) in *Mytilus edulis*. Schematic overview of protein structures and number of contigs (in brackets) of identified PRRS in the *M. edulis* transcriptome. Protein domains are: Leucine-rich-repeat (LRR), transmembrane (TM), TLR and IL-1 receptor (TIR), Peptidoglycan recognition protein/amidase 2 (PGRP/ami_2), Glycosyl hydrolase 16 (Glyco_Hydro_16), Bactericidal permeability-increasing protein (BPI)/Lipopolysaccharide-binding protein (LBP)/Cholesteryl ester transfer protein (CETP) N-terminal (BPI1) and C-terminal domain (BPI2), caspase recruitment domain (CARD), DEAD and DEAH box helicases (DEXD), Helicase_C (Helic_c) and the C-teminal regulatory domain of RIG-I (RIG-I_c_RD). Contig length was set generic, proteins domains were deduced from SMART or NCBI Dart using default thresholds.

The TLR pathway is a highly conserved pathway and TLRs are found throughout the animal kingdom from basal metazoans to vertebrates [40]. TLRs recognize microbial structures via the extracellular leucine-rich-repeat (LRR) domain; signal transduction takes place by the intracellular toll-interleukin-domain (TIR) domain and TIR domain containing adaptor molecules. Previous investigation of bivalve TLRs only identified a single TLR in *Mya arenaria*, *Chlamys farreri* and *Crassostrea gigas*, respectively [41], [42], [43] or 2 transcripts in *Mytilus galloprovincialis* [7] which led to the assumption of a small TLR repertoire being present in bivalves. The transcriptome investigation of *M. edulis*, however, conveys a different picture with 27 putative TLR encoding contigs (e-value≤10^−10^, Table 3, Figure 7). Eight of these contigs show the typical domain structure of TLRs (LRR-TM-TIR), another 8 are comprised of a TIR and transmembrane domain and 11 transcript fragments only show a TIR domain but are highly similar to TLRs of other organisms (Table 3). In the purple sea urchin *S. purpuratus*, the high diversification of TLRs (222 TLR gene models [8]) was assumed to be a result of the absence of the adaptive immune system and the development of a complex and large body size, benthic lifestyle and long life span [40]. This might also be valid for bivalves and may explain the relatively high number of TLR transcripts found in the present transcriptome. Bivalves like the giant clam *Tridacna gigas* reach extremely large sizes, several bivalves e.g. the ocean Quahog *Arctica islandica* are extraordinarily long lived [44] and due to their filter feeding mode of life bivalves are in constant contact to the surrounding microbial community. A high number of bivalves and other molluscs also harbor endosymbiontic bacteria [45], [46] which have to be recognized and controlled for optimal symbiosis. TLR diversification might play a pivotal role in such a control of microbial communities. However, in *D. melanogaster* not all TLRs are solely involved in the immune response but are also players in developmental processes [40]. The verification of transcript models and the functional analysis of the identified TLRs in *Mytilus edulis* have to be undertaken as a next step to understand the importance of TLRs in the bivalves' immune system.

**Table 3:** Toll-like receptor like contigs identified in *Mytilus edulis* by blast annotation and domain search.

Contig information					Best blast hit (UniprotKB/Swissprot)					
*M. edulis* Accession	Domains identified	Contig length (bp)	Nr. of reads	longest ORF (aa)	Accession	Protein names	Organism	Length	Identity	E-value
HE609229	TIR	533	3	118	P08953TOLL	Protein toll	*Drosophila melanogaster*	1097	37.0%	4.0×10−18
HE609235	TIR	505	4	136	B2LT62TLR2	TLR 2	*Capra ibex*	784	30.0%	4.0×10−11
HE609240	TIR	529	4	117	Q6R5N8TLR13	TLR 13	*Mus musculus*	991	34.0%	2.0×10−15
HE609241	TIR	285	2	95	Q2V897TLR2	TLR 2	*Boselaphus tragocamelus*	784	40.0%	1.0×10−10
HE609217	TIR	915	15	294	B3Y618TLR2	TLR 2	*Macaca mulatta*	784	40.0%	5.0×10−14
HE609228	TIR	488	4	162	Q9QUK6TLR4	TLR 4	*Mus musculus*	835	29.0%	1.0×10−11
HE609239	TIR	280	2	79	Q9QUN7TLR2	TLR 2	*Mus musculus*	784	40.0%	4.0×10−13
HE609230	TIR	549	3	120	Q9EPW9TLR6	TLR 6	*Mus musculus*	795	42.0%	8.0×10−13
HE609223	TIR	854	8	165	P08953TOLL	Protein toll	*Drosophila melanogaster*	1097	27.0%	7.0×10−10
HE609231	TIR	787	5	108	Q9QUN7TLR2	TLR 2	*Mus musculus*	784	39.0%	3.0×10−12
HE609222	TIR	254	3	77	Q6T752TLR2_	TLR 2	*Equus caballus*	784	49.0%	1.0×10−14
HE609224	TM,TIR	557	6	172	Q6R5N8TLR13	TLR 13	*Mus musculus*	991	26.0%	9.0×10−10
HE609234	TM,TIR	524	3	174	Q9EPW9TLR6	TLR 6	*Mus musculus*	795	28.0%	9.0×10−16
HE609219	TM,TIR	645	5	214	Q9QUN7TLR2	TLR 2	*Mus musculus*	784	25.0%	2.0×10−11
HE609232	TM,TIR	1190	11	238	P58727TLR4	TLR 4	*Felis catus*	833	36.0%	9.0×10−26
HE609233	TM,TIR	724	7	154	Q15399TLR1	TLR 1	*Homo sapiens*	786	36.0%	6.0×10−24
HE609218	TM,TIR	1857	17	553	Q9DGB6TLR22	TLR 2 type-2	*Gallus gallus*	781	23.0%	2.0×10−23
HE609237	TM,TIR	827	12	237	Q9DGB6TLR22	TLR 2 type-2	*Gallus gallus*	781	30.0%	8.0×10−23
HE609236	TM,TIR,	1149	7	332	Q9DD78TLR21	TLR 2 type-1	*Gallus gallus*	793	28.0%	7.0×10−24
HE609238	5xLRR, TM, TIR,	1687	20	544	Q6R5N8TLR13	TLR 13	*Mus musculus*	991	26.0%	1.0×10−31
HE609225	11xLRR, LRRNT, 4x LRR, TM,TIR	3744	125	1138	P08953TOLL	Protein toll	*Drosophila melanogaster*	1097	26.0%	3.0×10−64
HE609226	2x LRR, LRRCT,TM, TIR	1260	10	419	Q15399TLR1	TLR 1	*Homo sapiens*	786	33.0%	3.0×10−25
HE609216	2x LRR,LRRCT,TM,TIR	1645	18	540	Q9QUK6TLR4	TLR 4	*Mus musculus*	835	24.0%	1.0×10−18
HE609227	LRRCT, TM, TIR	1000	10	333	Q689D1TLR2	TLR 2	*Canis familiaris*	785	28.0%	4.0×10−29
HE609242	LRRCT,LRRNT,LRRCT,TM,TIR	2477	186	479	P08953TOLL	Protein toll	*Drosophila melanogaster*	1097	23.0%	1.0×10−20
HE609220	LRRCT,TM,TIR	775	11	225	Q9EPW9TLR6	TLR 6	*Mus musculus*	795	27.0%	3.0×10−14
HE609221	LRRCT,TM,TIR	1023	15	283	Q704V6TLR6	TLR 6	*Bos taurus*	793	24.0%	2.0×10−22

Only contigs with showed a TLR blast annotation with e-values≤10^−10^ and at least a TIR domain are shown.

**Figure 8:**
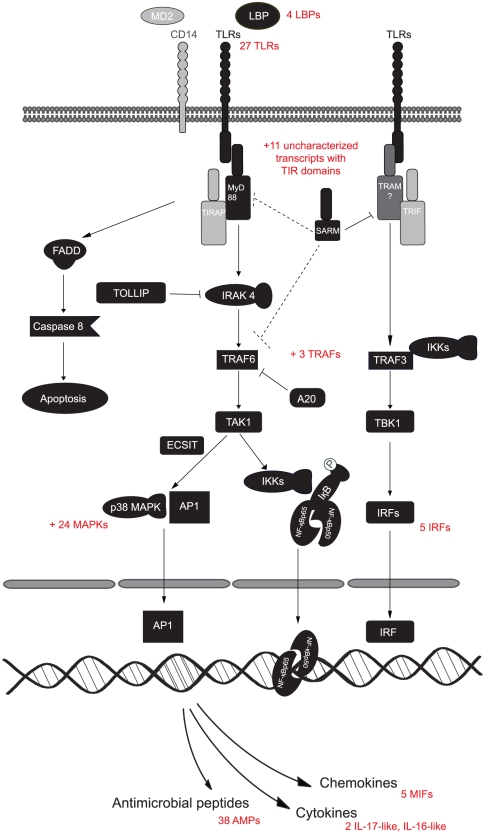
The TLR pathway in *Mytilus edulis*. Schematic comparison of *Mytilus* and vertebrate TLR pathway members. Homologous contigs of *Mytilus* are marked in black and vertebrate genes marked in grey. The broken arrows from SARM display possible interactions with Myd88 or TRAF6 deduced from other studies (see text) in the absence of TRAM.

To investigate the composition of the downstream TLR pathway, we searched for other molecular constituents of the TLR pathway, e.g. key adaptor molecules like the myeloid differentiation primary response protein 88 (MyD88), TIR domain-containing adapter molecule (TIRAP/MAL), TIR domain-containing adapter molecule 2 (TRAM/TICAM2) and the TIR domain-containing adapter molecule 1 (TRIF). The use of the different adaptor molecules differentiate the TLR signaling network in a MYD88 -dependent (MYD88, TIRAP) and -independent (TRAM, TRIF) pathway. In cnidaria main players of the MyD88 -dependent and -independent pathway could be identified such as MYD88, TRIF and TRAM (TICAM2) [10]. However, in the lower deuterostomes and protostomes like *S. purpuratus*, *D. melanogaster* and *C. elegans*, TLR signaling seems to takes place solely via the MyD88 dependent pathway [8], [11]. TIRAP (MAL) is also not present in these species leaving MyD88 as the single primary adaptor molecule for downstream signaling. Most constituents of the MyD88 dependent TLR pathway are found in *M. edulis*, including MyD88 (*#*HE609071), IRAK 4 (*#*HE609065), TRAF6 (*#*HE609075) and others (Figure 8, Table S4). We did not find clear evidence for the MYD88- independent pathway, i.e. distinct orthologues of TRAM and TRIF, which is in line with other findings in *Mytilus galloprovincialis* [7] and the so far hypothesized absence of this pathway in lophotrochozoan invertebrates. One weak hit for TRAM (#HE609076) is however present in *Mytilus edulis* as well as putative transcripts for further components of the Myd88 independent pathway such as TRAF3 (*#*HE609056), TBK1 (*#*HE609091) and several interferon regulatory factors (IRF, *#*HE609042, HE609043, HE609044, HE609045, HE609046). In *S. purpuratus* and *B. floridae* a high number of yet uncharacterized TIR-domain containing molecules were hypothesized to functionally replace TIRAP, TRAM or TRIF [8]. This might also be the case for *M. edulis* where 11 uncharacterized transcripts with TIR domains could be identified. Another TIR domain containing molecule involved in the MyD88 independent TLR pathway in humans, and present in *M. edulis*, is SARM (Sterile alpha and TIR motif-containing protein, *#*HE609073). The exact function of SARM is still under debate as a variety of functions (immune system, neuronal development) and interaction partners where found within the deuterostome and protostome lineages [47]. In humans and also in the horseshoe crab *Limulus polyphemus* SARM is a negative regulator of TRIF in the Myd88 independent line of the TLR pathway and does not interact with Myd88 [48]. In a recent investigation of the *B. floridae* SARM ortholog however, a convincing interaction of SARM with Myd88 and TRAF6 within the Myd88 dependent pathway of the TLR pathway was reported. The investigation such an interaction between SARM and MyD88 in *M. edulis* could thus give a further indication whether the MyD88 independent pathway is present or absent in *M. edulis*. In *C. elegans* however, TIR-1, the *C. elegans* ortholog of SARM, does not at all react with the sole TLR in this species, but utilizes a p38 MAPK-signaling pathway [48]. It remains to be investigated whether a MyD88 independent pathway exists also in protostomian and deuterostomian invertebrates.

#### 1.2 Rig-like receptors (RLRs)

Intracellular sensors of foreign RNA are the retinoic acid inducible gene I (RIG-I) and the melanoma differentiation associated gene-5 (MDA-5) [49]. Signal transduction takes place via the N-terminal CARD domain. RIG-I and MDA-5 like genes have been identified throughout the animal kingdom from cnidaria up to vertebrates and also in the *Mytilus edulis* transcriptome gene fragments are found (Figure 7, Table S4). The MDA-5 (*#*HE610043) orthologue in *M. edulis* is composed of the classical protein domain structure with an N-terminal CARD domain, followed by a DEAD/DEAH box helicase domain and a C-terminal helicase-c domain. The identified RIG-1 fragment so far lacks the N-terminal CARD domain which might however be revealed by full-length sequence investigation. However, in *S. purpuratus* RIG-I like genes were found to contain either a CARD or a DEAD domain. In *D. melanogaster* Dicer2 is hypothesized to be the functional equivalent to RLRs and lacks a CARD and Helicase c domain [50]. In contrast dicer related helicases (DHRs) in *C. elegans* contain at least a helicase c domain. The respective genes of both species align very well with *M. edulis* putative RIG-I and MDA-5 (Table S4). Thus it remains to be elucidated which effector domain is present in *M. edulis* RIG-I. Although a number of key components of the RLR pathway were found in the *M. edulis* transcriptome (Table S4), e.g. ATG12 (*#*HE609120) and ATG5 (*#*HE609116), the main adaptor protein IPS-1/MAVS/Cardif/Vista (IMCV) could not be identified. So far IMCV homologues were only found from vertebrates down to the purple sea urchin [8] but are not reported for any of the lower invertebrates or within the protostome lineage. It is important to note that the identified key components of the RLR pathway in *Mytilus edulis* are also involved in other pathways such as autophagy and not restricted to the RLR pathway. Thus the question remains if the identified RIG-1 and MDA-5 like genes work in the same manner as reported for mammals i.e. initiate interferon production and antiviral immunity, or rather initiates specific, RNAi-mediated antiviral effector mechanisms as found in plants, fungi and invertebrates by producing virus-derived small interfering RNAs (siRNAs) [51].

#### 1.3 NOD-like receptors (NLRs)

While TLRs and RLRs seem to be well conserved throughout the animal kingdom, no NLRs were reported so far for the protostome group such as *D. melanogaste*r and *C. elegans* or other organisms from the ecdysozoan or lophotrochozoan lineage [52]. However the occurrence of NLRs in cnidarians [14] led to the hypothesis that NLRs were secondarily lost in the protostome lineage. NLRs contain a nucleotide binding NACHT domain in combination with an C-terminal LRR domain and N-terminal effector binding domain (e.g. CARD, PYD, BIR) [53]. Especially in lower deuterostomian invertebrates and vertebrates as well as in early diverging metazoans a high number of NLR genes were found, emphasizing the importance of these receptors in the innate immune system [14]. We speculated that the low number of sequences available for lophotrochozoans could have obscured the identification of NLRs so far and a larger transcriptome analysis might reveal NLRs also in bivalves. However in the present *Mytilus edulis* transcriptome the search for NLRs and NACHT domains was not successful. Moreover key constituents of the NLR pathway are lacking in *M. edulis*, such as RIP2 (receptor-interacting protein kinase 2) and ASC (speck-like protein containing a CARD), which, like MyD88 or TRAM in the TLR pathway, act as adaptor molecules for different NLRs [53], as well as Caspase 1, a key molecule activated by inflammasome-forming NLRs [54]. It is interesting to speculate that also in *Mytilus* and bivalves other intracellular PRRs may have taken over the intracellular recognition of bacterial motifs and other danger signals.

#### 1.4 PGRPs/GNBPs/LBPs

Peptidoglycan recognition proteins (PGRPs), glucan binding proteins (GNBPs, formerly named Gram-negative binding proteins [55]) and lipopolysaccharide-β-1,3-glucan binding proteins (LBPS) comprise different families of intra- and extracellular proteins with pleiotropic functions from recognition of PAMPs (pathogen associated molecular patterns), enzymatic cleavage to antimicrobial actions. While PGRPs are conserved from insects to mammals [56] - with the exception of nematodes-, GNBPs are absent in mammals [57] and LBPs in insects, but both can be found in molluscs.

**Figure 9:**
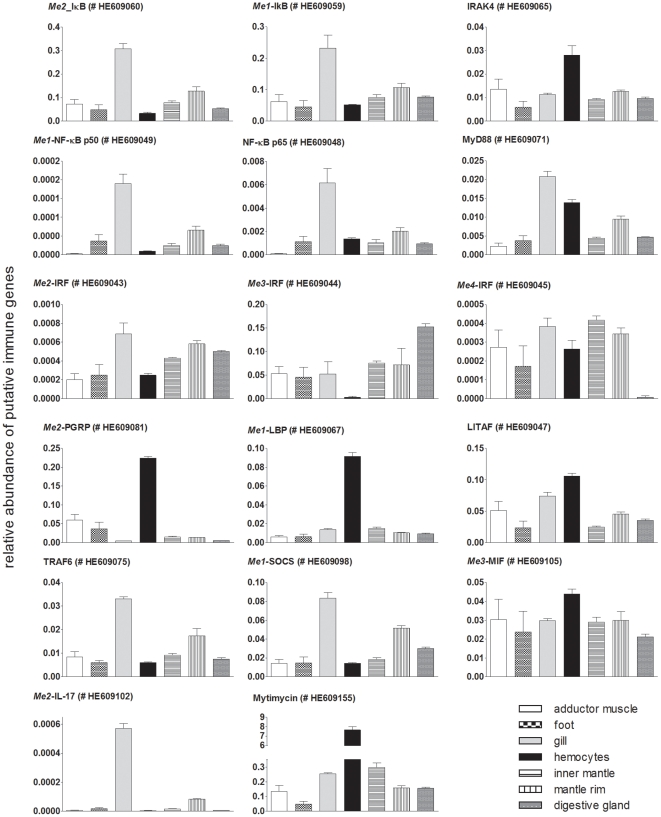
Quantitative expression of putative immune gene encoding contigs determined by qRT-PCR in different tissues of *Mytilus edulis*. Gene expression was measured in tissue specific cDNA pools consisting of 6 individuals each and is displayed as relative expression using the 18S rRNA as the control transcript. Results are mean ± SEM (n = 3 technical replicates). ENA accession numbers are given in brackets.

In *M. edulis* seven different transcript fragments encoding putative peptidoglycan recognition proteins were found which exhibit the conserved PGRP/amidase2 domain (Figure 7, Table S4). Mammals possess only 4 PRGPs which are secreted and are directly bactericidal or have an amidase function to hydrolyze peptidoglycan (PGN). PGN recognition in mammals is mediated extracellular by TLRs and intracellular by NLRs [56]. In insects a high variety of shorter and longer PGRPs as well as transmembrane or secreted transcripts are found, of which some are also bactericidal and have amidase function but, more importantly, can initiate signal transduction via the TOLL or IMD pathway or activate the prophenoloxidase cascade [58]. In *Drosophila* PGRPs are moreover quite specific and the different PGRPs were found to recognize different forms of peptidoglycan and thereby discriminate between gram positive and gram negative bacteria. Of the seven *M. edulis* transcripts, one represents the first bivalve PGRP with a transmembrane domain (*#*HE609080), thus, as found in *Drosophila*, bivalves seem to possess soluble and membrane bound forms of PGRPs. Further, one contig contained an additional SH3b domain (*#*HE609081), which are mainly known from bacteria and found only in a few eukaryotes. So far SH3b domains have not been reported in combination with a PGRP/Amidase2 domain (UniprotKB). Sanger sequencing verified the identified transcript of *M. edulis* and high levels were found in gill tissue of *M. edulis* compared to other tissues and hemocytes (Figure 9). Numerous PGRP orthologues were reported in other bivalves such as scallops and oysters [59], [60], [61], [62] and gene expression could be stimulated by bacterial infection or peptidoglycan (PGN) challenge, whereas LPS stimulation did not result in a significant up-regulation [59], [60], [62]. It had thus been concluded that PGRPs might indeed be important for the recognition and elimination of pathogens, presumably in particular gram-positive bacteria.

Glucan binding proteins (GNBPs) were studied extensively in arthropods [55], [63] and found to recognize β-1,3-glucans of fungal cell walls, LPS, and in cooperation with PGRPs, gram positive bacteria, but not PGN. In arthropods GNBPs are involved in the prophenoloxidase (PO) system, TLR pathway as well as the modulation of hemocytes functions such as phagocytosis, adhesion and degranulation. Research on GNBP function in bivalves is still scarce. Only two studies were conducted so far in the bivalves *Perna viridis* and *C. gigas* [64], [65] in which GNBPs were reported to be involved in the PO system. In *M. edulis* three different GNBPs with a glyco-hydrolase-16 domain were found (Figure 7, Table S4). As described above for PGRP, one GNBP additionally exhibited a transmembrane domain (*#*HE609089) which could hint towards membrane bound forms of GNBP. In contrast to insects, molluscs also exhibit lipopolysaccharide and β-1,3-glucan binding proteins (LBPs) [66], [67]. LBPs were studied in crustacean species, which lack PGRPs and activate the PO system via LBP [68]. The *Mytilus edulis* transcriptome contains 4 LBP transcript fragments with a characteristic BPI (bactericidal permeability-increasing protein/lipopolysaccharide-binding protein/cholesteryl ester transfer protein) 1 and/or 2 domain (*#*HE609067, HE609068, HE609069, HE609070). Two sequences again were additionally characterized by transmembrane domains (Figure 7, Table S4). As for PGRP one of these transmembrane containing LBP transcripts (*#*HE609067) was expressed highest in hemocytes (Figure 9).

In mammals extracellular sensing of gram negative bacteria is mediated by LBP, an adaptor for TLR4, whereas intracellular recognition is mainly accomplished by NLRs. *D. melanogaster*, which lacks LBP and NLRs, extracellular Gram positive and negative bacteria are recognized via PGRPs in cooperation with GNBPs which then activate the Toll or IMD pathway [55], [69], [70], [71], [72]. Intracellular recognition in *D. melanogaster* takes place via PGRP-Le which can activate apoptosis and act via the IMD and JAK/STAT pathway [73], [74]. In *C. elegans* the situation might be again completely different as this species lacks PGRPs, NLRs and GNBPs as well as functional TLRs or LBPs [69]. In *M. edulis* it remains to be investigated which of the LBPs, GNBPs and PGRPs recognize bacteria intra and/or extracellular and whether in *M. edulis*, as shown for *D. melanogaster*, PRGPs could functionally replace NLRs and act via the JAK/STAT pathway or the initiation of autophagy.

### 2. Transcription factors

#### 2.1 NF-κB

Many processes downstream of the PRRs are mediated by the transcription factor NF-κB, which is a key regulator of cellular processes such as cell proliferation, -survival and immunity. Members of the NF-*κ*B family have been identified in protostomes and deuterostomes and also in lower invertebrates like the sea anemone *Nematostella vectensis*, sweet water polyp *Hydra*, coral *Acropora millepora* (Cnidaria) and the demosponge *Amphimedon* [75]. In molluscs Rel family members were found in several bivalves and one gastropod species, and fragments contained the conserved RHD domain with the characteristic amino acid sequence of the DNA recognition loop and NLS [75], [76], [77], [78]. Tissue expression of the gene is ubiquitous in molluscs as is the case for other Rel genes in most taxa. Also in *M. edulis* two members of the Rel family with conserved RHD and NLS regions were identified with high similarity to the Rel family members p65 (*#*HE609048) and p50 (*#*HE609049, HE609050) (Table S4; Figure S2). Further 2 contigs highly similar to I*κ*B (*#*HE609059, HE609060) and one similar to IKK (*#*HE609061) were found in the *M. edulis* transcriptome. The putative NF-*κ*B and I*κ*B transcripts showed a similar expression pattern in the tissue panel, with highest expression in gill tissue (Figure 9).

#### 2.2 Interferon Regulatory Factor Family

A second conserved family of transcriptions factors that are important for the downstream regulation of the PRR mediated innate immune system signaling is the group of the interferon regulatory factor family (IRFs). IRFs are present from the most basal group of metazoans on and were found in porifera, placozoans, ctenophores and cnidarians. In vertebrates 10 members of the IRF family are known which function as major regulators of host defense [79]. IRF genes have two evolutionary conserved domains, the DNA-binding domain (DBD = IRF, Smart ID:SM00348) and the IRF association domain (IAD = IRF3, Pfam ID: PF10401), both already present in sponges. In cnidarians and bilaterians, IRF genes diversified into the IRF-1 (without IAD domain) and IRF4 (containing the IAD domain) branches. The IRF family expanded several times in distinct groups of bilaterians (molluscs, cephalochordates, tunicates and vertebrates), whereas they were lost or mutated beyond recognition in nematodes and insects. Although the function of the IRF family in vertebrates has been studied in considerable detail, very little is known about the function of invertebrate IRF genes. In molluscs 1–7 IRFs were found in different gastropod, bivalve and squid species [79]. All IRFs show the conserved N-terminal DBD domain and only in genome sequences of *Aplysia californica* and *Lottia gigantea* the combination of a DBD and IAP domain could be predicted [79]. In the *M. edulis* transcriptome 5 IRF members were identified which exhibit the conserved DBD domain and in one contig (*Me1-*IRF, *#HE609042*) the IAD domain as well (Table S4).This relatively high number of IRFs compared with previous findings in bivalves corroborates the conclusion of Nehyba et al [79] that the IRF family underwent an expansion in the taxon of molluscs; which was mainly deduced by the authors from the finding of 7 IRFs in the genome sequences of the gastropod *Aplysia californica* [79].

### 3. Cytokines, cytokine receptors and the JAK/STAT pathway

Classical sequence comparison using the major mammalian cytokines like interferon or interleukins did not yield a high number of identified cytokines in invertebrates [8], [11]. So far identification of invertebrate cytokine-like molecules like Spätzle in *D. melanogaster* or the Astakines in crustaceans showed no similarity to vertebrate cytokine structures (for review see, [80]). In the present study, we also could not identify any interferon (IFN) related sequences or IFN-domain containing contigs. However, 3 fragments putative encoding for interleukins were identified by blasting the *C. gigas* interleukin 17 mRNA sequence (EF190193.1) against the *M. edulis* transcriptome. *Me1*-IL-17 (*#HE609101*) and *Me2*-IL-17 (*#HE609102*) exhibit an IL17 protein domain and by tBLASTx analysis (UniProtKB) show similarity to interleukin 17 of *C. gigas* (for *#HE609101:* e-value of 1.00E-03, Table S4), *Oryzias latipes* (for *#HE609101:* e-value of 8.00E-04, for *#HE609102:* e-value 1.20E-02), *Rattus norvegicus* (for *#HE609102:* e-value 4.90E-01) and *H. sapiens* (for *#HE609101:* e-value of 1.6, Table S4). The putative transcript for IL16, *Me*-IL-16 (*#HE609100*), showed higher similarity to interleukin 16 of other organisms and exhibits an IL16 typical PDZ domain (Table S4). IL-17 seems to be quite well conserved compared to other cytokines as homologues were identified in the sea urchin genome, *C. gigas* ESTs (*Cg*IL-17) [81] as well as recently in the *B. floridae* genome [11]. Tissue expression analysis in the oyster revealed highest expression levels in gill tissue and bacterial injection or immersion lead to an increase in *Cg*IL-17 gene expression levels in the immunocytes [81]. Also in *M. edulis* the investigation of *Me2*-IL-17 (*#HE609102*) showed highest expression in gill tissue (Figure 9). The identification of a mollusc IL-17-receptor orthologue however, is still pending.

Another ancient and conserved cytokine of the immune system is the macrophage migration inhibitory factor (MIF), which is reported from cyanobacteria and sea urchins to humans [8], [82] and was also recently discovered in the group of mollusca [83], [84]. Previously found to be a T-cell cytokine of the adaptive immune system, MIF is emerging as an important mediator of the innate immune system and the conservation of MIF across taxa indicates an important biological function. In *M. edulis* 5 MIF-like transcripts with characteristic MIF domains were identified (Table S4). The ubiquitous distribution of the investigated *Me3*-MIF (*#HE609105*) in the different *Mytilus* tissues (Figure 9) is in line with the broad and constitutive tissue expression pattern of MIF in humans [82]. The respective study however noted a higher expression in tissues directly exposed to the natural environment such as the lung, the epithelial lining of the skin, and the gastrointestinal tracts [82]. In the so far conducted two mollusc studies high expression was also found especially in the hepatopancreas ( = digestive gland), mantle and gill tissue [83], [84] which are tissues highly exposed to the surrounding environment. Stimulation by different microbial associated molecular patterns (MAMPs, e.g. LPS, PGN) or bacteria resulted in increased MIF expression in *Chlamys farreri* [84] and *Haliotis diversicolor* [83] indicating the involvement of MIF in innate immune system pathways also in lower invertebrates.

#### 3.1 Tumor necrosis factor superfamily

TNF-α is a soluble cytokine with pleiotropic action. It is a member of the tumor necrosis factor super family (TNF) and their receptors (TNFR), which form a large group in the vertebrate system within various immune system pathways [85]. In bivalves several studies described the response of hemocytes on vertebrate TNF-α and hypothesized the occurrence of this cytokine and related receptors. However, sequence data for invertebrates are still scarce and incomplete. A first TNF-α homologue was identified in the gastropod *Haliotis discus discus* [86] but further factors and receptors still awaiting discovery in the mollusca. The *M. edulis* transcriptome does not contain a TNF-α orthologue, however 12 TNF superfamily members were found according to their conserved TNF_2 protein domain (domain ID: PS50049) using PROSITE [87] (Table S5). Eight members contain additional transmembrane domains, and 9 contain TNF-receptor domains, of which 3 have an additional transmembrane and DEATH domain (Table S5). Further the *M. edulis* transcriptome holds sequences encoding for TNF receptor adaptor proteins such as the DEATH domain containing FADD (*#HE609077*) and five TRAF proteins (*#HE609055*, *HE609056*, *HE609057*, *HE609058*, *HE609075*) (Table S4). No ortholog for TRADD was identified which is in line with reports from other invertebrate studies [88]. In invertebrates like *D. melanogaster* or the sea urchin *S. purpuratus* few TNF and TNFR could be identified whereas in *B. floridae* comparable high numbers like in vertebrates were reported [11] which lead to the hypothesis of the TNF expansion being a chordate innovation [89]. However, the *M edulis* transcriptome with a high number of TNF related contigs (Table S5) and corresponding adapters (Table S4) suggests that this family already plays an important role in lophotrochozoan species.

The LPS-induced TNF-alpha factor LITAF is a transcription factor which controls TNF alpha gene expression. The gene was indentified in various vertebrates and invertebrates, including bivalves [90], [91], [92]. In bivalves, a single LITAF gene per bivalve species was reported for the scallop *Chlamys farreri* [90] and the oysters *Pinctada fucata* [91] and *C. gigas* [92]. Tissue gene expression pattern in the different species was found to be quite variable with generally highest expression in gill and intestinal organs. LITAF expression in vertebrates and bivalves can be induced by LPS stimulation [91], [93], [94], indicating an immune system function. In the *M. edulis* transcriptome 24 fragments with a LITAF domain and tBLASTx hit (UniprotKB/Swissprot, e≤10^−3^) for LITAF were found (Table S6). Tissue expression deduced from the transcriptome data showed high expression in digestive gland and hemocytes. As no specific gill tissue transcriptome was generated qRT-PCR analysis of one LITAF transcript (*#*HE609047) was carried out. This verified a high expression level in digestive gland tissue and hemocytes and additionally demonstrated high expression in gill tissue (Figure 9). All three tissues are in close contact to the surrounding environment and the gill and digestive gland have to defend their large surfaces against microbial infection with fast recognition and signal transduction. These results, together with the high number of TNF and TNFR transcripts indicate that the TNF pathway presumably plays an important role in the first line of defense in *M. edulis*.

#### 3.2 JAK/STAT

An evolutionary conserved pathway induced by cytokines is the JAK/STAT pathway. JAKs are activated by cytokine receptors and phosphorylate STATs, which then translocate into the nucleus and modulate expression of various genes involved in immune reactions, such as cytokines. Previous studies in *Mytilus edulis* using mammalian STAT antibodies and IFN as a stimulant already suggested the occurrence of the JAK/STAT pathway in bivalves. In lower invertebrates like *D. melanogaster* and *Anopheles gambiae*, key players of the JAK/STAT pathway have already been identified and were found to be involved in immune response processes [95]. As expected, contigs with a high similarity to JAK 2 (*#*HE609094) and STAT (*#*HE609095, HE609096, HE609097) (Table S4) are present in *M. edulis*. Further 2 putative transcripts of the suppressor of cytokine signaling (SOCS) are found (*#*HE609098, HE609099), which is a negative regulator of the cytokine signal within the JAK/STAT pathway. The control of cytokine production and other immune responses is crucial to prevent excess inflammatory processes which can also be harmful for the host. SOCS acts in a feedback-loop in such a way that its expression is up-regulated after STAT activation. The investigation of SOCS (*#*HE609098) in the tissue panel demonstrated highest expression in gill tissue (Table S4). Similar results were found for the single so far described mollusc SOCS gene in the gastropod *Haliotis discus discus* [96]. These results give evidence for the presence of cytokine-like proteins in *M. edulis* and other molluscs, and gill tissue might be a good candidate tissue to look for further transcripts. Indeed, as reported above, *Me2*-IL-17 was expressed highest in gill tissue. Yet no convincing transcript coding for an interleukin- or interferon-receptor could be identified in the *M. edulis* transcriptome or other studies. Thus, the respective cytokine receptor for the activation of JAK in *M. edulis* and other bivalves still remains to be discovered.

#### 3.3 Antimicrobial peptides

Like cytokines, antimicrobial peptides (AMPs) are downstream targets after recognition of foreign particles. AMPs are widely expressed in invertebrates to vertebrates to defend against bacteria and fungi, either by directly killing or slowing growth of the attackers. In vertebrates, and presumably also in invertebrates, they further act as modulators of the immune system by e.g. stimulating cytokine release or chemotaxis [97]. In molluscs, the occurrence and function of AMPs has been especially well studied in *M. edulis* and *M. galloprovincialis*. The so far identified AMPs can be sorted into the four groups: defensins, mytilins, myticins and mytimycins [98], [99]. The different peptide groups show distinct antimicrobial properties against gram-positive and gram-negative bacteria and fungi. Several transcripts of the known AMPs i.e. myticins (5 contigs), mytilins (13 contigs), mytimycins (7 contigs) and defensins (8 contigs) are found in the *M. edulis* transcriptome (Table S7). Additionally 5 transcripts of a just recently identified AMP for *Mytilus galloprovincialis* [7], a big defensin, are present (Table S7, Figure S3). All AMP contigs in *M. edulis* are covered by a high total number of 41274 reads (Table S7). Compared to the much lower number of 13276 reads in all other immune related transcripts (Table S4), this strongly indicates the important role of these AMPs in the *Mytilus* immune system.

### 4. Complement system

An ancient pro-inflammatory and microbial destruction system is the complement system. Genomes of *Drosophila* and *Caenorhabditis* lack components of the complement system, which previously led to the assumption that the complement pathway is restricted to the vertebrate system. However the recent findings of complement factors in genomes and transcriptomes of other protostomes and early metazoans rather indicates a gene loss in the ecdysozoan lineage and instead imply an ancient origin of the complement pathway [100].

DNA and mRNA sequence findings of the C3 complement factor, which is the central element of all three known complement pathways (classical, alternative, lectin) [100], have been reported for several invertebrates like cnidarians [10], [101], the protostomian horseshoe crab [102] as well as the deuterostomian echinoderms [103], [104] and tunicates [38]. Previous identification of mollusc C3 orthologs in the cephalopod *Euprymna scolopes* [105] and bivalve *Venerupis decussates* [106] indicated the possibility of a complement system also in molluscs. In the *M. edulis* transcriptome, two fragments (*#*HE609863, HE609109) with high similarity to the C3 complement factor could be identified (Table S4). While the immune system function of C3 was impressively demonstrated in the higher invertebrates [102], [103], however, functional analysis in molluscs is still scarce and awaits further investigation. As further outlined by Nanoko [100] the composition of the complement system in molluscs is still unresolved. In the *M. edulis* and other mollusc databases, none of the other central components of the mammalian complement cascade (Bf, C2, C4, C5) could be identified next to C3. Only for the factor B (Bf), a component of the alternative pathway, a single transcript is described for the bivalve *Ruditapes decussatus* [106]. Due to alterations in the sequence structure, the function of the bivalves' Bf gene as a C3 convertase in the complement pathway was, however, questioned by other researchers [100]. In the large *M. edulis* transcriptome no Bf-like ortholog could be found by annotation or via protein domain analysis.

From the components of the last membrane attack complex (MAC) only a sole fragment with similarity to the C6 complement factor is present in *M. edulis* (*#*HE609110). C6, C7, C8 and C9 form the membrane attack complex (MAC) by constructing a pore in the pathogenic/foreign cell membrane, thereby destroying it. Sequence findings of the C6 complement factor and other MAC factors have been described only in the deuterostome lineage, including first hints in marine invertebrates, the sea urchin *S. purpuratus* [107] and the tunicate *Ciona intestinalis* [38]. The lack of MAC members in *M. edulis* goes hand in hand with other transcriptome/genome studies of lower marine invertebrates [10], [105]. The transcriptome, however, contained 19 fragments exhibiting a single Membrane-Attack-Complex/Perforin domain (MACPF, Pfam domain PF01823) which might belong to the complement system (Table S8) [108]. In all, the current data corroborate Nonaka's hypothesis that the complement system of protostomes might be quite different when compared to the vertebrate or cnidarian system [100].

### 5. Apoptosis/Autophagy

Cell death is a fundamental response to immune stress and different forms of cellular destruction can be distinguished: apoptosis, autophagy and necrosis. The processes of apoptosis and autophagy are well controlled and extensively described pathways [109], [110]. Characteristics of apoptosis on the cellular level, like the fragmentation of the nucleus or translocation of phosphatidylserine to the outer membrane, have been described in a variety of lower invertebrates including molluscs. Sequence information of genes involved in these processes is, however, still rather scarce [111]. In 2006 a complex apoptotic toolkit was discovered in the sea urchin genome, resembling the expanded apoptosis machinery in the vertebrate lineage. This corroborated the hypothesis that the apoptotic pathway increased in complexity towards the deuterostome lineage, whereas it simplified in the protostome group, as described in *D. melanogaster* and *C. elegans*. In the present study, however, the finding of various transcripts for apoptosis related genes in the *M. edulis* transcriptome (Table S4) illustrates once more that the simplification of pathways may not be the case for all protostome lineages. As described above, a high number of TNF receptor like transcripts were identified, which are involved in apoptotic processes, as well as various members of the BCL2 family and the apoptosis-inducing factor family (AIFs, AIF: *#*HE609124, AFL: *#*HE609125). Further, the *M. edulis* transcriptome contains 52 caspase-like transcripts with a caspase domain (Peptidase C14, Pfam domain PF00656). 5 of these transcripts have an additional CARD domain and one an additional BIR domain (Table S9). Such domain organization hints towards the occurrence of initiator and effector caspases in *M. edulis* and underlines the impression that a complex apoptosis machinery is already present in this mollusc.

In contrast to apoptosis, autophagy does not necessarily lead to cell death but is rather characterized by the formation of double-membrane vesicles which enclose unwanted particles and organelles, so called autophagosomes, which fuse with lysosomes for enzymatic degradation of enclosed contents. Main players here include mTOR (target of rapamycin) which is a key inhibitor of autophagy, and the large group of autophagy-related genes (ATGs), responsible for autophagy induction and autophagosome formation. Recent studies promote the importance of autophagy in the immune system, be it as a downstream effect of TLR recognition or other PRRs [112], [113], or in the involvement of LC3 (ATG8) in phagocytosis of pathogens [114]. In marine invertebrates autophagic processes have mainly been investigated within the context of ecotoxicology or in oxidative stress scenarios induced by various environmental stressors [115]. The involvement in immune system function is less explored and the lack of sequence information hampered the investigation of gene expression until now. Within the current transcriptome we could now identify transcripts for 10 ATGs, Beclin 1 and mTOR (Table S4). These findings point towards a well developed autophagy machinery. The sequence information obtained in the present study will help to investigate this presumably evolutionary early immune pathway, postulated to represent a very initial cell-autonomous defense of early eukaryotic cells against microbes [112].

The *de novo* transcriptome assembly always results in fragmented transcripts of genes. Previous investigations of immune system parameters in marine invertebrate imply a high diversity within some genes families e.g. C1q domain containing genes in bivalves [6] or TLRs, NLRs or the 185/333 system in sea urchins [116].The high number of transcript fragments reported for some genes families in the present study, such as C1q or TNF domain containing contigs, LITAF-like or BCL2-like contigs, most likely thus represent real single genes, but may still partly result from assembly artifacts or represent splice variants of the same gene. Future studies, which go into more detail of the different genes families, should aim for a further verification of a transcripts' origin. Clustering approaches and multiple sequence alignments could serve as useful tools to test whether contigs indeed represent real single genes. The release of the recently sequenced first bivalve genome from the oyster *Crassotrea gigas* will certainly give further insight into the diversity of the bivalves' immune system.

### Tissue atlas of selected immune repertoire transcripts in Mytilus edulis

Tissue expression of selected contigs with putative immune function in *Mytilus edulis* was analyzed to get a first insight whether molecular patterns of these contigs i) reflect a tissue or cell specific function e.g. immune defense for hemocytes, or digestion and therefore exposure to microbial loads in the digestive gland, or ii) display the grade of exposure to the surrounding environment e.g. the constant exposure of gill tissue to inflowing seawater and accompanied microbes. 15 of the 17 selected putative immune related transcripts identified the gill tissue and hemocytes as major sites of immune defense (Figure 9). Gills are in close contact to the surrounding sea water medium and the present results imply that this organ might not only be important for feeding and respiration. Moreover, it does not mediate its immune defense purely by physical factors such as a pronounced mucus layer, but may play a major role in the first recognition of unwanted particles in the environment and the initiation of an immune response The high expression of transcripts known to be involved in several immune pathways such as I*κ*B, *NF-κB* and TRAF 6 as well as interleukin-17 corroborate this assumption. Highest transcript levels of LBP and PGRPs were demonstrated in hemocytes. Both genes are important in the recognition and direct destruction of bacteria. Interestingly, compared to hemocytes and gill tissue, the digestive gland was not characterized by exclusively high expression values of any of the investigated contigs, which was expected due to the high exposure to microbes and other foreign particles during feeding. The current analysis may thus give a first hint towards the differential immunological importance of the barrier organs of *M. edulis* and may stimulate functional investigations describing the role of different immune pathways under physiological and pathological (e.g. infection) conditions.

### Conclusion

The marine mussel *Mytilus edulis* has a wide geographic and ecosystem distribution and is a keystone species of many coastal marine communities. The in-depth investigation of a *M. edulis* transcriptome revealed a complex immune repertoire including a high number of innate immune recognition receptors and downstream pathway members, a sophisticated TNF, autophagy and apoptosis system, as well as putative cytokines such as interleukin-17 and several macrophage inhibitory factors (MIFs). The results of this study partly contrast with findings obtained for ecdysozoans and other studies in lophotrochozoans which reported a less complex immune repertoire. Especially the number of 27 TLR exceeds the previous finding of maximal 2 TLR transcripts per bivalve species. Together with the high number of TLR pathway members this indicates a well-conserved TLR pathway in bivalves. A higher number of genes were annotated by species of the deuterostome clade compared to ecdysozoan protostome annotations. Although the absence of NLRs in *Mytilus* is in line with findings obtained for ecdysozoans, together with the high number of TNF family members, a seemingly complex apoptosis machinery and the presence of LBP and the complement system, this points towards a closer relation to the deuterostome clade due to the basal position of lophotrochozoans within protostomes. This hypothesis is corroborated by the phylogenetic comparison of *M. edulis* randomly selected genes with 23 species ranging from *Amphimedon* to *Homo sapiens*. *Mytilus edulis* and the other investigated lophotrochozoans (*Aplysia californica*, *Lottia gigantea*, *Helobdella robusta*, *Capitella teleta*) show a closer evolutionary distance to the deuterostomes than the ecdysozoan clade. Taken together, our study provides an unprecedented in-depth analysis of the *Mytilus edulis* transcriptome and demonstrates that bivalves indeed represent important model organisms that may lead to novel insights into the phylogeny of the immune system. The inventory of the *Mytilus* immune system put forward in the current study will form a sound basis for functional studies, which are required to assess the exact role of the individual elements of the transcriptome. In addition to functional studies the data enable to study SNP patterns and allele frequencies in different *Mytilus* populations subjected to high selective pressure in order to systematically elucidate the extent of selection on distinct immune pathways [117].

## Supporting Information

Figure S1 **Comparison of** ***Mytilus edulis*** **transcriptome sequences with previously published** ***Mytilus*** **sequences.** BLASTn comparison with contigs and singletons of the *M. edulis* transcriptome (251098 sequences (seq.): 74622 contigs, 176476 singletons) with previously published *Mytilus* sequences using different cut-off e-values. Shown is the percentage of sequences of published datasets highly similar to sequences of the new *M. edulis* database. A) Comparison with *Mytilus edulis* sequences from 1) Craft *et al.* unassembled singletons (5036 seq.), 2) Craft et al. newly assembled contigs (5742 sequences) and 3) downloaded from NCBI (271 seq.). B) Comparison with *Mytilus galloprovincialis* sequences from 1) Craft *et al.* unassembled singletons (40972 seq.), 2) Craft et al. newly assembled contigs (12827 sequences), and 3) downloaded from NCBI (19579 seq.). C) Comparison against all sequences published for the genus *Mytilus* (127591 seq.) downloaded from NCBI and taken from Craft *et al.* 2010. *Vice versa*: D) Percentage of sequences of the new *M. edulis* database, 1) contigs 2) singletons, which are found in previously published datasets for the genus *Mytilus* (127591 seq.).
(TIF)

Figure S2 **Deduced amino acid sequence of NF-κB-like contigs of the blue mussel** ***Mytilus edulis*** **.** In mammals the five proteins of the NF-κB transcription factor family (p65 (RelA), RelB, c-Rel, p105/p50 (NF-κB1), p100/52 (NF-κB2)) form distinct transcriptionally active homo- and heterodimeric complexes. The p50/p65 dimer is the most prominent heterodimer in most cell types [119], and exhibit a conserved DNA-binding/dimerization domain (‘RGLRFRYECE’), Rel homology domain (RHD), and a nuclear localization signal (NLS, ‘KRKR’). In most unstimulated cells the NF-*κ*B dimer is associated with I*κ*B in the cytosol until phosphorylation of I*κ*B by IKK, thereafter NF-*κ*B translocates into the nucleus. The two *M. edulis* contigs HE609048 and HE609050 show high similarity to NF-κB p65 and p56 and contain the conserved region of the RHD and the NLS domain (shaded).
(TIF)

Figure S3 **Conserved motifs in** ***M. edulis*** **big defensin transcripts identified in** ***Mytilus edulis*** **.** The amino acid sequence of 5 *Mytilus edulis* big defensins are compared with big defensin amino acid sequences from *Argopecten irradians* (AiBD, GenBank accession no. DQ334340) and *Venerupis philippinarum* (VpBD, GenBank accession no. HM562672). Further the conserved motif of big defensins is aligned [120]. In the bay scallop *Argopecten irradians* [121] and the clam *Venerupis philippinarum* [120] this AMP exhibited high antimicrobial activity against gram positive and gram negative bacteria, and was most abundant in gill tissue and hemocytes [121]. When translated into amino acids, the 5 transcripts of *M. edulis* share the conserved C-X6-C-X3-C-X13(14)-C-X4-C-C pattern in the defensin domain [120].
(TIF)

Table S1 Information of sample composition within the different transcriptome runs as well as of sequence data generated with *FLX or Titanium chemistry before and after quality control. Data after quality control were used for the assembly (see table S2).
(DOC)

Table S2 **Assembly strategy of the** ***Mytilus edulis*** **transcriptome.** Cleaned and quality controlled reads were initially assembled with either Celera (1. Assembly) or NEWBLER (2. Assembly). Resulting Contigs (C) and singletons (S) were subsequently further assembled in multiple rounds (R) using TGICL (Cap3) assembler and AMOS. For Run numbers 115–284 see table S1.
(DOC)

Table S3 **Information about genome sources of the different species used for phylogenetic analysis.**
(DOC)

Table S4 **Putative** ***M. edulis*** **immune relevant genes identified via BLAST analysis.** A) Sequence and domain information. Domain information of *M. edulis* contigs was deduced by amino-acid sequence investigation using SMART [27] and NCBI dart [28] with default thresholds. B) Comparison with selected species. Putative *M. edulis* immune genes were compared via tBLASTx against the nr/nt NCBI database of the classical model organisms *Homo sapiens* (taxid 9606), *Mus musculus* (taxid 10090), *Drosophila melanogaster* (taxid 7227), *Caenorhabditis elegans* (taxid6239) as well as molluscs (taxid6447), bivalvia (taxid6544) and *Mytilus* (taxid 6548). nd = no annotated blast hit detectable in nr/nt NCBI.* = tBLAStx against UniProtKB.
(DOC)

Table S5 **TNF related** ***M. edulis*** **transcripts with conserved TNF or TNFR domains identified by PROSITE.**
(DOC)

Table S6 ***M. edulis*** **contigs with high similarity to LPS induced TNF-alpha factor (LITAF) - like proteins of other organisms.**
(DOC)

Table S7 **Antimicrobial peptides identified in** ***M. edulis*** **.**
(DOC)

Table S8 **19 MACPF domain bearing contigs of** ***M. edulis*** **.** 5 of these fragments showed similarity to macrophage expressed gene-1, and 3 to apextrin, while 11 fragments could not be annotated. Proteins containing the MACPF domain but lacking other domains of the MAC complex members were reported in several other lower invertebrates (for review see, [108]). Some are known to belong to genes with strong hemolytic function like the hemolytic toxins of the nematocysts of the sea anemone *Phyllodiscus semoni* [108], and are therefore put in close relation to the vertebrate MAC members. Whether the 11 non-annotated MACPF containing fragments of *M. edulis* belong to the complement systems needs further sequence and functional analysis.
(DOC)

Table S9 **Caspase-like contigs of** ***M. edulis*** **.**
(DOC)

File S1 **Alignment (aln format) used for phylogenetic analysis.**
(ALN)

File S2 **Alignment (phy format) used for phylogenetic analysis.**
(PHY)
