# Supplementary figures and images for: Massively Parallel RNA Sequencing Identifies a Complex Immune Gene Repertoire in the lophotrochozoan Mytilus edulis
Source: PLoS One. 2012 Mar 20;7(3):e33091. doi: 10.1371/journal.pone.0033091 (PMC3308963; doi:10.1371/journal.pone.0033091)

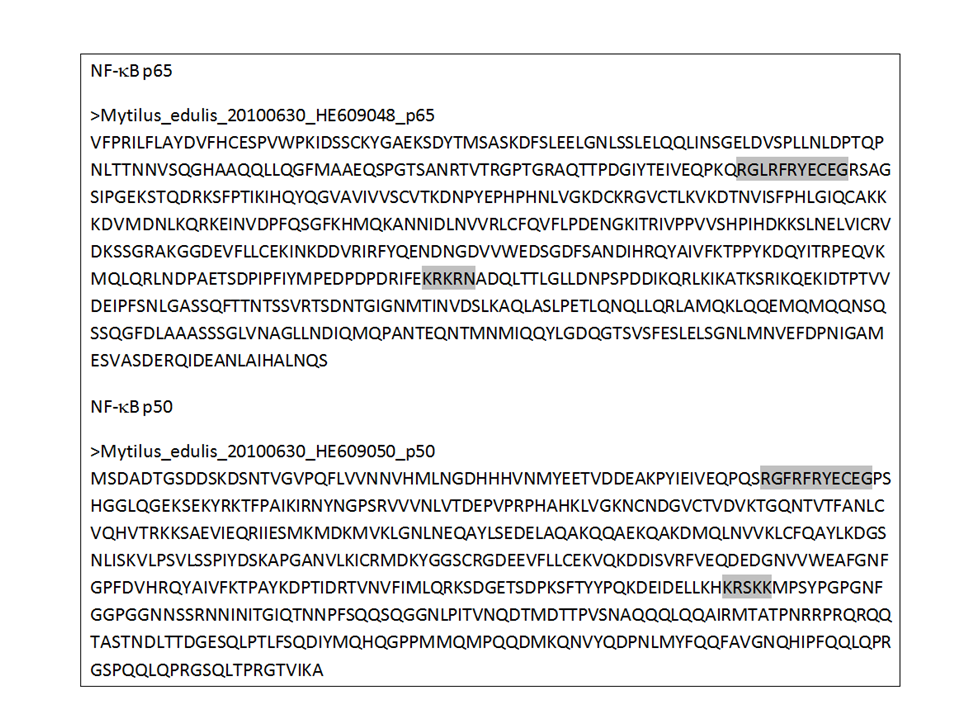

Supplement: Figure S2 — Deduced amino acid sequence of NF-κB-like contigs of the blue mussel Mytilus edulis . In mammals the five proteins of the NF-κB transcription factor family (p65 (RelA), RelB, c-Rel, p105/p50 (NF-κB1), p100/52 (NF-κB2)) form distinct transcriptionally active homo- and heterodimeric complexes. The p50/p65 dimer is the most prominent heterodimer in most cell types [119], and exhibit a conserved DNA-binding/dimerization domain (‘RGLRFRYECE’), Rel homology domain (RHD), and a nuclear localization signal (NLS, ‘KRKR’). In most unstimulated cells the NF-κB dimer is associated with IκB in the cytosol until phosphorylation of IκB by IKK, thereafter NF-κB translocates into the nucleus. The two M. edulis contigs HE609048 and HE609050 show high similarity to NF-κB p65 and p56 and contain the conserved region of the RHD and the NLS domain (shaded). (TIF) [file pone.0033091.s002.tif]
