# Supplementary material for: Massively Parallel RNA Sequencing Identifies a Complex Immune Gene Repertoire in the lophotrochozoan Mytilus edulis
Source: PLoS One. 2012 Mar 20;7(3):e33091. doi: 10.1371/journal.pone.0033091 (PMC3308963; doi:10.1371/journal.pone.0033091)
Supplement: Table S1 — Information of sample composition within the different transcriptome runs as well as of sequence data generated with *FLX or Titanium chemistry before and after quality control. Data after quality control were used for the assembly (see table S2). (DOC) [file pone.0033091.s004.doc]

|  | Sample information | | | | Roche sff file before quality clip and cleaning | | | After quality clip and cleaning | | |
| --- | --- | --- | --- | --- | --- | --- | --- | --- | --- | --- |
| **GS FLX Filename** | **Treatment** | **Duration of treatment** | **Tissue** | **Nr of individuals within the sample** | **Read number** | **Average Length (bp)** | **Mb** | **Read number** | **Average Length (bp)** | **Mb** |
| 115_Mytilus_EST_unstimuliert* | control | 4h | digestive gland | 1 | 5378 | 108 | 0,58 | 4669 | 110 | 0,51 |
| 116_Mytilus_EST_LPS_stimuliert* | LPS | 4h | digestive gland | 2 | 6141 | 114 | 0,7 | 5365 | 115 | 0,61 |
| 138_M_edulis_control* | control | 4h | ” digestive gland | 1 | 42383 | 246 | 10,42 | 40911 | 235 | 9,62 |
| 139M_edulis_LPS* | LPS | 4h | digestive gland | 2 | 48128 | 248 | 11,97 | 46739 | 240 | 11,22 |
| 152_M_edulisCtrlAllTissues_081024* | control | 5 days | gill, foot , adductor muscle, digestive gland | 2 | 174340 | 224 | 39,07 | 164782 | 216 | 35,59 |
| 153_M_edulisStressAllTissues_081024* | heat, anoxia, injury, LPS | 5 days | gill, foot , adductor muscle, digestive gland | 8 | 181101 | 217 | 39,32 | 167444 | 206 | 34,56 |
| 153_M_edulisStressAllTissues_081024_Titration13* | ” | ” | ” | ” | 999 | 230 | 0,23 | 925 | 217 | 0,2 |
| 153_M_edulisStressAllTissues_081024_Titration14* | ” | ” | ” | ” | 2082 | 226 | 0,47 | 1905 | 215 | 0,41 |
| 153_M_edulisStressAllTissues_081024_Titration15* | ” | ” | ” | ” | 3701 | 221 | 0,82 | 3372 | 211 | 0,71 |
| 153_M_edulisStressAllTissues_081024_Titration16* | ” | ” | ” | ” | 3403 | 191 | 0,65 | 2984 | 189 | 0,56 |
| 185_Mytilus_edulis_TitaniumTest01 | 2-4% DSS | 9 days | digestive gland | 3 | 14 | 152 | 0 | 12 | 157 | 0 |
| 185_Mytilus_edulis_TitaniumTest02 | ” | ” | ” | ” | 315 | 166 | 0,05 | 287 | 168 | 0,04 |
| 185_Mytilus_edulis_TitaniumTest03 | ” | ” | ” | ” | 118 | 214 | 0,02 | 111 | 213 | 0,02 |
| 185_Mytilus_edulis_TitaniumTest04 | ” | ” | ” | ” | 134 | 231 | 0,03 | 127 | 231 | 0,02 |
| 186_Mytilus_edulis_TitaniumTest05 | control | 9 days | digestive gland | 3 | 4894 | 191 | 0,93 | 4516 | 189 | 0,85 |
| 186_Mytilus_edulis_TitaniumTest06 | ” | ” | ” | ” | 734 | 254 | 0,18 | 699 | 253 | 0,17 |
| 186_Mytilus_edulis_TitaniumTest07 | ” | ” | ” | ” | 712 | 169 | 0,12 | 669 | 171 | 0,11 |
| 186_Mytilus_edulis_TitaniumTest08 | ” | ” | ” | ” | 978 | 235 | 0,23 | 938 | 234 | 0,21 |
| 221_FZUWXHW01 | 2-4% DSS | 9 days | digestive gland | 3 | 1893 | 198 | 0,37 | 1815 | 188 | 0,34 |
| 221_FZUWXHW02 | ” | ” | ” | ” | 1912 | 195 | 0,37 | 1848 | 185 | 0,34 |
| 221_FZUWXHW03 | ” | ” | ” | ” | 3700 | 194 | 0,72 | 3543 | 185 | 0,65 |
| 221_FZUWXHW04 | ” | ” | ” | ” | 3050 | 190 | 0,57 | 2925 | 181 | 0,53 |
| 221_FZUWXHW05 | ” | ” | ” | ” | 2353 | 204 | 0,48 | 2269 | 194 | 0,44 |
| 221_FZUWXHW06 | ” | ” | ” | ” | 3665 | 205 | 0,75 | 3557 | 195 | 0,69 |
| 221_FZUWXHW07 | ” | ” | ” | ” | 5094 | 200 | 1,02 | 4947 | 190 | 0,94 |
| 221_FZUWXHW08 | ” | ” | ” | ” | 1920 | 187 | 0,36 | 1838 | 180 | 0,33 |
| 221_Mytilus_edulis_090731_FZZ6VRZ01 | ” | ” | ” | ” | 255845 | 188 | 48,18 | 248561 | 179 | 44,61 |
| 222_FZUWXHW09 | control | 9 days | digestive gland | 3 | 1107 | 186 | 0,2 | 1047 | 179 | 0,18 |
| 222_FZUWXHW10 | ” | ” | ” | ” | 290 | 183 | 0,05 | 280 | 175 | 0,04 |
| 222_FZUWXHW11 | ” | ” | ” | ” | 4651 | 200 | 0,93 | 4486 | 189 | 0,85 |
| 222_FZUWXHW12 | ” | ” | ” | ” | 5134 | 197 | 1,01 | 4949 | 187 | 0,92 |
| 222_FZUWXHW13 | ” | ” | ” | ” | 2165 | 212 | 0,46 | 2087 | 200 | 0,41 |
| 222_FZUWXHW14 | ” | ” | ” | ” | 197 | 189 | 0,03 | 189 | 183 | 0,03 |
| 222_FZUWXHW15 | ” | ” | ” | ” | 263 | 198 | 0,05 | 258 | 189 | 0,04 |
| 222_FZUWXHW16 | ” | ” | ” | ” | 4920 | 193 | 0,95 | 4750 | 186 | 0,88 |
| 222_Mytilus_edulis_090731_FZZ6VRZ02 | ” | ” | ” | ” | 255407 | 201 | 51,36 | 248633 | 191 | 47,49 |
| 230_Mytilus_edulis_DSS_090912 | 2-4% DSS | 9 days | digestive gland | 3 | 267099 | 330 | 88,24 | 254523 | 318 | 81,1 |
| 231_Mytilus_edulis_Control_090912 | control | 9 days | digestive gland | 3 | 171477 | 292 | 50,15 | 161712 | 283 | 45,79 |
| 271_Mytilus_edulis_4000_MA | 4000 µatm | 8 weeks | inner mantle | 8 | 139609 | 358 | 49,98 | 129360 | 361 | 46,79 |
| 272_Mytilus_edulis_380_MR | 380 µatm | 8 weeks | mantle rim | 11 | 161670 | 350 | 56,7 | 149218 | 356 | 53,17 |
| 273_Mytilus_edulis_380_MA | 380 µatm | 8 weeks | inner mantle | 12 | 183873 | 381 | 70,07 | 172837 | 382 | 66,18 |
| 274_Mytilus_edulis_4000_MR | 4000 µatm | 8 weeks | mantle rim | 12 | 162922 | 349 | 56,87 | 151290 | 351 | 53,14 |
| 283_Mytilus_edulis_haemozyten_control | control | 4 hours | hemocytes | 32 | 189366 | 330 | 62,64 | 181117 | 320 | 58,04 |
| 284_Mytilus_edulis_haemozyten_flagellin | 2.5µg/ml flagellin | 4 hours | hemocytes | 32 | 217695 | 335 | 73,1 | 208947 | 330 | 68,97 |
