# Supplementary material for: Massively Parallel RNA Sequencing Identifies a Complex Immune Gene Repertoire in the lophotrochozoan Mytilus edulis
Source: PLoS One. 2012 Mar 20;7(3):e33091. doi: 10.1371/journal.pone.0033091 (PMC3308963; doi:10.1371/journal.pone.0033091)
Supplement: Table S2 — Assembly strategy of the Mytilus edulis transcriptome. Cleaned and quality controlled reads were initially assembled with either Celera (1. Assembly) or NEWBLER (2. Assembly). Resulting Contigs (C) and singletons (S) were subsequently further assembled in multiple rounds (R) using TGICL (Cap3) assembler and AMOS. For Run numbers 115–284 see table S1. (DOC) [file pone.0033091.s005.doc]

| Round | Assembler/Mapper | Input | percent_identity | min_overlap |  |
| --- | --- | --- | --- | --- | --- |
| 1. Assembly |  |  |  |  |  |
| 1 | Celera 5.3 | Reads(Runs: 115-231) | default settings | default settings |  |
| 2 | Celera 5.3 | Singletons( Round: 1) | ” | ” |  |
| 3 | Celera 5.3 | Singletons(Round: 2) | ” | ” |  |
| 4 | Celera 5.3 | Singletons(Round: 3) | ” | ” |  |
| 5 | Celera 5.3 | Singletons(Round: 4) | ” | ” |  |
| 6 | Cap3 | Singletons(Round: 5, 100.000 Seq) | 90 | 40 |  |
| 7 | Cap3 | Singletons(Round: 5, 100.000 Seq) | 90 | 40 |  |
| 8 | Cap3 | Singletons(Round: 5, 100.000 Seq) | 90 | 40 |  |
| 9 | Cap3 | Singletons(Round: 5, 90.804 Seq) | 90 | 40 |  |
| 10 | Cap3 | Singletons(Round: 6) | 100 | 200 |  |
| 11 | Cap3 | Singletons(Round: 7) | 90 | 40 |  |
| 12 | Cap3 | Singletons(Round: 8) | 94 | 100 |  |
| 13 | Cap3 | Singletons(Round: 9) | 98 | 180 |  |
| 14 | Cap3 | Singletons(Round: 10) | 90 | 40 |  |
| 15 | Cap3 | Contigs(Round: 1-11) | 98 | 200 |  |
| 16 | Cap3 | Contigs(Round: 12) | 98 | 150 |  |
| 17 | Cap3 | Contigs(Round: 13) | 98 | 100 |  |
| 18 | Cap3 | Contigs(Round: 14) | 96 | 80 |  |
| 19 | Cap3 | Contigs(Round: 15) | 99 | 40 |  |
| 20 | Cap3 | Contigs(Round: 16) | 97 | 50 |  |
| 21 | Cap3 | Contigs(Round: 17) | 96 | 40 |  |
| 22 | Cap3 | Contigs(Round: 18) | 94 | 40 |  |
| 23 | Cap3 | Contigs(Round: 19) | 92 | 40 |  |
| 24 | Cap3 | Contigs(Round: 20) | 90 | 40 |  |
| 25 | Amos | Reads(Input: all reads; Backbone: C+S of R21): | Contigs: 49397, Average length: 465 bp; Singletons: 198756, Average length: 237 bp | | |
| 2. Assembly |  |  |  |  |  |
| 1 | Newbler(cDNA) | Reads(Runs: 271-284) | 90 | 40 |  |
| 2 | Cap3 | Singletons(Round: 1) | 96 | 60 |  |
| 3 | Cap3 | Singletons(Round: 2) | 94 | 40 |  |
| 4 | Cap3 | Contigs(Round: 1-3; old_Assembly) | 98 | 200 |  |
| 5 | Cap3 | Contigs+Singletons(C+S, Round: 4) | 96 | 140 |  |
| 6 | Cap3 | C+S (Round: 5) | 96 | 80 |  |
| 7 | Cap3 | C+S (Round: 6) | 96 | 40 |  |
| 8 | Amos | Reads(Input: all reads; Backbone: C+S of R7): | Contigs: 78929, Average length: 563 bp; Singletons: 253095, Average length: 298 bp | | |
| 9 | Cap3 | Singletons(Round: 8) | 96 | 60 |  |
| 10 | Cap3 | Singletons(Round: 9) | 94 | 40 |  |
| 11 | Cap3 | Contigs(Round: 8-10) | 96 | 160 |  |
| 12 | Cap3 | Contigs(Round: 11) | 98 | 100 |  |
| 13 | Cap3 | Contigs(Round: 12) | 98 | 40 |  |
| 14 | Cap3 | Contigs(Round: 13) | 96 | 40 |  |
| 15 | Cap3 | Contigs(Round: 14) | 94 | 40 |  |
| 16 | Amos | Reads(Input: all reads; Backbone: C of R15): | Contigs: 67384, Average length: 657 bp; Singletons: 234492, Average length: 274 bp | | |
| 17 | Cap3 | Singletons(Round: 16) | 96 | 60 |  |
| 18 | Cap3 | Contigs(Round: 16-17) | 98 | 40 |  |
| 19 | Cap3 | C+S (Round: 18) | 90 | 160 |  |
| 20 | Cap3 | C+S (Round: 19) | 90 | 100 |  |
| 21 | Cap3 | C+S (Round: 20) | 90 | 40 |  |
| 22 | Amos | Reads(Input: all reads; Backbone: C of R21): | Contigs: 62928, Average length: 678 bp; Singletons: 231486, Average length: 272 bp | | |
| 23 | Cap3 | Singletons(Round: 22) | 92 | 160 |  |
| 24 | Cap3 | Singletons(Round: 23) | 92 | 60 |  |
| 25 | Cap3 | Contigs(Round: 22-24) | 99 | 260 |  |
| 26 | Cap3 | C+S (Round: 25) | 96 | 200 |  |
| 27 | Cap3 | C+S (Round: 26) | 96 | 160 |  |
| 28 | Cap3 | C+S (Round: 27) | 90 | 160 |  |
| 29 | Cap3 | C+S (Round: 28) | 96 | 150 |  |
| 30 | Cap3 | C+S (Round: 29) | 80 | 300 |  |
| 31 | Cap3 | C+S (Round: 30) | 80 | 160 |  |
| 32 | Cap3 | C+S (Round: 31) | 80 | 160 |  |
| 33 | Cap3 | C+S (Round: 32) | 85 | 100 |  |
| 34 | Cap3 | C+S (Round: 33) | 90 | 60 |  |
| 35 | Cap3 | C+S (Round: 34) | 80 | 360 |  |
| 36 | Cap3 | C+S (Round: 35) | 80 | 160 |  |
| 37 | Cap3 | C+S (Round: 36) | 85 | 40 |  |
| 38 | Cap3 | C+S(R: 37); S(R: 24) | 90 | 60 |  |
| 39 | Cap3 | C+S(R: 38) | 90 | 100 |  |
| 40 | Cap3 | C+S(R: 39) | 90 | 160 |  |
| 41 | Cap3 | C+S(R: 40) | 90 | 260 |  |
| 42 | Cap3 | C+S(R: 41) | 90 | 40 |  |
| 43 | Cap3 | C+S(R: 42) | 85 | 260 |  |
| 44 | Cap3 | C+S(R: 43) | 85 | 160 |  |
| 45 | Cap3 | C+S(R: 44) | 80 | 460 |  |
| 46 | Cap3 | C+S(R: 45) | 90 | 40 |  |
| 47 | Amos | Reads(Input: all reads; Backbone: C of R21): | Contigs: 71907, Average length: 645 bp; Singletons: 206155, Average length: 287 bp | | |
| 48c | Cap3 | Contigs (R: 47) | 90 | 60 |  |
| 48s | Cap3 | Singletons (R: 47) | 90 | 160 |  |
| 49c | Cap3 | C+S(R: 48c) | 80 | 200 |  |
| 49s | Cap3 | Singletons (R: 48s) | 90 | 300 |  |
| 50c | Cap3 | C+S(R: 49c) | 80 | 140 |  |
| 50s | Cap3 | Singletons (R: 49s) | 90 | 360 |  |
| 51c | Cap3 | C+S(R: 50c) | 85 | 80 |  |
| 51s | Cap3 | Singletons (R: 50s) | 85 | 400 |  |
| 52s | Amos | Reads(Input: S of 47; BB: C of R48s-51s): | Contigs: 3236, Average length: 425 bp; Singletons: 165515, Average length: 269 bp | | |
| 52c | Cap3 | C+S(R: 51c) | 90 | 40 |  |
| 53 | Cap3 | C+S(R: 52c, 52s) | 90 | 60 |  |
| 54 | Cap3 | C+S(R: 53) | 80 | 260 |  |
| 55 | Cap3 | C+S(R: 54) | 80 | 460 |  |
| 56 | Cap3 | C+S(R: 55) | 85 | 40 |  |
| 57 | Cap3 | C+S(R: 56) | 85 | 60 |  |
| 58 | Cap3 | C+S(R: 57) | 85 | 100 |  |
| 59 | Amos | Reads(Input: all reads; Backbone: C of R58): | **Contigs: 74622, Average length: 645 bp; Singletons: 176476, Average length: 278 bp** | | |
