# Supplementary material for: Massively Parallel RNA Sequencing Identifies a Complex Immune Gene Repertoire in the lophotrochozoan Mytilus edulis
Source: PLoS One. 2012 Mar 20;7(3):e33091. doi: 10.1371/journal.pone.0033091 (PMC3308963; doi:10.1371/journal.pone.0033091)
Supplement: Table S3 — Information about genome sources of the different species used for phylogenetic analysis. (DOC) [file pone.0033091.s006.doc]

| **Species** | **Source** |
| --- | --- |
| *Mytilus edulis* | This paper |
| *Homo sapiens* | Ensembl Release 61 changed 01.02.11 |
| *Danio rerio* | Ensembl Release 61 changed 01.02.11 |
| *Trichoplax adhaerens* | JGI, version 1.0 |
| *Branchiostoma floridae* | JGI, version 1.0 |
| *Nematostella vectensis* | JGI, version 1.0 |
| *Xenopus tropicalis* | Ensembl Release 61 changed 01.02.11 |
| *Aplysia californica* | Broad Institute, Aplcal2.0 |
| *Hydra magnipapillata* | JGI, version 1.3 |
| *Caenorhabditis elegans* | Ensembl Release 61 changed 01.02.11 |
| *Drosophila melanogaster* | Ensembl Release 61 changed 01.02.11 |
| *Strongylocentrotus purpuratus* | SpBase version 2.6 |
| *Schmidtea mediterranea* | WUSTL, Schmidtea_mediterranea-3.1_ supercontigs.fa |
| *Capitella teleta* | JGI, version 1.0 |
| *Lottia gigantea* | JGI, version 1.0 |
| *Helobdella robusta* | JGI, version 1.0 |
| *Daphnia pulex* | JGI, version 1.0 |
| *Saccoglossus kowalevskii* | JGI version 1.3 |
| *Amphimedon queenslandica* | JGI, Aqu1.pep.fa |
| *Apis mellifera* | BeeBase, version 4.0 |
| *Tribolium castaneum* | Ensembl Release 61 changed 01.02.11 |
| *Pristionchus pacificus* | WormBase WS224 |
| *Ciona intestinalis* | Ensembl Release 61 changed 01.02.11 |
