# Supplementary material for: Massively Parallel RNA Sequencing Identifies a Complex Immune Gene Repertoire in the lophotrochozoan Mytilus edulis
Source: PLoS One. 2012 Mar 20;7(3):e33091. doi: 10.1371/journal.pone.0033091 (PMC3308963; doi:10.1371/journal.pone.0033091)
Supplement: Table S4 — Putative M. edulis immune relevant genes identified via BLAST analysis. A) Sequence and domain information. Domain information of M. edulis contigs was deduced by amino-acid sequence investigation using SMART [27] and NCBI dart [28] with default thresholds. B) Comparison with selected species. Putative M. edulis immune genes were compared via tBLASTx against the nr/nt NCBI database of the classical model organisms Homo sapiens (taxid 9606), Mus musculus (taxid 10090), Drosophila melanogaster (taxid 7227), Caenorhabditis elegans (taxid6239) as well as molluscs (taxid6447), bivalvia (taxid6544) and Mytilus (taxid 6548). nd = no annotated blast hit detectable in nr/nt NCBI.* = tBLAStx against UniProtKB. (DOC) [file pone.0033091.s007.doc]

**A:**

| Category | Gene | Domains expected | Domains identified | Contig length (bp) | Nr. of reads | Longest ORF (aa) containing protein domain/s | *M. edulis*  Accession |
| --- | --- | --- | --- | --- | --- | --- | --- |
| Transcription factors | *Me1-*IRF | IRF3, IRF | IRF3, IRF | 2046 | 19 | 469 | HE609042 |
|  | *Me2-*IRF | IRF3, IRF | IRF | 752 | 8 | 187 | HE609043 |
|  | *Me3-*IRF | IRF3, IRF | IRF | 1658 | 187 | 359 | HE609044 |
|  | *Me4-*IRF | IRF3, IRF | IRF | 775 | 6 | 153 | HE609045 |
|  | *Me5-*IRF | IRF3, IRF | IRF | 563 | 10 | 177 | HE609046 |
|  | LITAF | zf-LITAF like | zf-LITAF like | 1523 | 261 | 157 | HE609047 |
|  | *NF-κB* p65 | RhD, IPT | RhD, IPT | 3450 | 115 | 634 | HE609048 |
|  | *Me1*- *NF-κB* p50 | Arp, RhD, DEATH; ANK, IPT | 6 x ANK, DEATH | 3191 | 205 | 767 | HE609049 |
|  | *Me2*- *NF-κB* p50 | Arp, RhD, DEATH; ANK, IPT | RhD, IPT | 1774 | 57 | 543 | HE609050 |
| PRR pathway proteins | Caspase 3 subunit p12 | CASc | CASc | 3538 | 226 | 352 | HE609051 |
|  | *Me1-*TNFR | TNFR, TM, DEATH | TNFR,TM, DEATH | 1973 | 81 | 331 | HE609052 |
|  | *Me2-*TNFR | TNFR, TM, DEATH | 2x TNFR, TM, DEATH | 1245 | 140 | 335 | HE609053 |
|  | *Me3*-TNFR | TNFR, TM, DEATH | TNFR,TM, DEATH | 1564 | 236 | 341 | HE609054 |
|  | *Me1*-TRAF | RING, zf-TRAF, MATH | 2x zf-TRAF | 1020 | 40 | 288 | HE609055 |
|  | *Me2*-TRAF | RING, zf-TRAF, MATH | MATH | 666 | 4 | 194 | HE609056 |
|  | *Me3*-TRAF | RING, zf-TRAF, MATH | RING | 391 | 2 | 131 | HE609057 |
|  | *Me4*-TRAF | RING, zf-TRAF, MATH | zfTraf, MATH, Traf4 | 712 | 6 | 237 | HE609058 |
|  | *Me1*-I*κ*B | 5 x ANK | ANK | 1840 | 166 | 404 | HE609059 |
|  | *Me2*-I*κ*B | 6x ANK | ANK | 1663 | 539 | 392 | HE609060 |
|  | IKK | S_TKc, PKc_like | none | 1191 | 17 | 397 | HE609061 |
|  | *Me1*-caspase 8 | CASc, DED | CASc | 782 | 9 | 203 | HE609062 |
|  | *Me2*-caspase 8 | CASc, DED | CASc | 447 | 5 | 149 | HE609063 |
|  | ECSIT | ECSIT | ECSIT | 1371 | 30 | 420 | HE609064 |
|  | IRAK 4 | DEATH, STYKC | DEATH, STYKC | 1737 | 135 | 533 | HE609065 |
|  | Jun/AP1 | Jun, bzip1 | Jun, bzip1 | 2082 | 340 | 291 | HE609066 |
|  | *Me1*-LBP | BPI1 + BPI2 | TM, BPI1 + BPI2 | 1798 | 100 | 510 | HE609067 |
|  | *Me2*-LBP | BPI1 + BPI2 | BPI1 + BPI2 | 1765 | 87 | 500 | HE609068 |
|  | *Me3*-LBP | BPI1 + BPI2 | TM, BPI1 + BPI2 | 2193 | 29 | 485 | HE609069 |
|  | *Me4*-LBP | BPI1 + BPI2 | BPI | 879 | 7 | 248 | HE609070 |
|  | Myd88 | TIR, DEATH | Tir, DEATH | 1856 | 400 | 338 | HE609071 |
|  | p38 MAPK | STKc or PKc-like | STKc-p38 | 1738 | 21 | 278 | HE609072 |
|  | SARM | SARM, TIR | TIR | 477 | 3 | 159 | HE609073 |
|  | TOLLIP | C2, CUE | C2, CUE | 1196 | 27 | 282 | HE609074 |
|  | TRAF6 | RING, zf-TRAF, MATH | Ring, 2x TRAF z, MATH | 2070 | 99 | 596 | HE609075 |
|  | TRAM (TICAM 2) | TIR | TIR with e-value below threshold (6.03E+01) | 1702 | 25 | 568 | HE609076 |
|  | FADD | DED, DEATH | DED, DEATH | 1210 | 24 | 234 | HE609077 |
|  | MDA-5 | CARD, DEXDc, Helixc | CARD, DEXDc, Helixc | 2295 | 42 | 744 | HE609078 |
|  | DDX58 (RIG-I) | CARD, DEXDc, Helixc, RIG1-C-RD | DEXDc, Helixc, RIG1-C-RD | 1942 | 27 | 636 | HE609079 |
|  | *Me1*-PGRP | PGRP/AMI2 | TM, PGRP/AMI2 | 852 | 65 | 251 | HE609080 |
|  | *Me2*-PGRP | PGRP/AMI2 | PGRP/AMI2 | 1478 | 79 | 373 | HE609081 |
|  | *Me3-*PGRP | PGRP/AMI2 | PGRP/AMI2 | 1152 | 96 | 343 | HE609082 |
|  | *Me4*-PGRP | PGRP/AMI2 | Lys | 598 | 9 | 167 | HE609083 |
|  | *Me5*-PGRP | PGRP/AMI2 | PGRP/AMI2 | 619 | 6 | 104 | HE609084 |
|  | *Me6-*PGRP | PGRP/AMI2 | SH3b, PGRP/AMI2 | 956 | 180 | 262 | HE609085 |
|  | *Me7-*PGRP | PGRP/AMI2 | PGRP/AMI2 | 296 | 3 | 85 | HE609086 |
|  | *Me8*-PGRP | PGRP/AMI2 | PGRP/AMI2 | 773 | 51 | 200 | HE609087 |
|  | *Me1*-GNBP | Glyco_hydrolase_16 | Glyco_hydrolase_16 | 606 | 7 | 172 | HE609088 |
|  | *Me2*-GNBP | Glyco_hydrolase_16 | Transmembrane, Glyco_hydrolase_16 | 2222 | 75 | 662 | HE609089 |
|  | *Me3*-GNBP | Glyco_hydrolase_16 | Glyco_hydrolase_16 | 1687 | 2789 | 547 | HE609090 |
|  | TBK1 | PKc, STKc, rad50 | STKc | 1190 | 7 | 132 | HE609091 |
|  | TAK1 | TyrKc, MD1, Pkc | PP2Cc | 926 | 10 | 309 | HE609092 |
|  | A20 | OTU, ZnF | OTU, ZnF | 3179 | 41 | 850 | HE609093 |
|  | JAK 2 | B41, SH2, PKc (TyrKc) | PKc (TyrKc) | 1815 | 16 | 456 | HE609094 |
|  | *Me1*-STAT | STAT-alpha, STAT_int, STAT_bind, SH2 | STAT alpha, STAT bind, SH2 | 1524 | 21 | 301 & 165 | HE609095 |
|  | *Me2-*STAT | STAT-alpha, STAT_int, STAT_bind, SH2 | STAT int | 1004 | 7 | 262 | HE609096 |
|  | *Me3*-STAT | STAT-alpha, STAT_int, STAT_bind, SH2 | SH2 | 1282 | 10 | 210 | HE609097 |
|  | *Me1-*SOCS | SOCS, SH2 | SOCS, SH2 | 1830 | 182 | 250 | HE609098 |
|  | *Me2*-SOCS | SOCS, SH2 | SOCS, SH2 | 2819 | 46 | 513 | HE609099 |
| Cytokines | IL-16 | PDZ | PDZ | 850 | 8 | 165 | HE609100 |
|  | *Me1*-IL-17 | IL17 | IL17 | 2246 | 45 | 228 | HE609101 |
|  | *Me2*-IL-17 | IL17 | IL17 | 1498 | 272 | 194 | HE609102 |
|  | *Me1-*MIF | MIF | MIF | 678 | 29 | 128 | HE609103 |
|  | *Me2-*MIF | MIF | MIF | 717 | 43 | 127 | HE609104 |
|  | *Me3-*MIF | MIF | MIF | 742 | 65 | 115 | HE609105 |
|  | *Me4-*MIF | MIF | MIF | 583 | 105 | 111 | HE609106 |
|  | *Me5-*MIF | MIF | MIF | 557 | 34 | 124 | HE609107 |
| Complement system | *Me1-*C3 | A2M, A2M_recept, Isopren_C2_like, ANATO, Netrin C-terminal Domain, | A2M complement | 980 | 8 | 327 | HE609108 |
|  | *Me2*-C3 | A2M, A2M_recept, Isopren_C2_like, ANATO, Netrin C-terminal Domain, | A2M receptor, C345C | 1160 | 33 | 338 | HE609109 |
|  | C6 | FIMAC, CCP, LDLa, TSP_1, MACPF | TSP_1 | 400 | 2 | 134 | HE609110 |
|  | DMBT1 | CUB, SRCR, Zona_pellucida | 6xSRCR, VW, Zona_pellucida | 3922 | 198 | 1230 | HE609111 |
|  | ATG3 | autophagy - N, autophagy -act-C, autophagy - Cterm | autophagy - N, autophagy -act-C, autophagy - Cterm | 1178 | 26 | 346 | HE609112 |
|  | *Me1-*ATG4B | Peptidase_C54 | Peptidase_C54 | 996 | 8 | 87 | HE609113 |
|  | *Me2-*ATG4B | Peptidase_C54 | Peptidase_C54 | 707 | 9 | 160 | HE609114 |
|  | ATG4C | Peptidase_C54 | Peptidase_C54 | 1226 | 11 | 408 | HE609115 |
|  | ATG5 | ATG5 | ATG5 | 806 | 6 | 132 | HE609116 |
|  | ATG7 | MoeB, Thif1 and others. | Thif1 | 2190 | 51 | 374 | HE609117 |
| Autophagy / Apoptosis | ATG 8 (LC3) |  | MAP1_LC3 | 1755 | 34 | 120 | HE609118 |
|  | ATG9 | APG9 | APG9 | 729 | 4 | 165 | HE609119 |
|  | ATG12 | ATG12 | ATG12 | 1089 | 63 | 131 | HE609120 |
|  | ATG16 | ATG16, WD40 | ATG16 | 695 | 7 | 232 | HE609121 |
|  | Beclin1 | APG6 | APG6 | 1451 | 51 | 440 | HE609122 |
|  | mTOR | FAT, TEL1, FATC, Rapamycin bind, PI3Kc | none | 581 | 2 | 138 | HE609123 |
|  | AIFM1 | Pyr-redox | none | 1743 | 33 | 281 | HE609124 |
|  | AIFM3 (AFL) | Pyr-redox, Rieske | Pyr-redox, Rieske | 3454 | 189 | 922 | HE609125 |
|  | Bak | BCL2,TM | no | 332 | 2 | 103 | HE609126 |
|  | Bax | BCL2, TM | BCL2, TM | 1216 | 33 | 209 | HE609127 |
|  | *Me1*-BCL2 | BCL2, BHx, TM | BCL2 | 803 | 5 | 74 | HE609128 |
|  | *Me2-*BCL2 | BCL2, BHx, TM | BCL2 | 1506 | 525 | 230 | HE609129 |
|  | BCLxL | BCL2, BHx, TM | BCL2 | 1522 | 13 | 185 | HE609130 |
|  | initiator caspase with CARD domain | CASc, CARD, DED | CASc, CARD | 1485 | 43 | 339 | HE609131 |

**B:**

Homo sapiens & Mus musculus

|  |  |  | Homo sapiens |  |  |  | Mus musculus |  |  |  |
| --- | --- | --- | --- | --- | --- | --- | --- | --- | --- | --- |
| Category | Gene | *M. edulis*  Accession | Accession | Description | Query coverage | E-value | Accession | Description | Query coverage | E-value |
| Transcription factors | *Me1-*IRF | HE609042 | [NM_002198.2](http://www.ncbi.nlm.nih.gov/nucleotide/196049386?report=genbank&log$=nucltop&blast_rank=1&RID=3SVXRNM001R) | IRF 1 | 16% | 3,00E-30 | [NM_008390.2](http://www.ncbi.nlm.nih.gov/nucleotide/226874836?report=genbank&log$=nucltop&blast_rank=1&RID=3SVYGN4501P) | IRF 1 | 17% | 9,00E-31 |
|  | *Me2-*IRF | HE609043 | [NM_002199.3](http://www.ncbi.nlm.nih.gov/nucleotide/153082751?report=genbank&log$=nucltop&blast_rank=1&RID=3SWC6WED011) | IRF 2 | 49% | 6,00E-36 | [BC006577.1](http://www.ncbi.nlm.nih.gov/nucleotide/13879219?report=genbank&log$=nucltop&blast_rank=1&RID=3SWDB46S011) | IRF 2 | 49% | 3,00E-36 |
|  | *Me3-*IRF | HE609044 | [NM_002199.3](http://www.ncbi.nlm.nih.gov/nucleotide/153082751?report=genbank&log$=nucltop&blast_rank=1&RID=3SX0P772016) | IRF 2 | 19% | 3,00E-36 | [BC110666.1](http://www.ncbi.nlm.nih.gov/nucleotide/83404938?report=genbank&log$=nucltop&blast_rank=1&RID=3SX23ZE8011) | IRF 2 | 23% | 4,00E-37 |
|  | *Me4-*IRF | HE609045 | [NM_002199.3](http://www.ncbi.nlm.nih.gov/nucleotide/153082751?report=genbank&log$=nucltop&blast_rank=1&RID=3SXTKWZF016) | IRF 2 | 53% | 1,00E-31 | [BC006577.1](http://www.ncbi.nlm.nih.gov/nucleotide/13879219?report=genbank&log$=nucltop&blast_rank=2&RID=3SXU5RSG01R) | IRF 2 | 53% | 8,00E-32 |
|  | *Me5-*IRF | HE609046 | [NM_002199.3](http://www.ncbi.nlm.nih.gov/nucleotide/153082751?report=genbank&log$=nucltop&blast_rank=1&RID=3SYDN39U01R) | IRF 2 | 58% | 8,00E-37 | [BC110666.1](http://www.ncbi.nlm.nih.gov/nucleotide/83404938?report=genbank&log$=nucltop&blast_rank=1&RID=3SYEEH7Z016) | IRF 2 | 58% | 2,00E-37 |
|  | LITAF | HE609047 | [BC101402.1](http://www.ncbi.nlm.nih.gov/nucleotide/72533691?report=genbank&log$=nucltop&blast_rank=8&RID=FFC4PJV0011) | LITAF | 17% | 3,00E-21 | [NM_019980.2](http://www.ncbi.nlm.nih.gov/nucleotide/226693372?report=genbank&log$=nucltop&blast_rank=1&RID=FFC5CJBZ016) | LITAF | 17% | 2,00E-22 |
|  | *NF-κB* p65 | HE609048 | [M62399.1](http://www.ncbi.nlm.nih.gov/nucleotide/189503?report=genbank&log$=nucltop&blast_rank=7&RID=3PM482N5014) | *NF-κB* p65 | 24% | 2,00E-76 | [NM_009044.2](http://www.ncbi.nlm.nih.gov/nucleotide/112181203?report=genbank&log$=nucltop&blast_rank=1&RID=3PMZT8XU01P) | Rel | 19% | 2,00E-74 |
|  | *Me1*- *NF-κB* 1 p50 | HE609049 | [BC033210.1](http://www.ncbi.nlm.nih.gov/nucleotide/21620131?report=genbank&log$=nucltop&blast_rank=1&RID=3SNXFD9Z011) | *NF-κB* 1 p50 | 32% | 3,00E-59 | [M57999.1](http://www.ncbi.nlm.nih.gov/nucleotide/201931?report=genbank&log$=nucltop&blast_rank=1&RID=3SNYEGCD011) | *NF-κB* | 33% | 4,00E-59 |
|  | *Me2*- *NF-κB* 1 p50 | HE609050 | [M55643.1](http://www.ncbi.nlm.nih.gov/nucleotide/189179?report=genbank&log$=nucltop&blast_rank=4&RID=A478SBBG01S) | *NF-κB* 1 | 47% | 1,00E-89 | [AB119195.1](http://www.ncbi.nlm.nih.gov/nucleotide/34447178?report=genbank&log$=nucltop&blast_rank=2&RID=A47AE9C2014) | *NF-κB* 1 p105 | 48% | 5,00E-90 |
| PRR pathway proteins | Caspase 3 subunit p12 | HE609051 | [NM_004346.3](http://www.ncbi.nlm.nih.gov/nucleotide/73622121?report=genbank&log$=nucltop&blast_rank=1&RID=3V6516UM01R) | caspase 3 | 18% | 2,00E-33 | [Y13088.1](http://www.ncbi.nlm.nih.gov/nucleotide/2094813?report=genbank&log$=nucltop&blast_rank=1&RID=3V5UWDHP01P) | caspase 7 | 18% | 7,00E-33 |
|  | *Me1-*TNFR | HE609052 | n |  |  |  | n |  |  |  |
|  | *Me2-*TNFR | HE609053 | [BC047321.1](http://www.ncbi.nlm.nih.gov/nucleotide/28704106?report=genbank&log$=nucltop&blast_rank=5&RID=3Y4HP69P011) | Tnfrsf19 | 14% | 4,00E-04 | [NM_001164155.1](http://www.ncbi.nlm.nih.gov/nucleotide/256221105?report=genbank&log$=nucltop&blast_rank=1&RID=3Y4JD09N016) | Tnfrsf19 | 14% | 1,00E-04 |
|  | *Me3*-TNFR | HE609054 | n |  |  |  | [NM_013869.5](http://www.ncbi.nlm.nih.gov/nucleotide/256221051?report=genbank&log$=nucltop&blast_rank=1&RID=3Y568BP2011) | Tnfrsf19 | 11% | 2,00E-04 |
|  | *Me1*-TRAF | HE609055 | [U15637.1](http://www.ncbi.nlm.nih.gov/nucleotide/595910?report=genbank&log$=nucltop&blast_rank=1&RID=3YAMPN3Z01P) | CD40 binding protein | 62% | 7,00E-34 | [NM_011632.2](http://www.ncbi.nlm.nih.gov/nucleotide/114842404?report=genbank&log$=nucltop&blast_rank=1&RID=3YABT60801P) | Traf3 | 61% | 3,00E-31 |
|  | *Me2*-TRAF | HE609056 | [BC001769.1](http://www.ncbi.nlm.nih.gov/nucleotide/12804686?report=genbank&log$=nucltop&blast_rank=1&RID=3YDXBC16014) | Traf4 | 93% | 1,00E-72 | [NM_009423.4](http://www.ncbi.nlm.nih.gov/nucleotide/254028256?report=genbank&log$=nucltop&blast_rank=2&RID=3YDY2F45014) | Traf4 | 92% | 2,00E-72 |
|  | *Me3*-TRAF | HE609057 | [NM_145725.1](http://www.ncbi.nlm.nih.gov/nucleotide/22027617?report=genbank&log$=nucltop&blast_rank=2&RID=3YB5RJ08014) | Traf3 | 61% | 1,00E-23 | [NM_011632.2](http://www.ncbi.nlm.nih.gov/nucleotide/114842404?report=genbank&log$=nucltop&blast_rank=3&RID=3YB6E478016) | Traf3 | 61% | 1,00E-23 |
|  | *Me4*-TRAF | HE609058 | [NM_004295.3](http://www.ncbi.nlm.nih.gov/nucleotide/118402591?report=genbank&log$=nucltop&blast_rank=6&RID=3YCSC30T016) | Traf4 | 85% | 6,00E-40 | [NM_009423.4](http://www.ncbi.nlm.nih.gov/nucleotide/254028256?report=genbank&log$=nucltop&blast_rank=1&RID=3YCT3BS201P) | Traf4 | 85% | 3,00E-40 |
|  | *Me1*-I*κ*B | HE609059 | [NM_004556.2](http://www.ncbi.nlm.nih.gov/nucleotide/71274108?report=genbank&log$=nucltop&blast_rank=1&RID=3PG56CJP01R) | I*κ*B epsilon | 21% | 5,00E-19 | [AF030896.1](http://www.ncbi.nlm.nih.gov/nucleotide/2739157?report=genbank&log$=nucltop&blast_rank=1&RID=3PG6804W01P) | I*κ*B epsilon | 18% | 8,00E-22 |
|  | *Me2*-I*κ*B | HE609060 | [NM_003998.3](http://www.ncbi.nlm.nih.gov/nucleotide/259155300?report=genbank&log$=nucltop&blast_rank=1&RID=3PGZYNTH01P) | *NF-κB* 1 | 35% | 4,00E-31 | [NM_008689.2](http://www.ncbi.nlm.nih.gov/nucleotide/117606363?report=genbank&log$=nucltop&blast_rank=1&RID=3PH11P2Z01R) | *NF-κB* 1 | 34% | 3,00E-30 |
|  | IKK | HE609061 | [AF080157.1](http://www.ncbi.nlm.nih.gov/nucleotide/4185272?report=genbank&log$=nucltop&blast_rank=2&RID=3PJ6YVP2016) | IKK | 73% | 1,00E-37 | [NM_007700.2](http://www.ncbi.nlm.nih.gov/nucleotide/242332487?report=genbank&log$=nucltop&blast_rank=1&RID=3PJ7WZHX01P) | Chuk | 66% | 3,00E-38 |
|  | *Me1*-caspase 8 | HE609062 | [NM_032977.3](http://www.ncbi.nlm.nih.gov/nucleotide/98985798?report=genbank&log$=nucltop&blast_rank=1&RID=3V30STT2016) | casp 10 | 26% | 2,00E-10 | [NM_009812.2](http://www.ncbi.nlm.nih.gov/nucleotide/110626095?report=genbank&log$=nucltop&blast_rank=2&RID=3V31UPPB011) | casp 8 | 32% | 1,00E-12 |
|  | *Me2*-caspase 8 | HE609063 | [NM_001080125.1](http://www.ncbi.nlm.nih.gov/nucleotide/122056475?report=genbank&log$=nucltop&blast_rank=1&RID=3V4NZGDP01R) | casp 8 | 93% | 5,00E-30 | [NM_001080126.1](http://www.ncbi.nlm.nih.gov/nucleotide/122056577?report=genbank&log$=nucltop&blast_rank=2&RID=3V4UV52Z011) | casp 8 | 99% | 2,00E-26 |
|  | ECSIT | HE609064 | [NM_016581.3](http://www.ncbi.nlm.nih.gov/nucleotide/216548522?report=genbank&log$=nucltop&blast_rank=1&RID=62E8EEV601S) | ECSIT homolog | 50% | 2,00E-49 | [NM_012029.1](http://www.ncbi.nlm.nih.gov/nucleotide/6755521?report=genbank&log$=nucltop&blast_rank=1&RID=62E9J2NN016) | ECSIT homolog | 50% | 7,00E-49 |
|  | IRAK 4 | HE609065 | [AF445802.1](http://www.ncbi.nlm.nih.gov/nucleotide/20219009?report=genbank&log$=nucltop&blast_rank=3&RID=3T47WUBS01P) | IRAK 4 | 54% | 5,00E-58 | [AF445803.1](http://www.ncbi.nlm.nih.gov/nucleotide/20219011?report=genbank&log$=nucltop&blast_rank=1&RID=3T4KHBFR01R) | IRAK 4 | 54% | 9,00E-55 |
|  | Jun/AP1 | HE609066 | [BT019759.1](http://www.ncbi.nlm.nih.gov/nucleotide/54696383?report=genbank&log$=nucltop&blast_rank=1&RID=3V1V0WTG014) | v-jun sarcoma virus 17 oncogene homolog (avian) | 26% | 2,00E-36 | [X15358.1](http://www.ncbi.nlm.nih.gov/nucleotide/52765?report=genbank&log$=nucltop&blast_rank=1&RID=3V1VR5U9016) | junD proto-oncogene | 25% | 4,00E-38 |
|  | *Me1*-LBP | HE609067 | [AF105067.1](http://www.ncbi.nlm.nih.gov/nucleotide/4530276?report=genbank&log$=nucltop&blast_rank=1&RID=3NVYW1UG01P) | LBP | 64% | 8,00E-46 | [NM_008489.2](http://www.ncbi.nlm.nih.gov/nucleotide/113865990?report=genbank&log$=nucltop&blast_rank=1&RID=3NX7V2A101R) | LBP | 62% | 5,00E-42 |
|  | *Me2*-LBP | HE609068 | [AF105067.1](http://www.ncbi.nlm.nih.gov/nucleotide/4530276?report=genbank&log$=nucltop&blast_rank=1&RID=3NYJD2FD01R) | LBP | 66% | 1,00E-56 | [NM_008489.2](http://www.ncbi.nlm.nih.gov/nucleotide/113865990?report=genbank&log$=nucltop&blast_rank=1&RID=3PAJ8C3G014) | LBP | 63% | 2,00E-48 |
|  | *Me3*-LBP | HE609069 | [AF105067.1](http://www.ncbi.nlm.nih.gov/nucleotide/4530276?report=genbank&log$=nucltop&blast_rank=1&RID=3P95CM48014) | LBP | 25% | 4,00E-20 | [NM_008489.2](http://www.ncbi.nlm.nih.gov/nucleotide/113865990?report=genbank&log$=nucltop&blast_rank=2&RID=3P96ST7M01P) | LBP | 26% | 1,00E-22 |
|  | *Me4*-LBP | HE609070 | [AK313625.1](http://www.ncbi.nlm.nih.gov/nucleotide/164694814?report=genbank&log$=nucltop&blast_rank=1&RID=3PAUTGYZ016) | LBP | 45% | 1,00E-14 | [BC004795.1](http://www.ncbi.nlm.nih.gov/nucleotide/13435902?report=genbank&log$=nucltop&blast_rank=2&RID=3PAVXR0S014) | LBP | 64% | 3,00E-14 |
|  | Myd88 | HE609071 | [AB446470.1](http://www.ncbi.nlm.nih.gov/nucleotide/194239490?report=genbank&log$=nucltop&blast_rank=2&RID=3PCG0K6X01R) | Myd88 | 28% | 2,00E-25 | [BC058787.1](http://www.ncbi.nlm.nih.gov/nucleotide/37589151?report=genbank&log$=nucltop&blast_rank=5&RID=3PCGX2KN01P) | Myd88 | 24% | 4,00E-25 |
|  | p38 MAPK | HE609072 | [L35253.1](http://www.ncbi.nlm.nih.gov/nucleotide/529039?report=genbank&log$=nucltop&blast_rank=4&RID=62KTWP3J011) | p38 MAPK | 53% | 6,00E-141 | [NM_001168514.1](http://www.ncbi.nlm.nih.gov/nucleotide/270341385?report=genbank&log$=nucltop&blast_rank=1&RID=62M6499D013) | Mapk14 | 53% | 5,00E-141 |
|  | SARM | HE609073 | [NM_015077.2](http://www.ncbi.nlm.nih.gov/nucleotide/154090975?report=genbank&log$=nucltop&blast_rank=2&RID=5K8D8PYV012) | SARM | 83% | 2,00E-50 | [NM_172795.3](http://www.ncbi.nlm.nih.gov/nucleotide/281188263?report=genbank&log$=nucltop&blast_rank=1&RID=5K8EBXGK01N) | SARM | 83% | 2,00E-49 |
|  | TOLLIP | HE609074 | AK315592.1 | TOLLIP | 53% | 3,00E-72 | [AJ242971.1](http://www.ncbi.nlm.nih.gov/nucleotide/6048271?report=genbank&log$=nucltop&blast_rank=1&RID=3SUDHCY001P) | TOLLIP | 53% | 1,00E-72 |
|  | TRAF6 | HE609075 | [U78798.1](http://www.ncbi.nlm.nih.gov/nucleotide/1732425?report=genbank&log$=nucltop&blast_rank=6&RID=3PEJHJEU01P) | Traf6 | 57% | 5,00E-85 | [D84655.1](http://www.ncbi.nlm.nih.gov/nucleotide/1651194?report=genbank&log$=nucltop&blast_rank=2&RID=3PEKE3SK01R) | Traf6 | 55% | 5,00E-84 |
|  | TRAM (TICAM 2) | HE609076 | [NM_021649.6](http://www.ncbi.nlm.nih.gov/nucleotide/311307323?report=genbank&log$=nucltop&blast_rank=4&RID=NR8PT3Y2012) | TICAM 2 | 13% | 0.13 | [NM_173394.3](http://www.ncbi.nlm.nih.gov/nucleotide/288541414?report=genbank&log$=nucltop&blast_rank=4&RID=NR8R8XJ101R) | TICAM 2 | 13% | 0.075 |
|  | FADD | HE609077 | [BT006927.1](http://www.ncbi.nlm.nih.gov/nucleotide/30582692?report=genbank&log$=nucltop&blast_rank=1&RID=3VN9WGX7016) | FADD | 36% | 9,00E-20 | [U43184.1](http://www.ncbi.nlm.nih.gov/nucleotide/1167558?report=genbank&log$=nucltop&blast_rank=1&RID=3VNAR1JA011) | FADD | 37% | 4,00E-19 |
|  | MDA-5 | HE609078 | [AF095844.1](http://www.ncbi.nlm.nih.gov/nucleotide/11344593?report=genbank&log$=nucltop&blast_rank=2&RID=3UXAE9JC016) | MDA-5 | 34% | 3,00E-43 | [AY075132.1](http://www.ncbi.nlm.nih.gov/nucleotide/18698980?report=genbank&log$=nucltop&blast_rank=1&RID=3UXB4CZ7011) | HELICARD | 32% | 1,00E-42 |
|  | DDX58 (RIG-I) | HE609079 | [AF038963.1](http://www.ncbi.nlm.nih.gov/nucleotide/4405794?report=genbank&log$=nucltop&blast_rank=3&RID=89RT08P7013) | Rig1 | 54% | 1,00E-49 | [AY553221.1](http://www.ncbi.nlm.nih.gov/nucleotide/45385111?report=genbank&log$=nucltop&blast_rank=5&RID=89T7JAET013) | Rig1 | 52% | 4,00E-50 |
|  | *Me1*-PGRP | HE609080 | [BC107158.1](http://www.ncbi.nlm.nih.gov/nucleotide/78070689?report=genbank&log$=nucltop&blast_rank=2&RID=3VF9J10X01P) | PGRP | 55% | 6,00E-36 | [BC120840.1](http://www.ncbi.nlm.nih.gov/nucleotide/111308391?report=genbank&log$=nucltop&blast_rank=2&RID=3VFAGDV9011) | PGRP 4 | 57% | 2,00E-39 |
|  | *Me2*-PGRP | HE609081 | [BC128114.1](http://www.ncbi.nlm.nih.gov/nucleotide/118763939?report=genbank&log$=nucltop&blast_rank=1&RID=3VFK28R2011) | PGRP 3 | 36% | 2,00E-40 | [BC128291.1](http://www.ncbi.nlm.nih.gov/nucleotide/118764226?report=genbank&log$=nucltop&blast_rank=1&RID=3VFKU0RT011) | PGRP 3 | 33% | 1,00E-43 |
|  | *Me3-*PGRP | HE609082 | [NM_005091.2](http://www.ncbi.nlm.nih.gov/nucleotide/291490703?report=genbank&log$=nucltop&blast_rank=1&RID=3VFYAVSV011) | PGRP 1 | 35% | 3,00E-14 | [BC128291.1](http://www.ncbi.nlm.nih.gov/nucleotide/118764226?report=genbank&log$=nucltop&blast_rank=1&RID=3VFZ68C501P) | PGRP 3 | 30% | 9,00E-12 |
|  | *Me4*-PGRP | HE609083 | [NM_153374.2](http://www.ncbi.nlm.nih.gov/nucleotide/221139696?report=genbank&log$=nucltop&blast_rank=1&RID=3VHJFJSV011) | LYSMD2 | 25% | 6,00E-12 | [NM_027309.2](http://www.ncbi.nlm.nih.gov/nucleotide/130490960?report=genbank&log$=nucltop&blast_rank=1&RID=3VHK4D1G016) | LYSMD2 | 25% | 3,00E-12 |
|  | *Me5*-PGRP | HE609084 | [BC142636.1](http://www.ncbi.nlm.nih.gov/nucleotide/148744334?report=genbank&log$=nucltop&blast_rank=2&RID=3VH22N0X011) | PGRP 4 | 54% | 3,00E-28 | [NM_207247.4](http://www.ncbi.nlm.nih.gov/nucleotide/260166615?report=genbank&log$=nucltop&blast_rank=1&RID=3VH2VZ9B014) | PGRP 3 | 49% | 1,00E-30 |
|  | *Me6-*PGRP | HE609085 | [NM_052891.1](http://www.ncbi.nlm.nih.gov/nucleotide/16418404?report=genbank&log$=nucltop&blast_rank=1&RID=3VHVGEKN016) | PGRP 3 | 50% | 1,00E-41 | [BC128291.1](http://www.ncbi.nlm.nih.gov/nucleotide/118764226?report=genbank&log$=nucltop&blast_rank=1&RID=3VHW5SP601R) | PGRP 3 | 49% | 2,00E-43 |
|  | *Me7-*PGRP | HE609086 | [NM_005091.2](http://www.ncbi.nlm.nih.gov/nucleotide/291490703?report=genbank&log$=nucltop&blast_rank=1&RID=3VJXN7RW011) | PGRP 1 | 65% | 9,00E-13 | [NM_009402.2](http://www.ncbi.nlm.nih.gov/nucleotide/118130401?report=genbank&log$=nucltop&blast_rank=2&RID=3VJYPKGX016) | PGRP 1 | 63% | 8,00E-09 |
|  | *Me8*-PGRP | HE609087 | [NM_005091.2](http://www.ncbi.nlm.nih.gov/nucleotide/291490703?report=genbank&log$=nucltop&blast_rank=1&RID=3VKBEACH011) | PGRP 1 | 62% | 4,00E-45 | [NM_009402.2](http://www.ncbi.nlm.nih.gov/nucleotide/118130401?report=genbank&log$=nucltop&blast_rank=2&RID=3VKC8BMJ011) | PGRP 1 | 65% | 2,00E-44 |
|  | *Me1*-GNBP | HE609088 | n |  |  |  | n |  |  |  |
|  | *Me2*-GNBP | HE609089 | n |  |  |  | n |  |  |  |
|  | *Me3*-GNBP | HE609090 | n |  |  |  | n |  |  |  |
|  | TBK1 | HE609091 | [NM_013254.2](http://www.ncbi.nlm.nih.gov/nucleotide/19743810?report=genbank&log$=nucltop&blast_rank=2&RID=40JBJJSC011) | TBK1 | 45% | 1,00E-43 | [NM_019786.4](http://www.ncbi.nlm.nih.gov/nucleotide/251823838?report=genbank&log$=nucltop&blast_rank=1&RID=40JCVC1B011) | TBK1 | 45% | 2,00E-43 |
|  | TAK1 | HE609092 | [AB209372.1](http://www.ncbi.nlm.nih.gov/nucleotide/62088323?report=genbank&log$=nucltop&blast_rank=1&RID=40KRG03Z01R) | TAK1 | 61% | 6,00E-50 | [BC041110.1](http://www.ncbi.nlm.nih.gov/nucleotide/27552842?report=genbank&log$=nucltop&blast_rank=2&RID=40KSYZ1501P) | TAK1 | 67% | 5,00E-49 |
|  | A20 | HE609093 | [BC114480.1](http://www.ncbi.nlm.nih.gov/nucleotide/89365978?report=genbank&log$=nucltop&blast_rank=6&RID=40UXM8S0011) | TNF, alpha-induced protein 3 | 31% | 4,00E-42 | [U19463.1](http://www.ncbi.nlm.nih.gov/nucleotide/640036?report=genbank&log$=nucltop&blast_rank=1&RID=40UPBK1H01R) | A20 | 28% | 9,00E-43 |
|  | JAK 2 | HE609094 | [AF001362.1](http://www.ncbi.nlm.nih.gov/nucleotide/3236321?report=genbank&log$=nucltop&blast_rank=2&RID=40AF0UUH014) | JAK 2 | 41% | 5,00E-43 | [BC059834.1](http://www.ncbi.nlm.nih.gov/nucleotide/37590525?report=genbank&log$=nucltop&blast_rank=1&RID=40AFK9RE014) | JAK 2 | 41% | 4,00E-42 |
|  | *Me1*-STAT | HE609095 | [U48730.2](http://www.ncbi.nlm.nih.gov/nucleotide/6981725?report=genbank&log$=nucltop&blast_rank=3&RID=40B9HNFK011) | STAT 5b | 76% | 3,00E-58 | [Z48539.1](http://www.ncbi.nlm.nih.gov/nucleotide/758635?report=genbank&log$=nucltop&blast_rank=1&RID=40BA6H8H016) | STAT 5b | 71% | 2,00E-58 |
|  | *Me2-*STAT | HE609096 | [NM_003152.3](http://www.ncbi.nlm.nih.gov/nucleotide/221316717?report=genbank&log$=nucltop&blast_rank=4&RID=40DU69CS01P) | STAT 5a | 42% | 3,00E-14 | [NM_011488.3](http://www.ncbi.nlm.nih.gov/nucleotide/255759966?report=genbank&log$=nucltop&blast_rank=2&RID=40DUXRFH01P) | STAT 5a | 44% | 2,00E-21 |
|  | *Me3*-STAT | HE609097 | [NM_003152.3](http://www.ncbi.nlm.nih.gov/nucleotide/221316717?report=genbank&log$=nucltop&blast_rank=2&RID=40DZYRK4011) | STAT 5a | 21% | 7,00E-26 | [NM_011488.3](http://www.ncbi.nlm.nih.gov/nucleotide/255759966?report=genbank&log$=nucltop&blast_rank=1&RID=40E0MKCK016) | STAT 5a | 21% | 8,00E-25 |
|  | *Me1-*SOCS | HE609098 | [AF020590.1](http://www.ncbi.nlm.nih.gov/nucleotide/4090853?report=genbank&log$=nucltop&blast_rank=1&RID=40FMEDRF011) | SOCS 2 | 19% | 7,00E-24 | [NM_007706.4](http://www.ncbi.nlm.nih.gov/nucleotide/274325752?report=genbank&log$=nucltop&blast_rank=1&RID=40FNAZJH01P) | SOCS 2 | 18% | 1,00E-22 |
|  | *Me2*-SOCS | HE609099 | [NM_144949.2](http://www.ncbi.nlm.nih.gov/nucleotide/41349445?report=genbank&log$=nucltop&blast_rank=8&RID=40HA9AAK011) | SOCS 5 | 21% | 6,00E-83 | [NM_019654.2](http://www.ncbi.nlm.nih.gov/nucleotide/34328248?report=genbank&log$=nucltop&blast_rank=1&RID=40HB5ZJM016) | SOCS 5 | 21% | 7,00E-83 |
| Cytokines | IL-16 | HE609100 | [NM_001172128.1](http://www.ncbi.nlm.nih.gov/nucleotide/289063394?report=genbank&log$=nucltop&blast_rank=1&RID=3UTZZP3F011) | IL 16 | 36% | 3,00E-15 | [NM_001081064.1](http://www.ncbi.nlm.nih.gov/nucleotide/124486715?report=genbank&log$=nucltop&blast_rank=1&RID=3UU0TXG001R) | PDZ domain containing 2 | 32% | 2,00E-13 |
|  | *Me1*-IL-17 | HE609101* | Q6NZ94 | IL17A | 35% | 1.6 | n |  |  |  |
|  | *Me2*-IL-17 | HE609102 | n | n |  |  | n |  |  |  |
|  | *Me1-*MIF | HE609103 | [BC013976.2](http://www.ncbi.nlm.nih.gov/nucleotide/33878417?report=genbank&log$=nucltop&blast_rank=2&RID=4BF09F4T014) | MIF | 51% | 4,00E-22 | [L10613.1](http://www.ncbi.nlm.nih.gov/nucleotide/402716?report=genbank&log$=nucltop&blast_rank=1&RID=4BE7RF7K016) | glycosylation-inhibiting factor | 51% | 2,00E-21 |
|  | *Me2-*MIF | HE609104 | [EF611126.1](http://www.ncbi.nlm.nih.gov/nucleotide/148608028?report=genbank&log$=nucltop&blast_rank=1&RID=4BF09F4T014) | MIF | 45% | 2,00E-06 | [NM_010798.2](http://www.ncbi.nlm.nih.gov/nucleotide/118130170?report=genbank&log$=nucltop&blast_rank=8&RID=4BE7RF7K016) | MIF | 45% | 3,00E-05 |
|  | *Me3-*MIF | HE609105 | [BC013976.2](http://www.ncbi.nlm.nih.gov/nucleotide/33878417?report=genbank&log$=nucltop&blast_rank=2&RID=4BF09F4T014) | MIF | 46% | 7,00E-22 | [NM_010798.2](http://www.ncbi.nlm.nih.gov/nucleotide/118130170?report=genbank&log$=nucltop&blast_rank=2&RID=4BE7RF7K016) | MIF | 49% | 1,00E-21 |
|  | *Me4-*MIF | HE609106 | n |  |  |  | n |  |  |  |
|  | *Me5-*MIF | HE609107 | [EF611126.1](http://www.ncbi.nlm.nih.gov/nucleotide/148608028?report=genbank&log$=nucltop&blast_rank=1&RID=4BF09F4T014) | MIF | 58% | 6,00E-10 | [NM_010798.2](http://www.ncbi.nlm.nih.gov/nucleotide/118130170?report=genbank&log$=nucltop&blast_rank=8&RID=4BE7RF7K016) | MIF | 58% | 2,00E-09 |
| Complement system | *Me1-*C3 | HE609108 | [NM_015692.2](http://www.ncbi.nlm.nih.gov/nucleotide/118600976?report=genbank&log$=nucltop&blast_rank=1&RID=40VCMG1E014) | C3 and PZP-like, alpha-2-macroglobulin domain containing 8 | 48% | 2,00E-23 | [AY083458.1](http://www.ncbi.nlm.nih.gov/nucleotide/20070079?report=genbank&log$=nucltop&blast_rank=4&RID=40VDHJ6K016) | GPI-anchored alpha-2 macroglobulin-related protein | 38% | 7,00E-18 |
|  | *Me2*-C3 | HE609109 | [J04763.1](http://www.ncbi.nlm.nih.gov/nucleotide/1162925?report=genbank&log$=nucltop&blast_rank=1&RID=48UNCVC7014) | C3 | 25% | 2,00E-11 | [NM_009778.2](http://www.ncbi.nlm.nih.gov/nucleotide/126518316?report=genbank&log$=nucltop&blast_rank=2&RID=48UP1DT2016) | C3 | 25% | 1,00E-09 |
|  | C6 | HE609110 | [J04506.1](http://www.ncbi.nlm.nih.gov/nucleotide/618465?report=genbank&log$=nucltop&blast_rank=2&RID=4XP7YNF2014) | C6 | 30% | 9,00E-06 | [AF184900.1](http://www.ncbi.nlm.nih.gov/nucleotide/6503075?report=genbank&log$=nucltop&blast_rank=2&RID=4XP9VV4F016) | C6 | 30% | 2,00E-04 |
|  | DMBT1 | HE609111 | [NM_017579.2](http://www.ncbi.nlm.nih.gov/nucleotide/148539843?report=genbank&log$=nucltop&blast_rank=1&RID=48X7ZWJ1014) | DMBT1 | 26% | 2,00E-49 | [NM_007769.2](http://www.ncbi.nlm.nih.gov/nucleotide/87239966?report=genbank&log$=nucltop&blast_rank=1&RID=48X8H66S016) | DMBT1 | 30% | 1,00E-26 |
|  | ATG3 | HE609112 | [NM_022488.3](http://www.ncbi.nlm.nih.gov/nucleotide/34147490?report=genbank&log$=nucltop&blast_rank=4&RID=4B2EG8ND01P) | ATG3 | 62% | 4,00E-127 | [BC010809.1](http://www.ncbi.nlm.nih.gov/nucleotide/14789860?report=genbank&log$=nucltop&blast_rank=5&RID=4B2F3ENK016) | ATG3 | 63% | 2,00E-128 |
|  | *Me1-*ATG4B | HE609113 | [BC000719.2](http://www.ncbi.nlm.nih.gov/nucleotide/34783065?report=genbank&log$=nucltop&blast_rank=1&RID=4B4UKW3601R) | ATG4 B | 42% | 3,00E-40 | [NM_174874.3](http://www.ncbi.nlm.nih.gov/nucleotide/146198771?report=genbank&log$=nucltop&blast_rank=1&RID=4B4VAXCC014) | ATG4 B | 42% | 2,00E-39 |
|  | *Me2-*ATG4B | HE609114 | [NM_178326.2](http://www.ncbi.nlm.nih.gov/nucleotide/47132612?report=genbank&log$=nucltop&blast_rank=2&RID=4B561YF7014) | ATG4 B | 71% | 3,00E-69 | [NM_174874.3](http://www.ncbi.nlm.nih.gov/nucleotide/146198771?report=genbank&log$=nucltop&blast_rank=2&RID=4B56J80N011) | ATG4 B | 71% | 3,00E-69 |
|  | ATG4C | HE609115 | [AJ312234.1](http://www.ncbi.nlm.nih.gov/nucleotide/27763972?report=genbank&log$=nucltop&blast_rank=3&RID=4B5SMDG3014) | ATG4 C | 66% | 7,00E-84 | [AJ312233.1](http://www.ncbi.nlm.nih.gov/nucleotide/27763970?report=genbank&log$=nucltop&blast_rank=1&RID=4B5R06H3016) | ATG4 C | 64% | 9,00E-83 |
|  | ATG5 | HE609116 | [NM_004849.2](http://www.ncbi.nlm.nih.gov/nucleotide/92859692?report=genbank&log$=nucltop&blast_rank=2&RID=4B177BFE014) | ATG5 | 66% | 2,00E-66 | [NM_053069.5](http://www.ncbi.nlm.nih.gov/nucleotide/158508499?report=genbank&log$=nucltop&blast_rank=1&RID=4B17V36V011) | ATG5 | 67% | 6,00E-67 |
|  | ATG7 | HE609117 | [NM_006395.2](http://www.ncbi.nlm.nih.gov/nucleotide/222144225?report=genbank&log$=nucltop&blast_rank=1&RID=4B3GMM2001R) | ATG7 | 55% | 2,00E-137 | [BC058597.1](http://www.ncbi.nlm.nih.gov/nucleotide/37589292?report=genbank&log$=nucltop&blast_rank=4&RID=4B3HHAU101R) | ATG7 | 55% | 4,00E-141 |
| Autophagy / Apoptosis | ATG 8 (LC3) | HE609118 | [NM_022818.4](http://www.ncbi.nlm.nih.gov/nucleotide/197382745?report=genbank&log$=nucltop&blast_rank=7&RID=4B0PD4XK014) | LC3 | 19% | 6,00E-49 | [NM_025735.2](http://www.ncbi.nlm.nih.gov/nucleotide/227499340?report=genbank&log$=nucltop&blast_rank=1&RID=4B0R2K4F016) | LC3 | 20% | 7,00E-49 |
|  | ATG9 | HE609119 | [NM_173681.5](http://www.ncbi.nlm.nih.gov/nucleotide/239582719?report=genbank&log$=nucltop&blast_rank=2&RID=4B7VTF6J011) | ATG9 | 96% | 3,00E-59 | [NM_001002897.3](http://www.ncbi.nlm.nih.gov/nucleotide/146219844?report=genbank&log$=nucltop&blast_rank=2&RID=4B7WJYBR016) | ATG9 | 96% | 4,00E-61 |
|  | ATG12 | HE609120 | [NM_004707.3](http://www.ncbi.nlm.nih.gov/nucleotide/290560745?report=genbank&log$=nucltop&blast_rank=1&RID=4B1Z99WX011) | ATG12 | 32% | 1,00E-41 | [NM_026217.3](http://www.ncbi.nlm.nih.gov/nucleotide/145966751?report=genbank&log$=nucltop&blast_rank=1&RID=4B1ZWGB1016) | ATG12 | 31% | 2,00E-40 |
|  | ATG16 | HE609121 | [NM_030803.6](http://www.ncbi.nlm.nih.gov/nucleotide/124256479?report=genbank&log$=nucltop&blast_rank=3&RID=4B6UCX2Y014) | ATG16 | 72% | 8,00E-35 | [BC049122.1](http://www.ncbi.nlm.nih.gov/nucleotide/29144976?report=genbank&log$=nucltop&blast_rank=2&RID=4B6N7N0M014) | ATG16 | 81% | 5,00E-35 |
|  | Beclin1 | HE609122 | [NM_003766.3](http://www.ncbi.nlm.nih.gov/nucleotide/187608304?report=genbank&log$=nucltop&blast_rank=2&RID=491EC4ND014) | Beclin1 | 83% | 7,00E-149 | [NM_019584.3](http://www.ncbi.nlm.nih.gov/nucleotide/142352751?report=genbank&log$=nucltop&blast_rank=2&RID=491F3GJ0016) | Beclin1 | 77% | 4,00E-142 |
|  | mTOR | HE609123 | [NM_004958.3](http://www.ncbi.nlm.nih.gov/nucleotide/206725550?report=genbank&log$=nucltop&blast_rank=6&RID=4B92CU0H014) | mTOR | 80% | 6,00E-44 | [NM_020009.2](http://www.ncbi.nlm.nih.gov/nucleotide/227330585?report=genbank&log$=nucltop&blast_rank=5&RID=4B92Z0R0011) | mTOR | 80% | 2,00E-44 |
|  | AIFM1 | HE609124 | [NM_004208.3](http://www.ncbi.nlm.nih.gov/nucleotide/296923790?report=genbank&log$=nucltop&blast_rank=1&RID=4AY0VDF5011) | AIFM1 | 69% | 5,00E-91 | [DQ016499.1](http://www.ncbi.nlm.nih.gov/nucleotide/68005615?report=genbank&log$=nucltop&blast_rank=1&RID=4AY1DX5G016) | AIF short isoform 2 | 68% | 2,00E-83 |
|  | AIFM3 (AFL) | HE609125 | [NM_001146288.1](http://www.ncbi.nlm.nih.gov/nucleotide/226437567?report=genbank&log$=nucltop&blast_rank=1&RID=4AYN049X014) | AIFM3 | 41% | 8,00E-176 | [NM_175178.3](http://www.ncbi.nlm.nih.gov/nucleotide/70778908?report=genbank&log$=nucltop&blast_rank=1&RID=4AYNME7Y014) | AIFM3 | 41% | 6,00E-175 |
|  | Bak | HE609126 | [BC110337.1](http://www.ncbi.nlm.nih.gov/nucleotide/82571457?report=genbank&log$=nucltop&blast_rank=1&RID=4AWTT7HS011) | Bak1 | 63% | 2,00E-12 | [NM_007523.2](http://www.ncbi.nlm.nih.gov/nucleotide/111955301?report=genbank&log$=nucltop&blast_rank=1&RID=4AWNT062014) | Bak1 | 58% | 3,00E-12 |
|  | Bax | HE609127 | [NM_138761.3](http://www.ncbi.nlm.nih.gov/nucleotide/163659848?report=genbank&log$=nucltop&blast_rank=1&RID=4AUX0BV301R) | Bax | 30% | 2,00E-22 | [AY095934.1](http://www.ncbi.nlm.nih.gov/nucleotide/21217732?report=genbank&log$=nucltop&blast_rank=2&RID=4AVA135F014) | Bax | 30% | 4,00E-23 |
|  | *Me1*-BCL2 | HE609128 | [EU287875.1](http://www.ncbi.nlm.nih.gov/nucleotide/161277340?report=genbank&log$=nucltop&blast_rank=1&RID=4AKY99TX01P) | BCL2 | 50% | 1,00E-33 | [BC095964.1](http://www.ncbi.nlm.nih.gov/nucleotide/66365672?report=genbank&log$=nucltop&blast_rank=2&RID=4AUJDMB2014) | BCL2 | 57% | 4,00E-33 |
|  | *Me2-*BCL2 | HE609129 | [NM_021960.3](http://www.ncbi.nlm.nih.gov/nucleotide/33519459?report=genbank&log$=nucltop&blast_rank=1&RID=4AVBFV5A016) | myeloid cell leukemia sequence 1 (Mcl1) | 17% | 4,00E-11 | [NM_008562.3](http://www.ncbi.nlm.nih.gov/nucleotide/133892763?report=genbank&log$=nucltop&blast_rank=1&RID=4AVGGRVJ014) | Mcl1 | 17% | 2,00E-11 |
|  | BCLxL | HE609130 | [Z23115.1](http://www.ncbi.nlm.nih.gov/nucleotide/510900?report=genbank&log$=nucltop&blast_rank=2&RID=4AVSBJHT01P) | Bcl-xL | 22% | 5,00E-30 | [NM_009743.4](http://www.ncbi.nlm.nih.gov/nucleotide/118129881?report=genbank&log$=nucltop&blast_rank=4&RID=4AVTZUGS014) | BCL2-like 1 | 22% | 6,00E-30 |
|  | initiator caspase with CARD domain | HE609131 | [007240.1](http://www.ncbi.nlm.nih.gov/nucleotide/30583318?report=genbank&log$=nucltop&blast_rank=1&RID=4BA4W6WS01R) | casp 2 | 32% | 5,00E-17 | [D28492.1](http://www.ncbi.nlm.nih.gov/nucleotide/3090429?report=genbank&log$=nucltop&blast_rank=1&RID=4BA5NAUC01P) | casp 2 | 32% | 2,00E-16 |

Drosophila melanogaster & Caenorhabditis elegans

|  |  |  | *Drosophila melanogaster* | |  |  | *Caenorhabditis elegans* | |  |  |
| --- | --- | --- | --- | --- | --- | --- | --- | --- | --- | --- |
| Category | Gene | *M. edulis*  Accession | Accession | Description | Query coverage | E-value | Accession | Description | Query coverage | E-value |
| Transcription factors | *Me1-*IRF | HE609042 | nd |  |  |  | nd |  |  |  |
|  | *Me2-*IRF | HE609043 | nd |  |  |  | nd |  |  |  |
|  | *Me3-*IRF | HE609044 | nd |  |  |  | nd |  |  |  |
|  | *Me4-*IRF | HE609045 | nd |  |  |  | nd |  |  |  |
|  | *Me5-*IRF | HE609046 | nd |  |  |  | nd |  |  |  |
|  | LITAF | HE609047 | nd |  |  |  | nd |  |  |  |
|  | *NF-κB* p65 | HE609048 | [NM_165218.2](http://www.ncbi.nlm.nih.gov/nucleotide/281365138?report=genbank&log$=nucltop&blast_rank=1&RID=3PM4F2DY014) | dorsal | 25% | 7,00E-89 | nd |  |  |  |
|  | *Me1*-*NF-κB* 1 p50 | HE609049 | [NM_205999.1](http://www.ncbi.nlm.nih.gov/nucleotide/45552396?report=genbank&log$=nucltop&blast_rank=1&RID=3SNXVG2Z016) | cactus | 14% | 2,00E-19 | [NM_061028.2](http://www.ncbi.nlm.nih.gov/nucleotide/212645193?report=genbank&log$=nucltop&blast_rank=1&RID=3SNY4X14014) | transient receptor potential) channel family member | 16% | 1,00E-08 |
|  | *Me2*-*NF-κB* 1 p50 | HE609050 | [M23702.1](http://www.ncbi.nlm.nih.gov/nucleotide/157282?report=genbank&log$=nucltop&blast_rank=2&RID=A479A98001S) | dorsal | 48% | 1,00E-45 | nd |  |  |  |
| PRR pathway proteins | Caspase 3 subunit p12 | HE609051 | [NM_057626.3](http://www.ncbi.nlm.nih.gov/nucleotide/24762322?report=genbank&log$=nucltop&blast_rank=1&RID=3V5UCPZ5011) | death caspase-1 | 16% | 8,00E-28 | [AF088289.1](http://www.ncbi.nlm.nih.gov/nucleotide/4063375?report=genbank&log$=nucltop&blast_rank=1&RID=3V5UMPRC01R) | caspase-related protein 2B | 10% | 2,00E-05 |
|  | *Me1-*TNFR | HE609052 | nd |  |  |  | nd |  |  |  |
|  | *Me2-*TNFR | HE609053 | nd |  |  |  | nd |  |  |  |
|  | *Me3*-TNFR | HE609054 | nd |  |  |  | nd |  |  |  |
|  | *Me1*-TRAF | HE609055 | [AF111422.1](http://www.ncbi.nlm.nih.gov/nucleotide/10716151?report=genbank&log$=nucltop&blast_rank=1&RID=3YAATY4A01P) | Traf1 | 35% | 3,00E-08 | [AF348168.1](http://www.ncbi.nlm.nih.gov/nucleotide/13650032?report=genbank&log$=nucltop&blast_rank=1&RID=3YAB28E401P) | Traf1 | 42% | 1,00E-18 |
|  | *Me2*-TRAF | HE609056 | [NM_058068.3](http://www.ncbi.nlm.nih.gov/nucleotide/24581619?report=genbank&log$=nucltop&blast_rank=5&RID=3YDXJUBK01P) | Traf4 | 88% | 2,00E-62 | [AF348168.1](http://www.ncbi.nlm.nih.gov/nucleotide/13650032?report=genbank&log$=nucltop&blast_rank=1&RID=3YDXUBSZ01R) | Traf1 | 72% | 3,00E-27 |
|  | *Me3*-TRAF | HE609057 | [NM_001103610.1](http://www.ncbi.nlm.nih.gov/nucleotide/161076685?report=genbank&log$=nucltop&blast_rank=3&RID=3YB62UEH014) | Traf4 | 46% | 5,00E-18 | [NM_067372.3](http://www.ncbi.nlm.nih.gov/nucleotide/71986183?report=genbank&log$=nucltop&blast_rank=1&RID=3YB699U3016) | Traf1 | 64% | 9,00E-41 |
|  | *Me4*-TRAF | HE609058 | [NM_078525.2](http://www.ncbi.nlm.nih.gov/nucleotide/24640526?report=genbank&log$=nucltop&blast_rank=4&RID=3YCSNKWR014) | Traf6 | 59% | 8,00E-05 | nd |  |  |  |
|  | *Me1*-I*κ*B | HE609059 | [NM_057595.3](http://www.ncbi.nlm.nih.gov/nucleotide/24584590?report=genbank&log$=nucltop&blast_rank=3&RID=3PG5NTHU01P) | cactus | 33% | 8,00E-24 | nd |  |  |  |
|  | *Me2*-I*κ*B | HE609060 | [NM_205999.1](http://www.ncbi.nlm.nih.gov/nucleotide/45552396?report=genbank&log$=nucltop&blast_rank=1&RID=3PH0BABN01P) | cactus | 32% | 7,00E-32 | nd |  |  |  |
|  | IKK | HE609061 | nd |  |  |  | nd |  |  |  |
|  | *Me1*-caspase 8 | HE609062 | [NM_057626.3](http://www.ncbi.nlm.nih.gov/nucleotide/24762322?report=genbank&log$=nucltop&blast_rank=1&RID=3V319V6U014) | death casp-1 | 34% | 3,00E-10 | [AF210702.1](http://www.ncbi.nlm.nih.gov/nucleotide/11967320?report=genbank&log$=nucltop&blast_rank=2&RID=3V31JPNZ014) | casp CED-3 | 32% | 4,00E-08 |
|  | *Me2*-caspase 8 | HE609063 | [AF222007.1](http://www.ncbi.nlm.nih.gov/nucleotide/7264719?report=genbank&log$=nucltop&blast_rank=1&RID=3V4PBUAC01R) | casp 6-like protein BG2 | 73% | 4,00E-17 | [AF210702.1](http://www.ncbi.nlm.nih.gov/nucleotide/11967320?report=genbank&log$=nucltop&blast_rank=1&RID=3V4PJU4G011) | casp CED-3 | 60% | 2,00E-08 |
|  | ECSIT | HE609064 | [NM_141345.2](http://www.ncbi.nlm.nih.gov/nucleotide/24644457?report=genbank&log$=nucltop&blast_rank=4&RID=62E8TYXH011) | ECSIT | 46% | 3,00E-59 | [NM_068230.3](http://www.ncbi.nlm.nih.gov/nucleotide/133907394?report=genbank&log$=nucltop&blast_rank=1&RID=62E96E1D016) | hypothetical protein (Y17G9B.5) = unknown ECSIT? | 33% | 1,00E-34 |
|  | IRAK 4 | HE609065 | [NM_057623.3](http://www.ncbi.nlm.nih.gov/nucleotide/24650578?report=genbank&log$=nucltop&blast_rank=1&RID=3T47F8KB011) | pelle | 21% | 1,00E-21 | [AF348167.1](http://www.ncbi.nlm.nih.gov/nucleotide/13650029?report=genbank&log$=nucltop&blast_rank=1&RID=3T482KA901P) | Pelle IRAK-like kinase 1 | 22% | 1,00E-29 |
|  | Jun/AP1 | HE609066 | [NM_057238.4](http://www.ncbi.nlm.nih.gov/nucleotide/281363083?report=genbank&log$=nucltop&blast_rank=3&RID=3V1V7VES014) | Jun-related antigen | 21% | 6,00E-21 | nd |  |  |  |
|  | *Me1*-LBP | HE609067 | nd |  |  |  | nd |  |  |  |
|  | *Me2*-LBP | HE609068 | nd |  |  |  | nd |  |  |  |
|  | *Me3*-LBP | HE609069 | nd |  |  |  | nd |  |  |  |
|  | *Me4*-LBP | HE609070 | nd |  |  |  | nd |  |  |  |
|  | Myd88 | HE609071 | nd |  |  |  | nd |  |  |  |
|  | p38 MAPK | HE609072 | [AB006364.1](http://www.ncbi.nlm.nih.gov/nucleotide/4038643?report=genbank&log$=nucltop&blast_rank=3&RID=62KTY1V1011) | p38 MAPK | 52% | 2,00E-126 | [NM_068964.2](http://www.ncbi.nlm.nih.gov/nucleotide/86563614?report=genbank&log$=nucltop&blast_rank=1&RID=62M5HMT4013) | P38 Map Kinase family member (pmk-1) | 51% | 1,00E-128 |
|  | SARM | HE609073 | [NM_001104074.1](http://www.ncbi.nlm.nih.gov/nucleotide/161082209?report=genbank&log$=nucltop&blast_rank=1&RID=5K8DNZX201N) | Ect4 | 86% | 2,00E-60 | [AY834227.1](http://www.ncbi.nlm.nih.gov/nucleotide/56434535?report=genbank&log$=nucltop&blast_rank=2&RID=5K8E0VR8016) | Toll and interleukin 1 receptor domain protein | 81% | 4,00E-44 |
|  | TOLLIP | HE609074 | nd |  |  |  | [NM_060356.2](http://www.ncbi.nlm.nih.gov/nucleotide/212641725?report=genbank&log$=nucltop&blast_rank=1&RID=3SUD5EPA01R) | Tollip homolog family member (tli-1) | 49% | 5,00E-57 |
|  | TRAF6 | HE609075 | [AF111422.1](http://www.ncbi.nlm.nih.gov/nucleotide/10716151?report=genbank&log$=nucltop&blast_rank=1&RID=3PEJXZB001P) | Traf1 | 41% | 2,00E-38 | [AF348168.1](http://www.ncbi.nlm.nih.gov/nucleotide/13650032?report=genbank&log$=nucltop&blast_rank=1&RID=3PEK5RVY01R) | Traf1 | 29% | 4,00E-25 |
|  | TRAM (TICAM 2) | HE609076 | nd |  |  |  | nd |  |  |  |
|  | FADD | HE609077 | nd |  |  |  | nd |  |  |  |
|  | MDA-5 | HE609078 | [NM_079054.3](http://www.ncbi.nlm.nih.gov/nucleotide/45549182?report=genbank&log$=nucltop&blast_rank=5&RID=3UXAPEPF014) | Dicer-2 | 14% | 2,00E-51 | [AF480439.1](http://www.ncbi.nlm.nih.gov/nucleotide/21703171?report=genbank&log$=nucltop&blast_rank=1&RID=3UXAXM9D011) | dicer-related helicase | 26% | 5,00E-25 |
|  | DDX58 (RIG-I) | HE609079 | [NM_079054.3](http://www.ncbi.nlm.nih.gov/nucleotide/45549182?report=genbank&log$=nucltop&blast_rank=2&RID=3UWHN4TR016) | Dicer-2 | 19% | 1,00E-12 | [AF480440.1](http://www.ncbi.nlm.nih.gov/nucleotide/21703170?report=genbank&log$=nucltop&blast_rank=1&RID=3UWHWERE016) | dicer-related helicase-like | 35% | 3,00E-33 |
|  | *Me1*-PGRP | HE609080 | [AJ556620.1](http://www.ncbi.nlm.nih.gov/nucleotide/37665336?report=genbank&log$=nucltop&blast_rank=1&RID=3VF9ZP8U01R) | PGRP-SC2 | 56% | 1,00E-46 | nd |  |  |  |
|  | *Me2*-PGRP | HE609081 | [AJ556620.1](http://www.ncbi.nlm.nih.gov/nucleotide/37665336?report=genbank&log$=nucltop&blast_rank=4&RID=3VFKBXGM014) | PGRP-SC2 | 33% | 4,00E-51 | [NM_062046.1](http://www.ncbi.nlm.nih.gov/nucleotide/17536962?report=genbank&log$=nucltop&blast_rank=1&RID=3VFKJRJE014) | C-type LECtin family member (clec-122) | 17% | 2,00E-05 |
|  | *Me3-*PGRP | HE609082 | [AY327467.1](http://www.ncbi.nlm.nih.gov/nucleotide/33355915?report=genbank&log$=nucltop&blast_rank=3&RID=3VFYP2PH01P) | PGRP-LC | 28% | 9,00E-13 | nd |  |  |  |
|  | *Me4*-PGRP | HE609083 | nd |  |  |  | nd |  |  |  |
|  | *Me5*-PGRP | HE609084 | [NM_001169926.1](http://www.ncbi.nlm.nih.gov/nucleotide/281365922?report=genbank&log$=nucltop&blast_rank=4&RID=3VH283VB014) | PGRP-LC | 49% | 7,00E-36 | nd |  |  |  |
|  | *Me6-*PGRP | HE609085 | [AJ556616.1](http://www.ncbi.nlm.nih.gov/nucleotide/37665328?report=genbank&log$=nucltop&blast_rank=1&RID=3VHVP6ZD011) | PGRP-SC2 | 50% | 9,00E-42 | nd |  |  |  |
|  | *Me7-*PGRP | HE609086 | [AJ556620.1](http://www.ncbi.nlm.nih.gov/nucleotide/37665336?report=genbank&log$=nucltop&blast_rank=1&RID=3VJY882J011) | PGRP-SC2 | 63% | 9,00E-09 | nd |  |  |  |
|  | *Me8*-PGRP | HE609087 | [NM_001169926.1](http://www.ncbi.nlm.nih.gov/nucleotide/281365922?report=genbank&log$=nucltop&blast_rank=1&RID=3VKBU5AN014) | PGRP-LC | 60% | 2,00E-46 | nd |  |  |  |
|  | *Me1*-GNBP | HE609088 | [AF228474.1](http://www.ncbi.nlm.nih.gov/nucleotide/6969636?report=genbank&log$=nucltop&blast_rank=15&RID=3VSP93ZH016) | GNBP3 | 40% | 5,00E-09 | nd |  |  |  |
|  | *Me2*-GNBP | HE609089 | [NM_079418.2](http://www.ncbi.nlm.nih.gov/nucleotide/24666389?report=genbank&log$=nucltop&blast_rank=1&RID=3VRMPA87016) | GNBP1 | 20% | 3,00E-24 | nd |  |  |  |
|  | *Me3*-GNBP | HE609090 | [NM_079418.2](http://www.ncbi.nlm.nih.gov/nucleotide/24666389?report=genbank&log$=nucltop&blast_rank=1&RID=3VSF3680014) | GNBP1 | 27% | 6,00E-25 | nd |  |  |  |
|  | TBK1 | HE609091 | [NM_165337.1](http://www.ncbi.nlm.nih.gov/nucleotide/24585470?report=genbank&log$=nucltop&blast_rank=5&RID=40JCCGJD016) | IkappaB kinase-like 2 | 34% | 3,00E-42 | nd |  |  |  |
|  | TAK1 | HE609092 | nd |  |  |  | nd |  |  |  |
|  | A20 | HE609093 | [NM_141674.2](http://www.ncbi.nlm.nih.gov/nucleotide/24645472?report=genbank&log$=nucltop&blast_rank=5&RID=40UNY4CW011) | trabid | 7% | 4,00E-14 | nd |  |  |  |
|  | JAK 2 | HE609094 | [NM_139602.2](http://www.ncbi.nlm.nih.gov/nucleotide/24657233?report=genbank&log$=nucltop&blast_rank=4&RID=40AF7P13016) | Ack | 32% | 1,00E-38 | [NM_078382.5](http://www.ncbi.nlm.nih.gov/nucleotide/212646689?report=genbank&log$=nucltop&blast_rank=2&RID=40AFDZ26011) | protein KINase family member (kin-25) | 24% | 4,00E-32 |
|  | *Me1*-STAT | HE609095 | [NM_169900.2](http://www.ncbi.nlm.nih.gov/nucleotide/28572151?report=genbank&log$=nucltop&blast_rank=2&RID=40B9UN0G011) | STAT 92E | 62% | 8,00E-34 | [NM_001129342.1](http://www.ncbi.nlm.nih.gov/nucleotide/193206816?report=genbank&log$=nucltop&blast_rank=1&RID=40BA0ZNH016) | STAT transcription factor family member (sta-1) | 34% | 8,00E-16 |
|  | *Me2-*STAT | HE609096 | nd |  |  |  | nd |  |  |  |
|  | *Me3*-STAT | HE609097 | nd |  |  |  | nd |  |  |  |
|  | *Me1-*SOCS | HE609098 | [NM_078666.3](http://www.ncbi.nlm.nih.gov/nucleotide/161077912?report=genbank&log$=nucltop&blast_rank=3&RID=40FMM8XJ011) | SOCS 16D | 12% | 1,00E-06 | nd |  |  |  |
|  | *Me2*-SOCS | HE609099 | [NM_165243.1](http://www.ncbi.nlm.nih.gov/nucleotide/24584929?report=genbank&log$=nucltop&blast_rank=1&RID=40HARCNB014) | SOCS 36E | 18% | 4,00E-80 | nd |  |  |  |
| Cytokines | IL-16 | HE609100 | [NM_140447.2](http://www.ncbi.nlm.nih.gov/nucleotide/221331171?report=genbank&log$=nucltop&blast_rank=1&RID=3UU05749011) | big bang | 32% | 4,00E-15 | [NM_069818.2](http://www.ncbi.nlm.nih.gov/nucleotide/71990719?report=genbank&log$=nucltop&blast_rank=1&RID=3UU0D9A1011) | magi-1 | 26% | 6,00E-08 |
|  | *Me1*-IL-17 | HE609101 | nd |  |  |  | nd |  |  |  |
|  | *Me2*-IL-17 | HE609102 | nd |  |  |  | nd |  |  |  |
|  | *Me1-*MIF | HE609103 | nd |  |  |  | [NM_067135.4](http://www.ncbi.nlm.nih.gov/nucleotide/193211013?report=genbank&log$=nucltop&blast_rank=1&RID=4BE7DZFX01P) | MIF -1 | 42% | 2,00E-13 |
|  | *Me2-*MIF | HE609104 | nd |  |  |  | [NM_073602.4](http://www.ncbi.nlm.nih.gov/nucleotide/193207527?report=genbank&log$=nucltop&blast_rank=1&RID=4BE7DZFX01P) | MIF -2 | 42% | 4,00E-08 |
|  | *Me3-*MIF | HE609105 | nd |  |  |  | [NM_067135.4](http://www.ncbi.nlm.nih.gov/nucleotide/193211013?report=genbank&log$=nucltop&blast_rank=1&RID=4BE7DZFX01P) | MIF -1 | 38% | 3,00E-12 |
|  | *Me4-*MIF | HE609106 | nd |  |  |  | [NM_073602.4](http://www.ncbi.nlm.nih.gov/nucleotide/193207527?report=genbank&log$=nucltop&blast_rank=1&RID=4BE7DZFX01P) | MIF -2 | 55% | 5,00E-15 |
|  | *Me5-*MIF | HE609107 | nd |  |  |  | [NM_073602.4](http://www.ncbi.nlm.nih.gov/nucleotide/193207527?report=genbank&log$=nucltop&blast_rank=1&RID=4BE7DZFX01P) | MIF -2 | 58% | 8,00E-14 |
| Complement system | *Me1-*C3 | HE609108 | [AJ973205.1](http://www.ncbi.nlm.nih.gov/nucleotide/119116167?report=genbank&log$=nucltop&blast_rank=1&RID=40VCWGH401P) | TepI gene for thiolester containing protein I | 47% | 2,00E-18 | nd |  |  |  |
|  | *Me2*-C3 | HE609109 | nd |  |  |  | nd |  |  |  |
|  | C6 | HE609110 | nd |  |  |  | nd |  |  |  |
|  | DMBT1 | HE609111 | [NM_168316.2](http://www.ncbi.nlm.nih.gov/nucleotide/281365902?report=genbank&log$=nucltop&blast_rank=1&RID=48X85FHD016) | tequila | 16% | 2,00E-31 | nd |  |  |  |
|  | ATG3 | HE609112 | [NM_140802.2](http://www.ncbi.nlm.nih.gov/nucleotide/24666430?report=genbank&log$=nucltop&blast_rank=1&RID=4B2EPMSF014) | Aut1 | 67% | 1,00E-123 | [NM_067623.3](http://www.ncbi.nlm.nih.gov/nucleotide/193206857?report=genbank&log$=nucltop&blast_rank=1&RID=4B2EW58P014) | ATG3 | 57% | 3,00E-88 |
|  | *Me1-*ATG4B | HE609113 | [NM_134719.3](http://www.ncbi.nlm.nih.gov/nucleotide/281360504?report=genbank&log$=nucltop&blast_rank=5&RID=4B4UVR4V016) | ATG4 | 39% | 1,00E-27 | [NM_060974.3](http://www.ncbi.nlm.nih.gov/nucleotide/212645204?report=genbank&log$=nucltop&blast_rank=1&RID=4B4V2G6F01P) | ATG4.1 | 40% | 6,00E-27 |
|  | *Me2-*ATG4B | HE609114 | [NM_134719.3](http://www.ncbi.nlm.nih.gov/nucleotide/281360504?report=genbank&log$=nucltop&blast_rank=2&RID=4B566FBB016) | ATG4 | 71% | 5,00E-55 | [NM_060974.3](http://www.ncbi.nlm.nih.gov/nucleotide/212645204?report=genbank&log$=nucltop&blast_rank=1&RID=4B56C8T5016) | ATG4.1 | 65% | 3,00E-53 |
|  | ATG4C | HE609115 | [NM_134719.3](http://www.ncbi.nlm.nih.gov/nucleotide/281360504?report=genbank&log$=nucltop&blast_rank=9&RID=4B5TD3WG011) | ATG4 | 39% | 4,00E-39 | [NM_069807.2](http://www.ncbi.nlm.nih.gov/nucleotide/71999797?report=genbank&log$=nucltop&blast_rank=1&RID=4B65XGAJ014) | ATG4.2 | 43% | 5,00E-42 |
|  | ATG5 | HE609116 | [NM_132162.2](http://www.ncbi.nlm.nih.gov/nucleotide/24640354?report=genbank&log$=nucltop&blast_rank=2&RID=4B17DDNW011) | ATG5 | 65% | 5,00E-51 | [NM_058484.4](http://www.ncbi.nlm.nih.gov/nucleotide/193203631?report=genbank&log$=nucltop&blast_rank=1&RID=4B17MKJE016) | ATG5 | 35% | 2,00E-18 |
|  | ATG7 | HE609117 | [NM_137506.2](http://www.ncbi.nlm.nih.gov/nucleotide/24655130?report=genbank&log$=nucltop&blast_rank=2&RID=4B3GYKNS01P) | ATG7 | 42% | 7,00E-123 | [NM_069663.2](http://www.ncbi.nlm.nih.gov/nucleotide/71992133?report=genbank&log$=nucltop&blast_rank=1&RID=4B3HAT8801R) | ATG7 | 48% | 4,00E-115 |
| Autophagy / Apoptosis | ATG 8 (LC3) | HE609118 | [NM_167245.2](http://www.ncbi.nlm.nih.gov/nucleotide/281360683?report=genbank&log$=nucltop&blast_rank=1&RID=4B0PRV50011) | ATG 8 | 12% | 4,00E-07 | [NM_069634.2](http://www.ncbi.nlm.nih.gov/nucleotide/71999766?report=genbank&log$=nucltop&blast_rank=1&RID=4B0PWDMS011) | LC3, GABARAP and GATE-16 family member (lgg-2) | 21% | 1,00E-40 |
|  | ATG9 | HE609119 | [NM_137270.2](http://www.ncbi.nlm.nih.gov/nucleotide/22024174?report=genbank&log$=nucltop&blast_rank=7&RID=4B7W0WFY016) | ATG9 | 99% | 4,00E-81 | [AM085500.1](http://www.ncbi.nlm.nih.gov/nucleotide/74831728?report=genbank&log$=nucltop&blast_rank=3&RID=4B7W8ZXC016) | ATG9 | 84% | 6,00E-46 |
|  | ATG12 | HE609120 | [NM_140294.3](http://www.ncbi.nlm.nih.gov/nucleotide/221331103?report=genbank&log$=nucltop&blast_rank=1&RID=4B1ZFYEZ014) | ATG12 | 26% | 3,00E-32 | [NM_065827.1](http://www.ncbi.nlm.nih.gov/nucleotide/17554203?report=genbank&log$=nucltop&blast_rank=1&RID=4B1ZNRAF014) | LC3, GABARAP and GATE-16 family member (lgg-3) | 19% | 3,00E-08 |
|  | ATG16 | HE609121 | [NM_170434.4](http://www.ncbi.nlm.nih.gov/nucleotide/221460424?report=genbank&log$=nucltop&blast_rank=1&RID=4B6MXFC901R) | CG31033 (ATG16?) | 84% | 2,00E-37 | nd |  |  |  |
|  | Beclin1 | HE609122 | [NM_142952.2](http://www.ncbi.nlm.nih.gov/nucleotide/24649522?report=genbank&log$=nucltop&blast_rank=1&RID=491ENSXJ011) | ATG6 | 67% | 5,00E-124 | [NM_068443.2](http://www.ncbi.nlm.nih.gov/nucleotide/71994163?report=genbank&log$=nucltop&blast_rank=1&RID=491EW7J8011) | Beclin1 | 46% | 6,00E-32 |
|  | mTOR | HE609123 | [NM_080152.2](http://www.ncbi.nlm.nih.gov/nucleotide/24583993?report=genbank&log$=nucltop&blast_rank=1&RID=4B92J8FX014) | mTOR | 72% | 3,00E-18 | nd |  |  |  |
|  | AIFM1 | HE609124 | [NM_134805.3](http://www.ncbi.nlm.nih.gov/nucleotide/28573991?report=genbank&log$=nucltop&blast_rank=4&RID=4AY1267D011) | AIF | 63% | 3,00E-95 | [NM_067163.3](http://www.ncbi.nlm.nih.gov/nucleotide/71995310?report=genbank&log$=nucltop&blast_rank=2&RID=4AY174XA011) | AIF | 51% | 1,00E-60 |
|  | AIFM3 (AFL) | HE609125 | [AC009205.6](http://www.ncbi.nlm.nih.gov/nucleotide/13384324?report=genbank&log$=nucltop&blast_rank=1&RID=4AYN71P301P) | BAC clone BACR04C20 (AIF?) | 42% | 2,00E-98 | [NM_072711.3](http://www.ncbi.nlm.nih.gov/nucleotide/86563915?report=genbank&log$=nucltop&blast_rank=1&RID=4AYNE27X016) | hypothetical protein (oxidoreductase) , | 41% | 1,00E-94 |
|  | Bak | HE609126 | nd |  |  |  | nd |  |  |  |
|  | Bax | HE609127 | nd |  |  |  | nd |  |  |  |
|  | *Me1*-BCL2 | HE609128 | [NM_078978.1](http://www.ncbi.nlm.nih.gov/nucleotide/24652798?report=genbank&log$=nucltop&blast_rank=3&RID=4AUHZY24016) | buffy | 24% | 2,00E-09 | [NM_066883.4](http://www.ncbi.nlm.nih.gov/nucleotide/212645871?report=genbank&log$=nucltop&blast_rank=1&RID=4AUJ753U011) | Cell Death  abnormality family member (ced-9) | 34% | 1,00E-06 |
|  | *Me2-*BCL2 | HE609129 | nd |  |  |  | nd |  |  |  |
|  | BCLxL | HE609130 | [NM_078978.1](http://www.ncbi.nlm.nih.gov/nucleotide/24652798?report=genbank&log$=nucltop&blast_rank=4&RID=4AVSJ3X401R) | buffy | 11% | 2,00E-09 | nd |  |  |  |
|  | initiator caspase with CARD domain | HE609131 | [NM_057626.3](http://www.ncbi.nlm.nih.gov/nucleotide/24762322?report=genbank&log$=nucltop&blast_rank=12&RID=4BA54925016) | death casp-1 | 10% | 2,00E-06 | [AF210702.1](http://www.ncbi.nlm.nih.gov/nucleotide/11967320?report=genbank&log$=nucltop&blast_rank=1&RID=4BA5BSX701R) | casp CED-3 | 39% | 7,00E-15 |

**Mollusca & Bivalves.**

**Species names abbreviated in the table: *Argopecten irradians, Biomphalaria glabrata, Chlamys farreri, Crassostrea angulata, Crassostrea gigas, Euprymna scolopes, Haliotis cracherodii, Haliotis discus discus, Haliotis diversicolor, Haliotis diversicolor supertexta, Littorina littorea, Mytilus californianus, Mytilus edulis, Mytilus galloprovincialis, Meretrix meretrix, Pinctada fucata, Pseudocardium sachalinensis, Venerupis decussatus.***

|  |  |  | Mollusca |  |  |  |  | Bivalvia |  |  |  |  |
| --- | --- | --- | --- | --- | --- | --- | --- | --- | --- | --- | --- | --- |
| Category | Gene | *M. edulis*  Accession | Accession | Species | Description | Query cov. | E-value | Accession | Species | Description | Query cov. | E-value |
| Transcription factors | *Me1-*IRF | HE609042 | nd |  |  |  |  | nd |  |  |  |  |
|  | *Me2-*IRF | HE609043 | nd |  |  |  |  | nd |  |  |  |  |
|  | *Me3-*IRF | HE609044 | nd |  |  |  |  | nd |  |  |  |  |
|  | *Me4-*IRF | HE609045 | nd |  |  |  |  | nd |  |  |  |  |
|  | *Me5-*IRF | HE609046 | nd |  |  |  |  | nd |  |  |  |  |
|  | LITAF | HE609047 | [GU217721.1](http://www.ncbi.nlm.nih.gov/nucleotide/282153488?report=genbank&log$=nucltop&blast_rank=1&RID=FFC5M1AG016) | *M. meretrix* | LITAF | 18% | 4,00E-38 | [GU217721.1](http://www.ncbi.nlm.nih.gov/nucleotide/282153488?report=genbank&log$=nucltop&blast_rank=1&RID=FFC5VRSR014) | *M. meretrix* | LITAF | 18% | 7,00E-39 |
|  | *NF-κB* p65 | HE609048 | [EF121959.1](http://www.ncbi.nlm.nih.gov/nucleotide/119351134?report=genbank&log$=nucltop&blast_rank=1&RID=3PM4XK39014) | *P. fucata* | NF-*κ*B | 38% | 0.0 | [EF121959.1](http://www.ncbi.nlm.nih.gov/nucleotide/119351134?report=genbank&log$=nucltop&blast_rank=1&RID=3PM4XK39014) | *P. fucata* | NF-*κ*B | 38% | 0.0 |
|  | *Me1*-*NF-κB* 1 p50 | HE609049 | [EU871726.1](http://www.ncbi.nlm.nih.gov/nucleotide/194718236?report=genbank&log$=nucltop&blast_rank=1&RID=3SPJUHVU011) | *P. fucata* | inhibitor of nuclear factor-*κ*B protein | 16% | 2,00E-22 | [EU871726.1](http://www.ncbi.nlm.nih.gov/nucleotide/194718236?report=genbank&log$=nucltop&blast_rank=1&RID=3SNZ1D3W011) | *P. fucata* | inhibitor of nuclear factor-*κ*B protein | 16% | 7,00E-23 |
|  | *Me2*-*NF-κB* 1 p50 | HE609050 | [EF121959.1](http://www.ncbi.nlm.nih.gov/nucleotide/119351134?report=genbank&log$=nucltop&blast_rank=1&RID=A47B4HF4014) | *P. fucata* | NF-*κ*B | 41% | 2,00E-71 | [EF121959.1](http://www.ncbi.nlm.nih.gov/nucleotide/119351134?report=genbank&log$=nucltop&blast_rank=1&RID=A47BT2HM011) | *P. fucata* | NF-*κ*B | 41% | 6,00E-72 |
| PRR pathway proteins | Caspase 3 subunit p12 | HE609051 | [EF080944.1](http://www.ncbi.nlm.nih.gov/nucleotide/122056407?report=genbank&log$=nucltop&blast_rank=2&RID=3V5V3EWW011) | *M. galloprovincialis* | putative caspase 3 | 4% | 2,00E-18 | [EF080944.1](http://www.ncbi.nlm.nih.gov/nucleotide/122056407?report=genbank&log$=nucltop&blast_rank=1&RID=3V5VA16U01R) | *M. galloprovincialis* | putative caspase 3 | 4% | 6,00E-19 |
|  | *Me1-*TNFR | HE609052 | [FJ607953.1](http://www.ncbi.nlm.nih.gov/nucleotide/255929999?report=genbank&log$=nucltop&blast_rank=1&RID=3Y3TNRW5011) | *C. farreri* | TNFR | 5% | 0.002 | [FJ607953.1](http://www.ncbi.nlm.nih.gov/nucleotide/255929999?report=genbank&log$=nucltop&blast_rank=1&RID=3Y3U37EY016) | *C. farreri* | TNFR | 5% | 7,00E-04 |
|  | *Me2-*TNFR | HE609053 | [FJ607953.1](http://www.ncbi.nlm.nih.gov/nucleotide/255929999?report=genbank&log$=nucltop&blast_rank=1&RID=3Y4JMYBM016) | *C. farreri* | TNFR | 19% | 3,00E-08 | [FJ607953.1](http://www.ncbi.nlm.nih.gov/nucleotide/255929999?report=genbank&log$=nucltop&blast_rank=1&RID=3Y4JW4E3014) | *C. farreri* | TNFR | 19% | 8,00E-09 |
|  | *Me3*-TNFR | HE609054 | [FJ607953.1](http://www.ncbi.nlm.nih.gov/nucleotide/255929999?report=genbank&log$=nucltop&blast_rank=1&RID=3Y56DVXE014) | *Ch. farreri* | TNFR | 6% | 0.002 | [FJ607953.1](http://www.ncbi.nlm.nih.gov/nucleotide/255929999?report=genbank&log$=nucltop&blast_rank=1&RID=3Y56HD31016) | *C. farreri* | TNFR | 6% | 5,00E-04 |
|  | *Me1*-TRAF | HE609055 | [DQ350773.1](http://www.ncbi.nlm.nih.gov/nucleotide/85687487?report=genbank&log$=nucltop&blast_rank=1&RID=3YACFR5K01R) | *C. farreri* | Traf6 | 50% | 1,00E-21 | [DQ350773.1](http://www.ncbi.nlm.nih.gov/nucleotide/85687487?report=genbank&log$=nucltop&blast_rank=1&RID=3YACT85S01P) | *C. farreri* | Traf6 | 50% | 3,00E-22 |
|  | *Me2*-TRAF | HE609056 | [AY956816.1](http://www.ncbi.nlm.nih.gov/nucleotide/63034002?report=genbank&log$=nucltop&blast_rank=1&RID=3YDYA977014) | *E. scolopes* | Traf6 | 78% | 9,00E-34 | [DQ350773.1](http://www.ncbi.nlm.nih.gov/nucleotide/85687487?report=genbank&log$=nucltop&blast_rank=1&RID=3YDZ6SE8011) | *C. farreri* | Traf6 | 88% | 2,00E-33 |
|  | *Me3*-TRAF | HE609057 | [DQ350773.1](http://www.ncbi.nlm.nih.gov/nucleotide/85687487?report=genbank&log$=nucltop&blast_rank=2&RID=3YB6S1XT011) | *C. farreri* | Traf6 | 43% | 4,00E-141 | [DQ350773.1](http://www.ncbi.nlm.nih.gov/nucleotide/85687487?report=genbank&log$=nucltop&blast_rank=1&RID=3YB75CP701R) | *C. farreri* | Traf6 | 43% | 1,00E-14 |
|  | *Me4*-TRAF | HE609058 | [AY956816.1](http://www.ncbi.nlm.nih.gov/nucleotide/63034002?report=genbank&log$=nucltop&blast_rank=1&RID=3YCTA9BH01P) | *E. scolopes* | Traf6 | 59% | 1,00E-01 | [DQ350773.1](http://www.ncbi.nlm.nih.gov/nucleotide/85687487?report=genbank&log$=nucltop&blast_rank=1&RID=3YDF4MT9011) | *C. farreri* | Traf6 | 55% | 2,00E-12 |
|  | *Me1*-I*κ*B | HE609059 | [EU871726.1](http://www.ncbi.nlm.nih.gov/nucleotide/194718236?report=genbank&log$=nucltop&blast_rank=1&RID=3PG6HBYU01P) | *P. fucata* | I*κ*B | 36% | 1,00E-70 | [EU871726.1](http://www.ncbi.nlm.nih.gov/nucleotide/194718236?report=genbank&log$=nucltop&blast_rank=1&RID=3PG6UWSG01R) | *P. fucata* | I*κ*B | 36% | 4,00E-71 |
|  | *Me2*-I*κ*B | HE609060 | [FJ824733.1](http://www.ncbi.nlm.nih.gov/nucleotide/269306944?report=genbank&log$=nucltop&blast_rank=1&RID=3PH1CEFP01R) | *A. irradians* | I*κ*B | 43% | 5,00E-80 | [FJ824733.1](http://www.ncbi.nlm.nih.gov/nucleotide/269306944?report=genbank&log$=nucltop&blast_rank=1&RID=3PH1KXN601P) | *A. irradians* | I*κ*B | 43% | 2,00E-80 |
|  | IKK | HE609061 | [AY818355.1](http://www.ncbi.nlm.nih.gov/nucleotide/61815767?report=genbank&log$=nucltop&blast_rank=1&RID=3PJ82S8T01P) | *P. fucata* | IKK-like | 94% | 9,00E-130 | [AY818355.1](http://www.ncbi.nlm.nih.gov/nucleotide/61815767?report=genbank&log$=nucltop&blast_rank=1&RID=3PJ82S8T01P) | *P. fucata* | IKK-like | 94% | 9,00E-130 |
|  | *Me1*-caspase 8 | HE609062 | [FJ937785.1](http://www.ncbi.nlm.nih.gov/nucleotide/284159257?report=genbank&log$=nucltop&blast_rank=1&RID=3V321588014) | *M. californianus* | casp 8 | 39% | 3,00E-36 | [FJ937785.1](http://www.ncbi.nlm.nih.gov/nucleotide/284159257?report=genbank&log$=nucltop&blast_rank=1&RID=3V328EGV011) | *M. californianus* | casp 8 | 39% | 9,00E-37 |
|  | *Me2*-caspase 8 | HE609063 | [FJ937784.1](http://www.ncbi.nlm.nih.gov/nucleotide/284159255?report=genbank&log$=nucltop&blast_rank=1&RID=3V4V5CZP016) | *M. californianus* | casp-8-like protein | 100% | 1,00E-88 | [FJ937784.1](http://www.ncbi.nlm.nih.gov/nucleotide/284159255?report=genbank&log$=nucltop&blast_rank=1&RID=3V4RA1UY016) | *M. californianus* | casp-8-like protein | 100% | 3,00E-89 |
|  | ECSIT | HE609064 | [GU207418.1](http://www.ncbi.nlm.nih.gov/nucleotide/270063425?report=genbank&log$=nucltop&blast_rank=1&RID=62EA0CWP013) | *C. gigas* | clone CG_Ba127F5 | 59% | 7,00E-73 | [GU207418.1](http://www.ncbi.nlm.nih.gov/nucleotide/270063425?report=genbank&log$=nucltop&blast_rank=1&RID=62EAA7NK013) | *C. gigas* | clone CG_Ba127F5 | 59% | 2,00E-73 |
|  | IRAK 4 | HE609065 | [GU351882.1](http://www.ncbi.nlm.nih.gov/nucleotide/288551188?report=genbank&log$=nucltop&blast_rank=1&RID=3T4MNJW8014) | *H. diversicolor* | IRAK 4 | 70% | 4,00E-104 | [GQ454915.1](http://www.ncbi.nlm.nih.gov/nucleotide/258677221?report=genbank&log$=nucltop&blast_rank=1&RID=3T49BHJ6011) | *M. galloprovincialis* | extracell. regulated protein kinase | 6% | 1,00E-05 |
|  | Jun/AP1 | HE609066 | nd |  |  |  |  | nd |  |  |  |  |
|  | *Me1*-LBP | HE609067 | [FJ669299.1](http://www.ncbi.nlm.nih.gov/nucleotide/229472823?report=genbank&log$=nucltop&blast_rank=1&RID=3NX7YKY101R) | *C. gigas* | bpi | 73% | 7,00E-104 | [FJ669299.1](http://www.ncbi.nlm.nih.gov/nucleotide/229472823?report=genbank&log$=nucltop&blast_rank=1&RID=3NX7YKY101R) | *C. gigas* | bpi | 73% | 7,00E-104 |
|  | *Me2*-LBP | HE609068 | [FJ669312.1](http://www.ncbi.nlm.nih.gov/nucleotide/229472849?report=genbank&log$=nucltop&blast_rank=1&RID=3NYKCYDE01P) | *C. gigas* | bpi | 73% | 9,00E-100 | [FJ669312.1](http://www.ncbi.nlm.nih.gov/nucleotide/229472849?report=genbank&log$=nucltop&blast_rank=1&RID=3NYJ6G2F01P) | *C. gigas* | bpi | 73% | 4,00E-100 |
|  | *Me3*-LBP | HE609069 | [FJ669321.1](http://www.ncbi.nlm.nih.gov/nucleotide/229472867?report=genbank&log$=nucltop&blast_rank=1&RID=3P9719J001P) | *C. gigas* | bpi | 55% | 1,00E-106 | [FJ669321.1](http://www.ncbi.nlm.nih.gov/nucleotide/229472867?report=genbank&log$=nucltop&blast_rank=1&RID=3P97ANTZ014) | *C. gigas* | bpi | 55% | 6,00E-107 |
|  | *Me4*-LBP | HE609070 | [FJ669315.1](http://www.ncbi.nlm.nih.gov/nucleotide/229472855?report=genbank&log$=nucltop&blast_rank=1&RID=3PAW5PXU014) | *C. gigas* | bpi | 59% | 4,00E-30 | [FJ669315.1](http://www.ncbi.nlm.nih.gov/nucleotide/229472855?report=genbank&log$=nucltop&blast_rank=1&RID=3PAWFXDU014) | *C. gigas* | bpi | 59% | 2,00E-30 |
|  | Myd88 | HE609071 | [DQ249918.1](http://www.ncbi.nlm.nih.gov/nucleotide/82468374?report=genbank&log$=nucltop&blast_rank=1&RID=3PCHCNNT01R) | *C. farreri* | Myd88 | 34% | 1,00E-54 | [DQ249918.1](http://www.ncbi.nlm.nih.gov/nucleotide/82468374?report=genbank&log$=nucltop&blast_rank=1&RID=3PCHRG9X01R) | *C. farreri* | Myd88 | 34% | 6,00E-55 |
|  | p38 MAPK | HE609072 | [AB375065.1](http://www.ncbi.nlm.nih.gov/nucleotide/169264925?report=genbank&log$=nucltop&blast_rank=1&RID=62KUMG9H01S) | *C. gigas* | p38 MAPK | 57% | 2,00E-164 | [AB375065.1](http://www.ncbi.nlm.nih.gov/nucleotide/169264925?report=genbank&log$=nucltop&blast_rank=1&RID=62KUU5MM01N) | *C. gigas* | p38 MAPK | 57% | 5,00E-165 |
|  | SARM | HE609073 | nd |  |  |  |  | nd |  |  |  |  |
|  | TOLLIP | HE609074 | [EU408785.1](http://www.ncbi.nlm.nih.gov/nucleotide/171466140?report=genbank&log$=nucltop&blast_rank=1&RID=3SUDUZYS01R) | *H. cracherodii* | TOLLIP | 20% | 2,00E-36 | nd |  |  |  |  |
|  | TRAF6 | HE609075 | [DQ350773.1](http://www.ncbi.nlm.nih.gov/nucleotide/85687487?report=genbank&log$=nucltop&blast_rank=1&RID=3PEKN0C901P) | *C. farreri* | Traf6 | 70% | 0.0 | [DQ350773.1](http://www.ncbi.nlm.nih.gov/nucleotide/85687487?report=genbank&log$=nucltop&blast_rank=1&RID=3PEKZ8E401P) | *C. farreri* | Traf6 | 70% | 0.0 |
|  | TRAM (TICAM 2) | HE609076 | nd |  |  |  |  | nd |  |  |  |  |
|  | FADD | HE609077 | [FJ937785.1](http://www.ncbi.nlm.nih.gov/nucleotide/284159257?report=genbank&log$=nucltop&blast_rank=1&RID=3VNAYENF011) | *M. californianus* | casp 8 | 18% | 8,00E-05 | [FJ937785.1](http://www.ncbi.nlm.nih.gov/nucleotide/284159257?report=genbank&log$=nucltop&blast_rank=1&RID=3VNB392G01R) | *M. californianus* | casp 8 | 18% | 2,00E-05 |
|  | MDA-5 | HE609078 | nd |  |  |  |  | nd |  |  |  |  |
|  | DDX58 (RIG-I) | HE609079 | nd |  |  |  |  | nd |  |  |  |  |
|  | *Me1*-PGRP | HE609080 | [AB425338.1](http://www.ncbi.nlm.nih.gov/nucleotide/187370565?report=genbank&log$=nucltop&blast_rank=1&RID=3VFATCEG014) | *C. gigas* | PGRP S3 | 76% | 9,00E-85 | [AB425338.1](http://www.ncbi.nlm.nih.gov/nucleotide/187370565?report=genbank&log$=nucltop&blast_rank=1&RID=3VFCW6PB014) | *C. gigas* | PGRP S3 | 76% | 3,00E-85 |
|  | *Me2*-PGRP | HE609081 | [AB425336.1](http://www.ncbi.nlm.nih.gov/nucleotide/187370561?report=genbank&log$=nucltop&blast_rank=1&RID=3VFM4TGH011) | *C. gigas* | PGRP S1L | 73% | 4,00E-113 | [AB425336.1](http://www.ncbi.nlm.nih.gov/nucleotide/187370561?report=genbank&log$=nucltop&blast_rank=1&RID=3VFMD4FS011) | *C. gigas* | PGRP S1L | 73% | 1,00E-113 |
|  | *Me3-*PGRP | HE609082 | [AB473552.1](http://www.ncbi.nlm.nih.gov/nucleotide/238768471?report=genbank&log$=nucltop&blast_rank=1&RID=3VFZFG2V014) | *C. gigas* | PGRP L | 31% | 4,00E-19 | [AB473552.1](http://www.ncbi.nlm.nih.gov/nucleotide/238768471?report=genbank&log$=nucltop&blast_rank=1&RID=3VFZSAYY014) | *C. gigas* | PGRP L | 31% | 1,00E-19 |
|  | *Me4*-PGRP | HE609083 | nd |  |  |  |  | nd |  |  |  |  |
|  | *Me5*-PGRP | HE609084 | [AB425336.1](http://www.ncbi.nlm.nih.gov/nucleotide/187370561?report=genbank&log$=nucltop&blast_rank=3&RID=3VH3245J01P) | *C. gigas* | PGRP S1L | 54% | 9,00E-45 | [AB425336.1](http://www.ncbi.nlm.nih.gov/nucleotide/187370561?report=genbank&log$=nucltop&blast_rank=3&RID=3VH380PE01R) | *C. gigas* | PGRP S1L | 54% | 3,00E-45 |
|  | *Me6-*PGRP | HE609085 | [EF452346.1](http://www.ncbi.nlm.nih.gov/nucleotide/133874729?report=genbank&log$=nucltop&blast_rank=2&RID=3VHWCA6C01P) | *B.glabrata* | M-line PGRP short form | 48% | 9,00E-60 | [AB425338.1](http://www.ncbi.nlm.nih.gov/nucleotide/187370565?report=genbank&log$=nucltop&blast_rank=1&RID=3VHWVZ0C016) | *C. gigas* | PGRP S3 | 50% | 4,00E-55 |
|  | *Me7-*PGRP | HE609086 | [AY987008.1](http://www.ncbi.nlm.nih.gov/nucleotide/66735050?report=genbank&log$=nucltop&blast_rank=1&RID=3VJYXX5F014) | *C. farreri* | PGRP S1 precursor | 67% | 3,00E-14 | [AY987008.1](http://www.ncbi.nlm.nih.gov/nucleotide/66735050?report=genbank&log$=nucltop&blast_rank=1&RID=3VK6H71P016) | *C. farreri* | PGRP S1 precursor | 67% | 1,00E-14 |
|  | *Me8*-PGRP | HE609087 | [AY956812.1](http://www.ncbi.nlm.nih.gov/nucleotide/63033994?report=genbank&log$=nucltop&blast_rank=1&RID=3VKCJTPJ01P) | *E. scolopes* | PGRP 2 precursor | 69% | 2,00E-70 | [AB425338.1](http://www.ncbi.nlm.nih.gov/nucleotide/187370565?report=genbank&log$=nucltop&blast_rank=1&RID=3VKCWM4S011) | *C. gigas* | PGRP S3 | 67% | 1,00E-63 |
|  | *Me1*-GNBP | HE609088 | [FJ755187.1](http://www.ncbi.nlm.nih.gov/nucleotide/225698611?report=genbank&log$=nucltop&blast_rank=1&RID=3VSPYUN2011) | *H. diversicolor supertexta* | β-GRP | 81% | 1,00E-81 | [AY308829.1](http://www.ncbi.nlm.nih.gov/nucleotide/32250972?report=genbank&log$=nucltop&blast_rank=1&RID=3VSR4KJP011) | *P. sachalinensis* | β-1,3-glucanase | 79% | 5,00E-49 |
|  | *Me2*-GNBP | HE609089 | [EF121825.1](http://www.ncbi.nlm.nih.gov/nucleotide/119350970?report=genbank&log$=nucltop&blast_rank=1&RID=3VRN9F68014) | *B.glabrata* | β-GRP | 47% | 1,00E-144 | [AY259542.1](http://www.ncbi.nlm.nih.gov/nucleotide/32442367?report=genbank&log$=nucltop&blast_rank=1&RID=3VRNH5N4011) | *C. farreri* | LGBP | 51% | 2,00E-143 |
|  | *Me3*-GNBP | HE609090 | [AB377114.1](http://www.ncbi.nlm.nih.gov/nucleotide/211926831?report=genbank&log$=nucltop&blast_rank=1&RID=3VSG1767016) | *C. gigas* | β-GRP 1 | 68% | 9,00E-176 | [AB377114.1](http://www.ncbi.nlm.nih.gov/nucleotide/211926831?report=genbank&log$=nucltop&blast_rank=1&RID=3VSG9J7K014) | *C. gigas* | β-GRP 1 | 68% | 3,00E-176 |
|  | TBK1 | HE609091 | nd |  |  |  |  | nd |  |  |  |  |
|  | TAK1 | HE609092 | [GQ222365.1](http://www.ncbi.nlm.nih.gov/nucleotide/255918295?report=genbank&log$=nucltop&blast_rank=1&RID=40KT58P801R) | *C. angulata* | TAK1 | 79% | 4,00E-115 | [GQ222365.1](http://www.ncbi.nlm.nih.gov/nucleotide/255918295?report=genbank&log$=nucltop&blast_rank=1&RID=40KTBYNG01R) | *C. angulata* | TAK1 | 79% | 1,00E-115 |
|  | A20 | HE609093 | nd |  |  |  |  | nd |  |  |  |  |
|  | JAK 2 | HE609094 | [AJ535669.1](http://www.ncbi.nlm.nih.gov/nucleotide/31335669?report=genbank&log$=nucltop&blast_rank=1&RID=40AFTKUE01R) | *C. gigas* | insulin-related peptide receptor | 26% | 1,00E-34 | [AJ535669.1](http://www.ncbi.nlm.nih.gov/nucleotide/31335669?report=genbank&log$=nucltop&blast_rank=1&RID=40AG3HRM01P) | *C. gigas* | insulin-related peptide receptor | 26% | 4,00E-35 |
|  | *Me1*-STAT | HE609095 | [DQ233650.1](http://www.ncbi.nlm.nih.gov/nucleotide/81336156?report=genbank&log$=nucltop&blast_rank=1&RID=40BAB93Y01P) | *L. littorea* | STAT | 21% | 4,00E-29 | nd |  |  |  |  |
|  | *Me2-*STAT | HE609096 | nd |  |  |  |  | nd |  |  |  |  |
|  | *Me3*-STAT | HE609097 | nd |  |  |  |  | nd |  |  |  |  |
|  | *Me1-*SOCS | HE609098 | [EU977134.1](http://www.ncbi.nlm.nih.gov/nucleotide/197700092?report=genbank&log$=nucltop&blast_rank=1&RID=40FNG3VW011) | *H. d. discus* | SOCS 2 | 22% | 9,00E-39 | nd |  |  |  |  |
|  | *Me2*-SOCS | HE609099 | [EU977134.1](http://www.ncbi.nlm.nih.gov/nucleotide/197700092?report=genbank&log$=nucltop&blast_rank=2&RID=40HBCJPP01P) | *H. d. discus* | SOCS 2 | 10% | 2,00E-04 | nd |  |  |  |  |
| Cytokines | IL-16 | HE609100 | nd |  |  |  |  | nd |  |  |  |  |
|  | *Me1*-IL-17 | HE609101 | A9XE49* | *C. gigas* | **IL 17-like protein** | 23% | 1,00E-03 | A9XE49* | *C. gigas* | **IL 17-like protein** | 23% | 1,00E-03 |
|  | *Me2*-IL-17 | HE609102 | nd |  |  |  |  | nd |  |  |  |  |
|  | *Me1-*MIF | HE609103 | [FJ435176.1](http://www.ncbi.nlm.nih.gov/nucleotide/215398884?report=genbank&log$=nucltop&blast_rank=1&RID=4BE80A4Y016) | *H. d. discus* | MIF | 50% | 1,00E-29 | nd |  |  |  |  |
|  | *Me2-*MIF | HE609104 | [GQ118971.1](http://www.ncbi.nlm.nih.gov/nucleotide/239509143?report=genbank&log$=nucltop&blast_rank=1&RID=4BE80A4Y016) | *B.glabrata* | MIF -2 | 43% | 5,00E-11 | nd |  |  |  |  |
|  | *Me3-*MIF | HE609105 | [FJ435176.1](http://www.ncbi.nlm.nih.gov/nucleotide/215398884?report=genbank&log$=nucltop&blast_rank=1&RID=4BE80A4Y016) | *H. d. discus* | MIF -2 | 46% | 8,00E-30 | nd |  |  |  |  |
|  | *Me4-*MIF | HE609106 | [GQ118971.1](http://www.ncbi.nlm.nih.gov/nucleotide/239509143?report=genbank&log$=nucltop&blast_rank=1&RID=4BE80A4Y016) | *B.glabrata* | MIF -2 | 55% | 1,00E-09 | nd |  |  |  |  |
|  | *Me5-*MIF | HE609107 | [GQ118971.1](http://www.ncbi.nlm.nih.gov/nucleotide/239509143?report=genbank&log$=nucltop&blast_rank=1&RID=4BE80A4Y016) | *B.glabrata* | MIF -2 | 58% | 5,00E-14 | nd |  |  |  |  |
| Complement system | *Me1-*C3 | HE609108 | [EU596375.1](http://www.ncbi.nlm.nih.gov/nucleotide/192383354?report=genbank&log$=nucltop&blast_rank=1&RID=40VDTUP2014) | *E. scolopes* | C3-like | 68% | 6,00E-40 | [FJ392025.1](http://www.ncbi.nlm.nih.gov/nucleotide/224037269?report=genbank&log$=nucltop&blast_rank=1&RID=40VDZGV2014) | *V. decussatus* | C3 | 58% | 1,00E-34 |
|  | *Me2*-C3 | HE609109 | [EU596375.1](http://www.ncbi.nlm.nih.gov/nucleotide/192383354?report=genbank&log$=nucltop&blast_rank=1&RID=48UP8YAM016) | *E. scolopes* | C3-like | 22% | 1,00E-05 | [EF210036.1](http://www.ncbi.nlm.nih.gov/nucleotide/144952811?report=genbank&log$=nucltop&blast_rank=1&RID=48UPNTZU014) | *C. farreri* | thioester-containing protein | 25% | 3,00E-05 |
|  | C6 | HE609110 | nd |  |  |  |  | nd |  |  |  |  |
|  | DMBT1 | HE609111 | [EF080941.1](http://www.ncbi.nlm.nih.gov/nucleotide/122056401?report=genbank&log$=nucltop&blast_rank=1&RID=48X8XZEB014) | *M. galloprovincialis* | putative uromodulin | 15% | 7,00E-121 | [EF080941.1](http://www.ncbi.nlm.nih.gov/nucleotide/122056401?report=genbank&log$=nucltop&blast_rank=1&RID=48X94VTU014) | *M. galloprovincialis* | putative uromodulin | 15% | 2,00E-121 |
|  | ATG3 | HE609112 | nd |  |  |  |  | nd |  |  |  |  |
|  | *Me1-*ATG4B | HE609113 | nd |  |  |  |  | nd |  |  |  |  |
|  | *Me2-*ATG4B | HE609114 | nd |  |  |  |  | nd |  |  |  |  |
|  | ATG4C | HE609115 | nd |  |  |  |  | nd |  |  |  |  |
|  | ATG5 | HE609116 | nd |  |  |  |  | nd |  |  |  |  |
|  | ATG7 | HE609117 | nd |  |  |  |  | nd |  |  |  |  |
| Autophagy / Apoptosis | ATG 8 (LC3) | HE609118 | [AJ308548.1](http://www.ncbi.nlm.nih.gov/nucleotide/15384733?report=genbank&log$=nucltop&blast_rank=1&RID=4B0RENBK011) | *M. edulis* | endo-1,4-β-D-glucanase | 17% | 5,00E-37 | [AJ308548.1](http://www.ncbi.nlm.nih.gov/nucleotide/15384733?report=genbank&log$=nucltop&blast_rank=1&RID=4B0R81CJ011) | *M. edulis* | endo-1,4-β-D-glucanase | 17% | 2,00E-36 |
|  | ATG9 | HE609119 | nd |  |  |  |  | nd |  |  |  |  |
|  | ATG12 | HE609120 | nd |  |  |  |  | nd |  |  |  |  |
|  | ATG16 | HE609121 | nd |  |  |  |  | nd |  |  |  |  |
|  | Beclin1 | HE609122 | nd |  |  |  |  | nd |  |  |  |  |
|  | mTOR | HE609123 | nd |  |  |  |  | nd |  |  |  |  |
|  | AIFM1 | HE609124 | nd |  |  |  |  | nd |  |  |  |  |
|  | AIFM3 (AFL) | HE609125 | nd |  |  |  |  | nd |  |  |  |  |
|  | Bak | HE609126 | nd |  |  |  |  | nd |  |  |  |  |
|  | Bax | HE609127 | nd |  |  |  |  | nd |  |  |  |  |
|  | *Me1*-BCL2 | HE609128 | [EU678310.1](http://www.ncbi.nlm.nih.gov/nucleotide/197091695?report=genbank&log$=nucltop&blast_rank=1&RID=4AUUXSNH01R) | *C. gigas* | predicted Bcl-2 | 46% | 1,00E-33 | [EU678310.1](http://www.ncbi.nlm.nih.gov/nucleotide/197091695?report=genbank&log$=nucltop&blast_rank=1&RID=4AUK1MDJ014) | *C. gigas* | predicted Bcl-2 | 46% | 3,00E-34 |
|  | *Me2-*BCL2 | HE609129 | nd |  |  |  |  |  |  |  |  |  |
|  | BCLxL | HE609130 | [EU678310.1](http://www.ncbi.nlm.nih.gov/nucleotide/197091695?report=genbank&log$=nucltop&blast_rank=1&RID=4AW89MGW016) | *C. gigas* | predicted Bcl-2 | 22% | 2,00E-34 | [EU678310.1](http://www.ncbi.nlm.nih.gov/nucleotide/197091695?report=genbank&log$=nucltop&blast_rank=1&RID=4AVUC79A011) | *C. gigas* | predicted Bcl-2 | 22% | 6,00E-35 |
|  | initiator caspase with CARD domain | HE609131 | [FJ937784.1](http://www.ncbi.nlm.nih.gov/nucleotide/284159255?report=genbank&log$=nucltop&blast_rank=1&RID=4BAFDBJ5016) | *M. californianus* | casp 8-like | 23% | 2,00E-10 | [FJ937784.1](http://www.ncbi.nlm.nih.gov/nucleotide/284159255?report=genbank&log$=nucltop&blast_rank=1&RID=4BA63M2P016) | *M. californianus* | casp 8-like | 23% | 5,00E-11 |

***Mytilus***

|  |  |  | *Mytilus* |  |  |  |  |
| --- | --- | --- | --- | --- | --- | --- | --- |
| Category | Gene | *M. edulis*  Accession | Accession | Species | Description | Query coverage | E-value |
| Transcription factors | *Me1-*IRF | HE609042 | nd |  |  |  |  |
|  | *Me2-*IRF | HE609043 | nd |  |  |  |  |
|  | *Me3-*IRF | HE609044 | nd |  |  |  |  |
|  | *Me4-*IRF | HE609045 | nd |  |  |  |  |
|  | *Me5-*IRF | HE609046 | nd |  |  |  |  |
|  | LITAF | HE609047 | nd |  |  |  |  |
|  | *NF-κB* p65 | HE609048 | [DQ673623.1](http://www.ncbi.nlm.nih.gov/nucleotide/110430503?report=genbank&log$=nucltop&blast_rank=1&RID=3PM5BTPY016) | *M. galloprovincialis* | Rel | 9% | 1,00E-71 |
|  | *Me1*-*NF-κB*1 p50 | HE609049 | nd |  |  |  |  |
|  | *Me2*- *NF-κB* 1 p50 | HE609050 | [HQ127223.1](http://www.ncbi.nlm.nih.gov/nucleotide/304636268?report=genbank&log$=nucltop&blast_rank=1&RID=A47CB9MX014) | *M. galloprovincialis* | Rel | 41% | 7,00E-70 |
| PRR pathway proteins | Caspase 3 subunit p12 | HE609051 | [EF080944.1](http://www.ncbi.nlm.nih.gov/nucleotide/122056407?report=genbank&log$=nucltop&blast_rank=1&RID=3V5VHNWR01R) | *M. galloprovincialis* | putative caspase 3 | 4% | 8,00E-20 |
|  | *Me1-*TNFR | HE609052 | nd |  |  |  |  |
|  | *Me2-*TNFR | HE609053 | nd |  |  |  |  |
|  | *Me3*-TNFR | HE609054 | [HM004082.1](http://www.ncbi.nlm.nih.gov/nucleotide/294987166?report=genbank&log$=nucltop&blast_rank=1&RID=3Y56N6TG016) | *M. trossulus* | monocyte-derived macrophages (MDM)-like protein | 13% | 3,00E-04 |
|  | *Me1*-TRAF | HE609055 | nd |  |  |  |  |
|  | *Me2*-TRAF | HE609056 | nd |  |  |  |  |
|  | *Me3*-TRAF | HE609057 | nd |  |  |  |  |
|  | *Me4*-TRAF | HE609058 | nd |  |  |  |  |
|  | *Me1*-I*κ*B | HE609059 | nd |  |  |  |  |
|  | *Me2*-I*κ*B | HE609060 | nd |  |  |  |  |
|  | IKK | HE609061 | nd |  |  |  |  |
|  | *Me1*-caspase 8 | HE609062 | [FJ937785.1](http://www.ncbi.nlm.nih.gov/nucleotide/284159257?report=genbank&log$=nucltop&blast_rank=1&RID=3V328EGV011) | *M. californianus* | casp 8 | 39% | 9,00E-37 |
|  | *Me2*-caspase 8 | HE609063 | [FJ937784.1](http://www.ncbi.nlm.nih.gov/nucleotide/284159255?report=genbank&log$=nucltop&blast_rank=1&RID=3V4RKB5T016) | *M. californianus* | casp-8-like | 100% | 4,00E-90 |
|  | ECSIT | HE609064 | nd |  |  |  |  |
|  | IRAK 4 | HE609065 | [GQ454915.1](http://www.ncbi.nlm.nih.gov/nucleotide/258677221?report=genbank&log$=nucltop&blast_rank=1&RID=3T49BHJ6011) | *M. galloprovincialis* | extracellular regulated protein kinase | 6% | 1,00E-05 |
|  | Jun/AP1 | HE609066 | nd |  |  |  |  |
|  | *Me1*-LBP | HE609067 | nd |  |  |  |  |
|  | *Me2*-LBP | HE609068 | nd |  |  |  |  |
|  | *Me3*-LBP | HE609069 | nd |  |  |  |  |
|  | *Me4*-LBP | HE609070 | nd |  |  |  |  |
|  | Myd88 | HE609071 | nd |  |  |  |  |
|  | p38 MAPK | HE609072 | [GQ454914.1](http://www.ncbi.nlm.nih.gov/nucleotide/258677219?report=genbank&log$=nucltop&blast_rank=1&RID=62KV81ST013) | *M.galloprovincialis* | c-Jun N-terminal kinase JNK | 21% | 5,00E-34 |
|  | SARM | HE609073 | nd |  |  |  |  |
|  | TOLLIP | HE609074 | nd |  |  |  |  |
|  | TRAF6 | HE609075 | nd |  |  |  |  |
|  | TRAM (TICAM 2) | HE609076 | nd |  |  |  |  |
|  | FADD | HE609077 | [FJ937785.1](http://www.ncbi.nlm.nih.gov/nucleotide/284159257?report=genbank&log$=nucltop&blast_rank=1&RID=3VNWFU6501P) | *M. californianus* | casp 8 | 17% | 3,00E-06 |
|  | MDA-5 | HE609078 | nd |  |  |  |  |
|  | DDX58 (RIG-I) | HE609079 | nd |  |  |  |  |
|  | *Me1*-PGRP | HE609080 | nd |  |  |  |  |
|  | *Me2*-PGRP | HE609081 | nd |  |  |  |  |
|  | *Me3-*PGRP | HE609082 | nd |  |  |  |  |
|  | *Me4*-PGRP | HE609083 | nd |  |  |  |  |
|  | *Me5*-PGRP | HE609084 | nd |  |  |  |  |
|  | *Me6-*PGRP | HE609085 | nd |  |  |  |  |
|  | *Me7-*PGRP | HE609086 | nd |  |  |  |  |
|  | *Me8*-PGRP | HE609087 | nd |  |  |  |  |
|  | *Me1*-GNBP | HE609088 | nd |  |  |  |  |
|  | *Me2*-GNBP | HE609089 | nd |  |  |  |  |
|  | *Me3*-GNBP | HE609090 | nd |  |  |  |  |
|  | TBK1 | HE609091 | nd |  |  |  |  |
|  | TAK1 | HE609092 | nd |  |  |  |  |
|  | A20 | HE609093 | nd |  |  |  |  |
|  | JAK 2 | HE609094 | [DQ158074.1](http://www.ncbi.nlm.nih.gov/nucleotide/74418623?report=genbank&log$=nucltop&blast_rank=1&RID=40AGHPSG011) | *M. galloprovincialis* | stress responsive kinase (KRS) | 8% | 4,00E-05 |
|  | *Me1*-STAT | HE609095 | nd |  |  |  |  |
|  | *Me2-*STAT | HE609096 | nd |  |  |  |  |
|  | *Me3*-STAT | HE609097 | nd |  |  |  |  |
|  | *Me1-*SOCS | HE609098 | nd |  |  |  |  |
|  | *Me2*-SOCS | HE609099 | nd |  |  |  |  |
| Cytokines | IL-16 | HE609100 | nd |  |  |  |  |
|  | *Me1*-IL-17 | HE609101 | nd |  |  |  |  |
|  | *Me2*-IL-17 | HE609102 | nd |  |  |  |  |
|  | *Me1-*MIF | HE609103 | nd |  |  |  |  |
|  | *Me2-*MIF | HE609104 | nd |  |  |  |  |
|  | *Me3-*MIF | HE609105 | nd |  |  |  |  |
|  | *Me4-*MIF | HE609106 | nd |  |  |  |  |
|  | *Me5-*MIF | HE609107 | nd |  |  |  |  |
| Complement system | *Me1-*C3 | HE609108 | nd |  |  |  |  |
|  | *Me2*-C3 | HE609109 | nd |  |  |  |  |
|  | C6 | HE609110 | nd |  |  |  |  |
|  | DMBT1 | HE609111 | [EF080941.1](http://www.ncbi.nlm.nih.gov/nucleotide/122056401?report=genbank&log$=nucltop&blast_rank=1&RID=48X9ATA3011) | *M. galloprovincialis* | putative uromodulin | 15% | 3,00E-122 |
|  | ATG3 | HE609112 | nd |  |  |  |  |
|  | *Me1-*ATG4B | HE609113 | nd |  |  |  |  |
|  | *Me2-*ATG4B | HE609114 | nd |  |  |  |  |
|  | ATG4C | HE609115 | nd |  |  |  |  |
|  | ATG5 | HE609116 | nd |  |  |  |  |
|  | ATG7 | HE609117 | nd |  |  |  |  |
| Autophagy / Apoptosis | ATG 8 (LC3) | HE609118 | [AJ308548.1](http://www.ncbi.nlm.nih.gov/nucleotide/15384733?report=genbank&log$=nucltop&blast_rank=1&RID=4B0S02KD016) | *M. edulis* | endo-1,4-β-D-glucanase | 17% | 6,00E-38 |
|  | ATG9 | HE609119 | nd |  |  |  |  |
|  | ATG12 | HE609120 | nd |  |  |  |  |
|  | ATG16 | HE609121 | nd |  |  |  |  |
|  | Beclin1 | HE609122 | nd |  |  |  |  |
|  | mTOR | HE609123 | nd |  |  |  |  |
|  | AIFM1 | HE609124 | nd |  |  |  |  |
|  | AIFM3 (AFL) | HE609125 | nd |  |  |  |  |
|  | Bak | HE609126 | nd |  |  |  |  |
|  | Bax | HE609127 | nd |  |  |  |  |
|  | *Me1*-BCL2 | HE609128 | nd |  |  |  |  |
|  | *Me2-*BCL2 | HE609129 | nd |  |  |  |  |
|  | BCLxL | HE609130 | nd |  |  |  |  |
|  | initiator caspase with CARD domain | HE609131 | [FJ937784.1](http://www.ncbi.nlm.nih.gov/nucleotide/284159255?report=genbank&log$=nucltop&blast_rank=1&RID=4BA6A6UF016) | *M. californianus* | casp 8-like | 23% | 6,00E-12 |
