# Supplementary material for: Massively Parallel RNA Sequencing Identifies a Complex Immune Gene Repertoire in the lophotrochozoan Mytilus edulis
Source: PLoS One. 2012 Mar 20;7(3):e33091. doi: 10.1371/journal.pone.0033091 (PMC3308963; doi:10.1371/journal.pone.0033091)
Supplement: Table S5 — TNF related M. edulis transcripts with conserved TNF or TNFR domains identified by PROSITE. (DOC) [file pone.0033091.s008.doc]

| *M. edulis*  Accession | Contig length (bp) | Nr. of reads | longest ORF (aa) | Domains identified |
| --- | --- | --- | --- | --- |
| TNF superfamiliy |  |  |  |  |
| HE610071 | 885 | 9 | 73 | TNF2, TNF1 |
| HE610065 | 2749 | 153 | 288 | TNF2,TM |
| HE610066 | 1176 | 22 | 306 | TNF2, TM |
| HE610070 | 418 | 3 | 139 | TNF2 |
| HE610067 | 1930 | 172 | 272 | TNF2, TM |
| HE610073 | 1466 | 38 | 283 | TNF2, TM |
| HE610059 | 1656 | 41 | 414 | TNF2, TM |
| HE610069 | 1683 | 20 | 212 | TNF2 |
| HE610064 | 1221 | 17 | 302 | TNF2, TM |
| HE610061 | 1593 | 75 | 263 | TNF2, TM |
| HE610060 | 2568 | 77 | 171 | TNF2 |
| HE610063 | 1498 | 20 | 339 | TNF2, TM |
| TNF receptor |  |  |  |  |
| HE609052 | 1973 | 81 | 331 | TNFR,TM, DEATH |
| HE609053 | 1245 | 140 | 335 | 2x TNFR, TM, DEATH |
| HE609054 | 1564 | 236 | 341 | TNFR,TM, DEATH |
| HE651930 | 875 | 11 | 292 | 2x TNFR |
| HE610074 | 651 | 13 | 178 | 2x TNFR |
| HE610075 | 2760 | 169 | 282 | 2x TNFR |
| HE610062 | 849 | 25 | 140 | TNFR |
| HE610072 | 509 | 19 | 121 | TNFR |
| HE610068 | 422 | 3 | 103 | 2x TNFR |
