# Supplementary material for: Massively Parallel RNA Sequencing Identifies a Complex Immune Gene Repertoire in the lophotrochozoan Mytilus edulis
Source: PLoS One. 2012 Mar 20;7(3):e33091. doi: 10.1371/journal.pone.0033091 (PMC3308963; doi:10.1371/journal.pone.0033091)
Supplement: Table S6 — M. edulis contigs with high similarity to LPS induced TNF-alpha factor (LITAF) - like proteins of other organisms. (DOC) [file pone.0033091.s009.doc]

| Contig information | |  | Best blast hit (UniprotKB/Swissprot) LITAF-like homolog | | | | |
| --- | --- | --- | --- | --- | --- | --- | --- |
| *M. edulis* Accession | Contig length (bp) | Nr. of reads | Accession | Species | Length | % Identity | E-value |
| HE609137 | 347 | 2 | Q5U2U6LITFL | *Rattus norvegicus* | 208 | 43.0 | 7.0×10^-10^ |
| HE609140 | 345 | 3 | P0C0T0LITAF | *Rattus norvegicus* | 161 | 48.0 | 5.0×10^-6^ |
| HE609148 | 557 | 3 | Q5U2U6LITFL | *Rattus norvegicus* | 208 | 43.0 | 2.0×10^-7^ |
| HE609150 | 481 | 6 | Q9JLJ0LITAF | *Mus musculus* | 161 | 40.0 | 1.0×10^-15^ |
| HE609135 | 626 | 7 | Q6GMG8LITAF | *Danio rerio* | 163 | 31.0 | 2.0×10^-7^ |
| HE609153 | 405 | 9 | Q54HX8LITAH | *Dictyostelium discoideum* | 181 | 50.0 | 5.0×10^-13^ |
| HE609154 | 478 | 9 | Q54HX8LITAH | *Dictyostelium discoideum* | 181 | 48.0 | 4.0×10^-22^ |
| HE609143 | 577 | 11 | Q9H305LITFL | *Homo sapiens* | 208 | 45.0 | 8.0×10^-14^ |
| HE609149 | 928 | 20 | Q54HX8LITAH | *Dictyostelium discoideum* | 181 | 41.0 | 9.0×10^-29^ |
| HE609147 | 1063 | 23 | Q9H305LITFL | *Homo sapiens* | 208 | 38.0 | 9.0×10^-27^ |
| HE609152 | 1068 | 23 | Q6GMG8LITAF | *Danio rerio* | 163 | 42.0 | 2.0×10^-22^ |
| HE609146 | 749 | 24 | Q8QGW7LITAF | *Gallus gallus* | 148 | 45.0 | 4.0×10^-20^ |
| HE609151 | 1627 | 33 | Q8QGW7LITAF | *Gallus gallus* | 148 | 49.0 | 4.0×10^-23^ |
| HE609144 | 1213 | 35 | P0C0T0LITAF | *Rattus norvegicus* | 161 | 32.0 | 1.0×10^-16^ |
| HE609136 | 1040 | 43 | Q8QGW7LITAF | *Gallus gallus* | 148 | 45.0 | 2.0×10^-23^ |
| HE609141 | 850 | 66 | Q54HX8LITAH | *Dictyostelium discoideum* | 181 | 41.0 | 1.0×10^-21^ |
| HE609138 | 1271 | 69 | Q54HX8LITAH | *Dictyostelium discoideum* | 181 | 38.0 | 7.0×10^-20^ |
| HE609132 | 874 | 81 | Q58D45LITFL | *Bos taurus* | 208 | 35.0 | 4.0×10^-15^ |
| HE609139 | 2565 | 113 | Q54HX8LITAH | *Dictyostelium discoideum* | 181 | 42.0 | 5.0×10^-29^ |
| HE609142 | 1200 | 128 | Q6P828LITAF | *Xenopus tropicalis* | 148 | 39.0 | 3.0×10^-16^ |
| HE609134 | 975 | 132 | Q9DB75LITFL | *Mus musculus* | 208 | 42.0 | 1.0×10^-21^ |
| HE609145 | 1388 | 167 | Q8QGW7LITAF | *Gallus gallus* | 148 | 45.0 | 4.0×10^-23^ |
| HE609047 | 1523 | 261 | Q8QGW7LITAF | *Gallus gallus* | 148 | 48.0 | 1.0×10^-23^ |
| HE609133 | 1245 | 272 | Q54HX8LITAH | *Dictyostelium discoideum* | 181 | 41.0 | 2.0×10^-21^ |
