# Supplementary material for: Massively Parallel RNA Sequencing Identifies a Complex Immune Gene Repertoire in the lophotrochozoan Mytilus edulis
Source: PLoS One. 2012 Mar 20;7(3):e33091. doi: 10.1371/journal.pone.0033091 (PMC3308963; doi:10.1371/journal.pone.0033091)
Supplement: Table S7 — Antimicrobial peptides identified in M. edulis . (DOC) [file pone.0033091.s010.doc]

| Gene | Contig information | |  | Best blast hit (UniprotKB/Swissprot) | |  |  |  |  |  |
| --- | --- | --- | --- | --- | --- | --- | --- | --- | --- | --- |
|  | *M. edulis*  Accession | Contig length (bp) | Nr. of reads | Accession | Protein | Species | Length | Identity | Score | E-value |
| Mytimycin | HE609161 | 530 | 48 | C1K9I9C1K9I9 | Mytimycin | *Mytilus galloprovincialis* | 152 | 90.0% | 536 | 4.0×10^-53^ |
|  | HE609158 | 404 | 53 | C1K9I9C1K9I9 | Mytimycin | *M.galloprovincialis* | 152 | 90.0% | 536 | 3.0×10^-53^ |
|  | HE609157 | 718 | 49 | C1K9I9C1K9I9 | Mytimycin | *M.galloprovincialis* | 152 | 90.0% | 789 | 4.0×10^-82^ |
|  | HE609159 | 1164 | 277 | C1K9I9C1K9I9 | Mytimycin | *M.galloprovincialis* | 152 | 91.0% | 236 | 1.0×10^-17^ |
|  | HE609160 | 603 | 52 | C1K9I9C1K9I9 | Mytimycin | *M.galloprovincialis* | 152 | 58.0% | 491 | 9.0×10^-48^ |
|  | HE610032 | 763 | 2073 | C1K9I9C1K9I9 | Mytimycin | *M.galloprovincialis* | 152 | 95.0% | 400 | 2.0×10^-57^ |
|  | HE609156 | 694 | 212 | C1K9I9C1K9I9 | Mytimycin | *M.galloprovincialis* | 152 | 69.0% | 596 | 9.0×10^-60^ |
| Myticin | HE609875 | 749 | 2513 | P82103MYNA | Myticin A | *M.galloprovincialis* | 96 | 95.0% | 515 | 2.0×10^-50^ |
|  | HE609165 | 435 | 53 | P82103MYNA | Myticin A | *M.galloprovincialis* | 96 | 100.0% | 148 | 3.0×10^-8^ |
|  | HE609164 | 792 | 671 | A7XP66A7XP66 | Myticin B | *M.galloprovincialis* | 96 | 97.0% | 519 | 9.0×10^-51^ |
|  | HE609163 | 1355 | 1095 | A7DWV3A7DWV3 | Myticin C | *M.galloprovincialis* | 100 | 98.0% | 526 | 3.0×10^-51^ |
|  | HE609166 | 582 | 97 | A7DWU0A7DWU0 | Myticin C | *M.galloprovincialis* | 100 | 86.0% | 240 | 2.0×10^-30^ |
| Defensin | HE609169 | 740 | 116 | Q9U6U0Q9U6U0 | Antimicrobial peptide MGD2b | *M.galloprovincialis* | 82 | 79.0% | 382 | 6.0×10^-35^ |
|  | HE609171 | 507 | 63 | Q9Y0A9Q9Y0A9 | MGD2 antimicrobial peptide | *M.galloprovincialis* | 81 | 36.0% | 148 | 1.0×10^-7^ |
|  | HE609168 | 479 | 55 | Q9Y0A9Q9Y0A9 | MGD2 antimicrobial peptide | *M.galloprovincialis* | 81 | 50.0% | 162 | 8.0×10^-10^ |
|  | HE609170 | 496 | 64 | Q9Y0A9Q9Y0A9 | MGD2 antimicrobial peptide | *M.galloprovincialis* | 81 | 50.0% | 157 | 3.0×10^-9^ |
|  | HE609174 | 962 | 888 | Q9Y0A9Q9Y0A9 | MGD2 antimicrobial peptide | *M.galloprovincialis* | 81 | 46.0% | 153 | 4.0×10^-8^ |
|  | HE609173 | 588 | 41 | C4NYB8C4NYB8_ | Defensin | *Crassostrea gigas* | 65 | 45.0% | 152 | 2.0×10^-8^ |
|  | HE609172 | 993 | 348 | Q9Y0A9Q9Y0A9 | MGD2 antimicrobial peptide | *M.galloprovincialis* | 81 | 36.0% | 148 | 1.0×10^-7^ |
|  | HE609167 | 448 | 50 | Q9U6U0Q9U6U0 | Antimicrobial peptide MGD2b | *M.galloprovincialis* | 82 | 52.0% | 104 | 4.0×10^-3^ |
| Mytilin | HE609175 | 838 | 20708 | Q9Y0B1Q9Y0B1 | Mytilin B | *M.galloprovincialis* | 103 | 100.0% | 555 | 7.0×10^-55^ |
|  | HE609178 | 689 | 346 | Q9Y0B1Q9Y0B1 | Mytilin B | *M.galloprovincialis* | 103 | 82.0% | 471 | 3.0×10^-45^ |
|  | HE609187 | 210 | 369 | Q9Y0B1Q9Y0B1 | Mytilin B | *M.galloprovincialis* | 103 | 92.0% | 166 | 3.0×10^-10^ |
|  | HE609185 | 188 | 62 | Q9Y0B1Q9Y0B1 | Mytilin B | *M.galloprovincialis* | 103 | 100.0% | 136 | 8.0×10^-7^ |
|  | HE609176 | 611 | 8527 | Q5XWD7Q5XWD7 | Mytilin C | *M.trossulus* | 99 | 97.0% | 528 | 5.0×10^-52^ |
|  | HE609182 | 413 | 86 | Q5XWD7Q5XWD7 | Mytilin C | *M.trossulus* | 99 | 97.0% | 221 | 1.0×10^-16^ |
|  | HE609186 | 361 | 522 | Q5XWD7Q5XWD7 | Mytilin C | *M.trossulus* | 99 | 85.0% | 429 | 9.0×10^-41^ |
|  | HE609184 | 545 | 371 | Q5XWD7Q5XWD7 | Mytilin C | *M.trossulus* | 99 | 100.0% | 534 | 7.0×10^-53^ |
|  | HE609179 | 820 | 699 | B3VT96B3VT96 | Mytilin D | *M.galloprovincialis* | 97 | 98.0% | 514 | 4.0×10^-50^ |
|  | HE609183 | 443 | 65 | B3VT96B3VT96 | Mytilin D | *M.galloprovincialis* | 97 | 98.0% | 514 | 4.0×10^-50^ |
|  | HE609180 | 494 | 274 | B3VT96B3VT96 | Mytilin D | *M.galloprovincialis* | 97 | 90.0% | 473 | 7.0×10^-46^ |
|  | HE609177 | 416 | 12 | D3JX72D3JX72 | Mytilin-3 | *M.coruscus* | 101 | 58.0% | 196 | 9.0×10^-14^ |
|  | HE609181 | 507 | 26 | D3JX72D3JX72 | Mytilin-3 | *M.coruscus* | 101 | 47.0% | 164 | 5.0×10^-10^ |
| Big defensin | HE609188 | 1231 | 227 | Q86QN6BDEF | Big defensin | *Branchiostoma belcheri* | 117 | 44.0% | 265 | 5.0×10^-21^ |
|  | HE609190 | 458 | 52 | Q86QN6BDEF | Big defensin | *B. belcheri* | 117 | 44.0% | 262 | 2.0×10^-21^ |
|  | HE609189 | 472 | 26 | Q86QN6BDEF | Big defensin | *B. belcheri* | 117 | 43.0% | 233 | 5.0×10^-18^ |
|  | HE609191 | 583 | 50 | Q86QN6BDEF | Big defensin | *B. belcheri* | 117 | 43.0% | 220 | 2.0×10^-16^ |
|  | HE609192 | 430 | 7 | Q86QN6BDEF | Big defensin | *B. belcheri* | 117 | 45.0% | 232 | 6.0×10^-18^ |
