# Supplementary material for: Massively Parallel RNA Sequencing Identifies a Complex Immune Gene Repertoire in the lophotrochozoan Mytilus edulis
Source: PLoS One. 2012 Mar 20;7(3):e33091. doi: 10.1371/journal.pone.0033091 (PMC3308963; doi:10.1371/journal.pone.0033091)
Supplement: Table S8 — 19 MACPF domain bearing contigs of M. edulis . 5 of these fragments showed similarity to macrophage expressed gene-1, and 3 to apextrin, while 11 fragments could not be annotated. Proteins containing the MACPF domain but lacking other domains of the MAC complex members were reported in several other lower invertebrates (for review see, [108]). Some are known to belong to genes with strong hemolytic function like the hemolytic toxins of the nematocysts of the sea anemone Phyllodiscus semoni [108], and are therefore put in close relation to the vertebrate MAC members. Whether the 11 non-annotated MACPF containing fragments of M. edulis belong to the complement systems needs further sequence and functional analysis. (DOC) [file pone.0033091.s011.doc]

| *M. edulis* accession | Length (bp) | Nr. of reads | Annotation |
| --- | --- | --- | --- |
| HE610076 | 2668 | 519 | apextrin-like |
| HE610077 | 1598 | 28 | apextrin-like |
| HE610083 | 771 | 10 | apextrin-like |
| HE610079 | 2503 | 124 | Macrophage-expressed gene 1 protein |
| HE610085 | 2372 | 1104 | Macrophage-expressed gene 1 protein |
| HE610091 | 2623 | 233 | Macrophage-expressed gene 1 protein |
| HE610092 | 1826 | 14 | Macrophage-expressed gene 1 protein |
| HE610094 | 2248 | 71 | Macrophage-expressed gene 1 protein |
| HE610078 | 1782 | 173 | N |
| HE610080 | 364 | 2 | N |
| HE610081 | 1852 | 82 | N |
| HE610082 | 1721 | 23 | N |
| HE610084 | 1882 | 69 | N |
| HE610086 | 2173 | 121 | N |
| HE610087 | 1310 | 21 | N |
| HE610088 | 1769 | 42 | N |
| HE610089 | 1790 | 55 | N |
| HE610090 | 2021 | 53 | N |
| HE610093 | 1780 | 46 | N |
