# Supplementary material for: Massively Parallel RNA Sequencing Identifies a Complex Immune Gene Repertoire in the lophotrochozoan Mytilus edulis
Source: PLoS One. 2012 Mar 20;7(3):e33091. doi: 10.1371/journal.pone.0033091 (PMC3308963; doi:10.1371/journal.pone.0033091)
Supplement: Table S9 — Caspase-like contigs of M. edulis . (DOC) [file pone.0033091.s012.doc]

| *M. edulis* accession | Length (bp) | Nr. of reads | Domains identified |
| --- | --- | --- | --- |
| HE609959 | 728 | 2 | BIR, Peptidase_C14 |
| HE609971 | 2030 | 63 | CARD, Peptidase_C14 |
| HE609955 | 2070 | 44 | CARD, Peptidase_C14 |
| HE609131 | 1485 | 43 | CARD, Peptidase_C14 |
| HE609980 | 1657 | 28 | CARD, Peptidase_C14 |
| HE609978 | 1282 | 16 | CARD, Peptidase_C14 |
| HE609051 | 3538 | 226 | Peptidase_C14 |
| HE609954 | 1464 | 89 | Peptidase_C14 |
| HE609969 | 1820 | 81 | Peptidase_C14 |
| HE609951 | 1413 | 59 | Peptidase_C14 |
| HE609946 | 2198 | 43 | Peptidase_C14 |
| HE609958 | 1413 | 40 | Peptidase_C14 |
| HE609981 | 2251 | 38 | Peptidase_C14 |
| HE609953 | 1234 | 28 | Peptidase_C14 |
| HE609949 | 532 | 26 | Peptidase_C14 |
| HE609944 | 1231 | 25 | Peptidase_C14 |
| HE609968 | 1564 | 22 | Peptidase_C14 |
| HE609950 | 490 | 21 | Peptidase_C14 |
| HE609947 | 1181 | 20 | Peptidase_C14 |
| HE609957 | 1163 | 16 | Peptidase_C14 |
| HE609975 | 1358 | 14 | Peptidase_C14 |
| HE609948 | 802 | 13 | Peptidase_C14 |
| HE609937 | 699 | 12 | Peptidase_C14 |
| HE609952 | 1159 | 10 | Peptidase_C14 |
| HE609933 | 469 | 9 | Peptidase_C14 |
| HE609062 | 782 | 9 | Peptidase_C14 |
| HE609962 | 853 | 8 | Peptidase_C14 |
| HE609956 | 616 | 7 | Peptidase_C14 |
| HE609966 | 410 | 6 | Peptidase_C14 |
| HE609967 | 588 | 6 | Peptidase_C14 |
| HE609974 | 698 | 6 | Peptidase_C14 |
| HE609934/HE609941* | 556 | 5 | Peptidase_C14 |
| HE609063 | 447 | 5 | Peptidase_C14 |
| HE609938 | 630 | 4 | Peptidase_C14 |
| HE609940 | 599 | 4 | Peptidase_C14 |
| HE609942 | 346 | 4 | Peptidase_C14 |
| HE609961 | 514 | 4 | Peptidase_C14 |
| HE609972 | 655 | 4 | Peptidase_C14 |
| HE609945 | 500 | 3 | Peptidase_C14 |
| HE609963 | 416 | 3 | Peptidase_C14 |
| HE609965 | 461 | 3 | Peptidase_C14 |
| HE609976 | 505 | 3 | Peptidase_C14 |
| HE609977 | 702 | 3 | Peptidase_C14 |
| HE609935 | 383 | 2 | Peptidase_C14 |
| HE609936 | 280 | 2 | Peptidase_C14 |
| HE609939 | 542 | 2 | Peptidase_C14 |
| HE609943 | 486 | 2 | Peptidase_C14 |
| HE609960 | 524 | 2 | Peptidase_C14 |
| HE609964 | 462 | 2 | Peptidase_C14 |
| HE609970 | 240 | 2 | Peptidase_C14 |
| HE609973 | 297 | 2 | Peptidase_C14 |
| HE609979 | 475 | 2 | Peptidase_C14 |

*contigs can be aligned to a 556bp long transcript with min 20bp overlap and 90% identity
